# Enhancer of zeste homolog 2 promotes renal fibrosis after acute kidney injury by inducing epithelial-mesenchymal transition and activation of M2 macrophage polarization

**DOI:** 10.1038/s41419-023-05782-4

**Published:** 2023-04-07

**Authors:** Xun Zhou, Hui Chen, Yan Hu, Xiaoyan Ma, Jinqing Li, Yingfeng Shi, Min Tao, Yi Wang, Qin Zhong, Danying Yan, Shougang Zhuang, Na Liu

**Affiliations:** 1 Department of Nephrology, Shanghai East Hospital, Tongji University School of Medicine, Shanghai, China; 2 Department of Medicine, Rhode Island Hospital and Alpert Medical School, Brown University, Providence, RI USA

**Keywords:** Chronic kidney disease, Acute kidney injury, Genetics research, Post-translational modifications, Clinical epigenetics

## Abstract

Long-term follow-up data indicates that 1/4 patients with acute kidney injury (AKI) will develop to chronic kidney disease (CKD). Our previous studies have demonstrated that enhancer of zeste homolog 2 (EZH2) played an important role in AKI and CKD. However, the role and mechanisms of EZH2 in AKI-to-CKD transition are still unclear. Here, we demonstrated EZH2 and H3K27me3 highly upregulated in kidney from patients with ANCA-associated glomerulonephritis, and expressed positively with fibrotic lesion and negatively with renal function. Conditional EZH2 deletion or pharmacological inhibition with 3-DZNeP significantly improved renal function and attenuated pathological lesion in ischemia/reperfusion (I/R) or folic acid (FA) mice models (two models of AKI-to-CKD transition). Mechanistically, we used CUT & Tag technology to verify that EZH2 binding to the PTEN promoter and regulating its transcription, thus regulating its downstream signaling pathways. Genetic or pharmacological depletion of EZH2 upregulated PTEN expression and suppressed the phosphorylation of EGFR and its downstream signaling ERK1/2 and STAT3, consequently alleviating the partial epithelial-mesenchymal transition (EMT), G2/M arrest, and the aberrant secretion of profibrogenic and proinflammatory factors in vivo and vitro experiments. In addition, EZH2 promoted the EMT program induced loss of renal tubular epithelial cell transporters (OAT1, ATPase, and AQP1), and blockade of EZH2 prevented it. We further co-cultured macrophages with the medium of human renal tubular epithelial cells treated with H_2_O_2_ and found macrophages transferred to M2 phenotype, and EZH2 could regulate M2 macrophage polarization through STAT6 and PI3K/AKT pathways. These results were further verified in two mice models. Thus, targeted inhibition of EZH2 might be a novel therapy for ameliorating renal fibrosis after acute kidney injury by counteracting partial EMT and blockade of M2 macrophage polarization.

## Introduction

Acute kidney injury (AKI) is a group of multidisciplinary clinical emergencies with a mortality rate approaching 50% [1–3]. AKI can lead to complete or incomplete tubular repair depending on the severity of the injury. After mild injury, the kidney can return to its normal structural and functional state, but severe or repeated injury is often accompanied by the development of renal fibrosis, and long-term follow-up data show that about 1/4 of patients with AKI will develop to chronic kidney disease (CKD) [4–6]. The renal fibrosis is characterized by activation and proliferation of fibroblast, inflammatory cell infiltration, and tubular cell atrophy [7]. Historical data suggests that the progression of renal fibrosis might be the result of the transformation of epithelial cells into cells with mesenchymal characteristics, known as epithelial-mesenchymal transition (EMT) [8]. Epithelial cell arrest in G2/M phase is a functional consequence of EMT [8]. After injury, renal tubular epithelial cells arrest at G2/M phase, leading to secretion of various profibrotic cytokines, proinflammatory cytokines, and growth factors [9–11]. Inflammatory infiltration is also considered to be an important driver of AKI progression and there is increasing evidence that activation of M2 macrophages is involved in renal fibrosis [12, 13]. Well-balanced crosstalk between tubular epithelial cells and mesenchymal cells or inflammatory cells is essential to maintain normal renal function.

Epidermal growth factor receptor (EGFR) signaling is involved in regulation of multiple biological processes [14]. Our previous studies have revealed that EGFR signaling induces renal fibrosis by promoting epithelial cell G2/M arrest and production of inflammatory cytokines/chemockines [10]. Phosphorylation of EGFR also induces the activation of signal transducer and activator of transcription 3 (STAT3) and extracellular signal-regulated kinase 1 and 2 (ERK1/2) signaling pathways [15]. In contrast, phosphatase and tensin homolog (PTEN) can interfere with the activation of multiple pro-fibrotic signaling pathways, thereby inhibiting tissue fibrosis [16].

There is increasing evidence that macrophage infiltration plays an important role in acute and chronic kidney disease [17]. Following recruitment to the damaged kidney, macrophages can be broadly classified into two different subtypes: classically activated (M1) and alternatively activated (M2) macrophages [18]. M2 macrophages promote tissue repair and exhibit anti-inflammatory effects [19]. However, in face of sustained injury, continuous infiltration of M2 macrophages leads to the activation of fibroblasts through the release of transforming growth factor-beta, platelet-derived growth factor, and vascular endothelium growth factor [20], resulting in irreversible fibrosis and renal tissue destruction [21]. Further studies in animal models of CKD, such as diabetic nephropathy [22] and unilateral ureteral obstruction (UUO) [23, 24], activation of the M2 phenotype leads to glomerulosclerosis, tubular interstitial fibrosis, and eventually organ failure. Activation of signal transducers and activators of transcription 6 (STAT6) [25–27] and phosphatidylinositol-3-kinase (PI3K)/AKT pathways [28, 29] can promote M2 macrophage polarization. Furthermore, latest research has revealed that M2 macrophage polarization is regulated by epigenetics [30].

Epigenetics refers to heritable changes in gene function that result in phenotypic changes without changes in the DNA sequence [31]. This mode of gene regulation can occur at the levels of DNA modification, protein modification, and non-coding RNA regulation. Enhancer of zeste homolog 2 (EZH2), a recently discovered histone methyltransferase, induces trimethylation at lysine 27 of histone H3 (H3K27me3), and plays an important role in renal diseases [32]. Previous studies of our research group have revealed that EZH2 is highly expressed in the kidney of AKI mice [33] and can sustainably promote the progression of renal fibrosis in CKD mice [16].

In this research, we examined the expressions of EZH2 and H3K27me3 in the kidneys of patients with ANCA-associated glomerulonephritis (AAGN), and the correlation between EZH2 and Masson’s trichrome-positive area, renal function as well as 24-h urine protein. To further investigate the role and mechanism of EZH2 in AKI-to-CKD transition, we constructed EZH2 conditional knockout mice (EZH2-cKO) and established two AKI-to-CKD transition models by ischemia/reperfusion (I/R) or folic acid (FA). In addition, we examined pharmacological or genetic inhibition of EZH2 on H_2_O_2_-induced EMT in cultured human tubular epithelial cells (HK2) and M2 macrophage polarization in mouse macrophage cells (RAW264.7) co-cultured with the medium of HK2 cells treated with H_2_O_2_. We hypothesized that EZH2 might play a vital role in the progression from AKI to CKD by regulating EMT in renal tubular epithelial cells and M2 macrophage polarization.

## Materials and methods

Additional details for all methods were provided in the Supplementary Materials.

### Human renal biopsy samples

Renal biopsies had been performed as part of routine clinical diagnostic investigation and collected as described in Supplementary Materials (Table S1). For the detection of EZH2 and H3K27me3 expression levels, we enrolled 9 patients who were diagnosed with AAGN by renal biopsy. Control samples (*n* = 9) were obtained from the healthy kidney tissues of individuals who underwent tumor nephrectomies without diabetes or chronic renal disease. The samples of renal biopsies were obtained from Department of Nephrology, Shanghai East Hospital affiliated with Tongji University from January 2017 to May 2022. The details of human renal biopsy samples were provided in the Supplementary Materials.

### Animals and treatment

Two mice models of AKI-to-CKD transition induced by I/R or FA were established. The I/R model was established by clipping the bilateral renal arteries of mice for 30 min, reperfusion of the kidneys was visually confirmed after removal of the clamps [34]. The animals were observed and fed for 4 weeks after operation. The FA model was established by one intraperitoneal dose of folic acid (250 mg/kg) dissolved in 300 mM NaHCO_3_ [35], while the control group was injected with an identical voluminal vehicle (300 mM NaHCO_3_, i.p.) for 4 weeks. To investigate the effect of 3-DZNeP on renal fibrosis, mice in I/R or FA model were intraperitoneally injected with 3-DZNeP (1 mg/kg) in saline every day. The details of animals and treatment were provided in the Supplementary Materials.

### Cell culture

HK2 cells were purchased from American Type Culture Collection (Manassas, VA, USA) and cultured in Dulbecco’s modified Eagle’s medium (DMEM) with F12 containing 10% fetal bovine serum (FBS), 1% penicillin, and streptomycin in an atmosphere of 5% CO_2_, and 95% air at 37 °C. To examine the anti-fibrotic effect of 3-DZNeP in H_2_O_2_-induced EMT in vitro, HK2 cells were starved for 24 h with DMEM/F12 containing 0.5% FBS and then exposed to H_2_O_2_ (0.5 mM) in the presence of 3-DZNeP (5 μM) or EZH2 siRNA for 24 h. Mouse macrophage cells (RAW264.7) were purchased from American Type Culture Collection (Manassas, VA, USA) and cultured in DMEM with 1640 containing 10% FBS, 1% penicillin, and streptomycin in an atmosphere of 5% CO_2_, and 95% air at 37 °C. To examine the effect of 3-DZNeP in M2 macrophage polarization in vitro, RAW264.7 cells were cultured with or without 10% (vol/vol) pre-collected cell culture media from HK2 cells treated as described above in the presence of 3-DZNeP (5 μM) or EZH2 siRNA for 24 h. Then, cells were harvested for further immunoblot analysis or immunofluorescent staining. All of the in vitro experiments were repeated for at least three times.

## Results

### EZH2 is highly upregulated in kidney of patients with ANCA-associated glomerulonephritis and correlated positively with Masson’s trichrome-positive area and negatively with eGFR

**Fig. 1:**
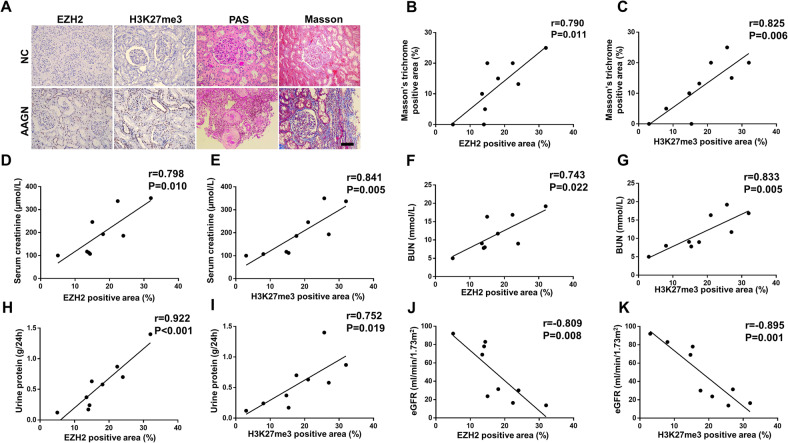
EZH2 is highly upregulated in kidney of patients with ANCA-associated glomerulonephritis and correlated positively with Masson’s trichrome-positive area and negatively with eGFR. **A** The photomicrographs of EZH2 and H3K27me3 immunohistochemical staining, PAS, and Masson’s trichrome staining in normal paracancerous tissue from renal carcinoma patient (normal control, NC) and renal cortical tissue from patients with ANCA-associated glomerulonephritis (AAGN). **B**–**K** The correlation between positive areas of EZH2 as well as H3K27me3 and Masson’s positive area, serum creatinine, blood urea nitrogen (BUN), urine protein, and estimated glomerular filtration rate (eGFR) in all AAGN patients (*n* = 9). Data were expressed as means ± SEM. *P < 0.05; **P < 0.01; ***P < 0.001; ****P < 0.0001. N.S., statistically not significant, with the comparisons labeled. All scale bars = 50 μm.

ANCA-associated vasculitis is a rare but severe group of diseases characterized by inflammation and necrosis of blood vessels. The majority of patients with AAV have renal involvement and are asymptomatic until advanced renal failure occurs. The clinical course of AAGN is characterized by acute and quiescent phases, and it is not uncommon to find active and chronic lesions in the same biopsy [36]. Renal biopsy is the current gold standard for establishing a diagnosis of AAGN, and the renal pathology of patients with AAGN may present cellular crescents, fibro-cellular crescents, and fibrous crescents simultaneously [37]. We enrolled 9 patients who were diagnosed with AAGN by renal biopsy and 9 patients who underwent tumor nephrectomies without diabetes or chronic renal disease to assess the expression levels of EZH2 and H3K27me3 via immunohistochemistry (IHC) staining. As shown in Fig. 1A, the kidney of patient with AAGN presented tubular atrophy and interstitial fibrosis by Masson’s trichrome and PAS staining, and EZH2 as well as H3K27me3 were highly expressed in the kidney of patients with AAGN compared with normal kidney cortex tissue, and localized specifically in tubular epithelial cells via IHC staining. Further correlation analysis (Fig. 1B–K) revealed that EZH2 and H3K27me3 expressions were correlated positively with Masson’s trichrome-positive area (EZH2: *r* = 0.790, *P* = 0.011; H3K27me3: *r* = 0.825, *P* = 0.006), serum creatinine (EZH2: *r* = 0.798, *P* = 0.010; H3K27me3: *r* = 0.841, *P* = 0.005), BUN (EZH2: *r* = 0.743, *P* = 0.022; H3K27me3: *r* = 0.833, *P* = 0.005), urine protein (EZH2: *r* = 0.922, *P* < 0.001; H3K27me3: *r* = 0.752, *P* = 0.019), and negatively with eGFR (EZH2: *r* = −0.809, *P* = 0.008; H3K27me3: *r* = −0.895, *P* = 0.001). Table S1 showed the clinical characteristics of the enrolled patients. There was no significant difference between two groups except serum creatinine, BUN, eGFR, hemoglobin, and albumin. These data suggested that with the deterioration of the renal function in AAGN patients, the expression levels of EZH2 and H3K27me3 increased.

### EZH2 conditional knockout or pharmacological depletion protects against renal dysfunction, suppresses renal fibrosis and loss of TEC transporters in I/R or FA induced AKI-to-CKD transition mice models

**Fig. 2:**
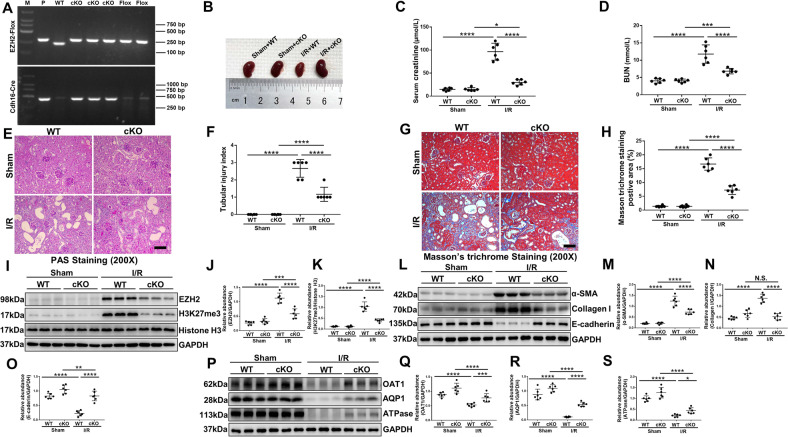
EZH2 conditional knockout protects against renal dysfunction, suppresses renal fibrosis and loss of TEC transporters in I/R induced AKI-to-CKD transition mouse model. The mouse model of AKI-to-CKD transition induced by I/R was established by clipping the bilateral renal arteries of mice for 30 min, and the animals were observed and fed for 4 weeks after operation. **A** Generation of conditional knockout mice in which EZH2 was specifically ablated in tubular epithelial cells by using Cre-LoxP recombination system. Genotyping was confirmed by tail preparation and PCR at 2 weeks of age. **B** Photograph showed the size, color, and texture of kidney in each group. **C** Serum creatinine of the mice in different groups. **D** Blood urea nitrogen (BUN) of the mice in different groups. **E** Photomicrographs showed the PAS staining of the kidneys. **F** Morphologic change of tubular injury was scored on the basis of PAS staining described in the Method section. **G** Photomicrographs showed the Masson’s trichrome staining of the kidneys. **H** The graph showed the positive areas (blue) of Masson’s trichrome staining. **I** Kidney tissue lysates from I/R mice were subjected to immunoblotting analysis with specific antibodies against EZH2, H3K27me3, Histone H3, and GAPDH. **J**, **K** Expression levels of EZH2 and H3K27me3 in different groups were quantified by densitometry and normalized with GAPDH and Histone H3 respectively. **L** Kidney tissue lysates from I/R mice were subjected to immunoblotting analysis with specific antibodies against α-SMA, Collagen I, E-cadherin, and GAPDH. **M**–**O** Expression levels of α-SMA, Collagen I, E-cadherin in different groups were quantified by densitometry and normalized with GAPDH. **P** Kidney tissue lysates from I/R mice were subjected to immunoblotting analysis with specific antibodies against OAT1, AQP1, ATPase, and GAPDH. **Q**–**S** Expression levels of OAT1, AQP1, and ATPase in different groups were quantified by densitometry and normalized with GAPDH. Data were expressed as means ± SEM. *P < 0.05; **P < 0.01; ***P < 0.001; ****P < 0.0001. N.S., statistically not significant, with the comparisons labeled. All scale bars = 50 μm.

**Fig. 3:**
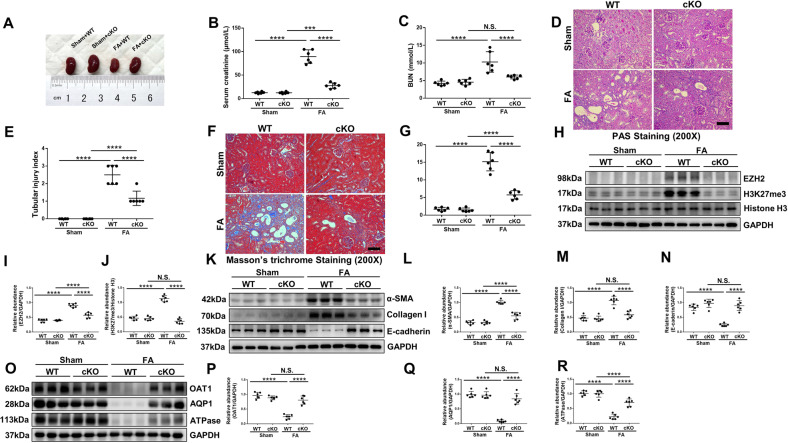
EZH2 conditional knockout protects against renal dysfunction, suppresses renal fibrosis and loss of TEC transporters in FA induced AKI-to-CKD transition mouse model. The mice in FA model were injected with a single dose of folic acid (250 mg/kg, dissolved in 300 mM NaHCO_3_, i.p.), while the control group was injected with an identical voluminal vehicle (300 mM NaHCO_3_, i.p.) for 4 weeks. **A** Photograph showed the size, color, and texture of kidney in each group. **B** Serum creatinine of the mice in different groups. **C** Blood urea nitrogen (BUN) of the mice in different groups. **D** Photomicrographs showed the PAS staining of the kidneys. **E** Morphologic change of tubular injury was scored on the basis of PAS staining described in the Method section. **F** Photomicrographs showed the Masson’s trichrome staining of the kidneys. **G** The graph showed the positive areas (blue) of Masson’s trichrome staining. **H** Kidney tissue lysates from FA mice were subjected to immunoblotting analysis with specific antibodies against EZH2, H3K27me3, Histone H3 and GAPDH. **I**, **J** Expression levels of EZH2 and H3K27me3 in different groups were quantified by densitometry and normalized with GAPDH and Histone H3 respectively. **K** Kidney tissue lysates from FA mice were subjected to immunoblotting analysis with specific antibodies against α-SMA, Collagen I, E-cadherin, and GAPDH. **L**–**N** Expression levels of α-SMA, Collagen I, E-cadherin in different groups were quantified by densitometry and normalized with GAPDH. (**O**) Kidney tissue lysates from FA mice were subjected to immunoblotting analysis with specific antibodies against OAT1, AQP1, ATPase and GAPDH. **P**–**R** Expression levels of OAT1, AQP1, and ATPase in different groups were quantified by densitometry and normalized with GAPDH. Data were expressed as means ± SEM. *P < 0.05; **P < 0.01; ***P < 0.001; ****P < 0.0001. N.S., statistically not significant, with the comparisons labeled. All scale bars = 50 μm.

To further demonstrate the role of EZH2 in AKI-to-CKD transition, we constructed EZH2 conditional knockout mice (Fig. 2A) and established two models of AKI-to-CKD transition induced by I/R or FA injury. EZH2 conditional knockout mice were generated by breeding our EZH2^fl/fl^ mice with Cdh16-Cre mice to obtain Cdh16-Cre^+^: EZH2^fl/fl^ mice (EZH2-cKO) and Cdh16-Cre^−^: EZH2^fl/fl^ mice (EZH2-WT). Our data showed that EZH2-cKO or inhibition of EZH2 with 3-DZNep significantly alleviated kidney damage and reduced serum creatinine and BUN in I/R or FA models (Figs. 2B–D, 3A–C, S1A, B, S2A–C). In addition, we examined the effect of EZH2 on renal pathological changes in I/R or FA models. I/R or FA injury mice showed severe tubular interstitial damage, such as tubular dilatation and tubular atrophy, EZH2-cKO or 3-DZNep administration mitigated tubular interstitial damage and largely preserved renal structure (Figs. 2E, F, 3D, E, S1C, D, S2D, E). As shown by Masson’s trichrome staining, I/R or FA injury mice had more positive areas of interstitial fibrosis than sham group, and EZH2-cKO or administration of 3-DZNeP reduced the increase of positive areas of interstitial fibrosis in I/R or FA injury mice (Figs. 2G, H, 3F, G, S1E, F, S2F, G). We examined the expression levels of EZH2 and H3K27me3 to confirm the knockout condition of EHZ2 and the effects of 3-DZNeP (Figs. 2I–K, 3H–J, S3A, S4A–C). Basal expression levels of EZH2 and H3K27me3 were detected in sham kidneys, and they were significantly increased due to I/R or FA injury; EZH2-cKO or 3-DZNeP administration reduced EZH2 and H3K27me3 expression levels.

In order to investigate the effects of EZH2 on myofibroblast activation and extracellular matrix precipitation after I/R or FA injury, the expressions of α-SMA and collagen I were detected by immunoblot analysis. Basal levels of α-SMA and collagen I were observed in the sham group and their expression levels were significantly upregulated after I/R or FA injury; EZH2-cKO or 3-DZNeP administration suppressed α-SMA and collagen I expressions, and the immunofluorescent result which revealed that EZH2 was expressed in both renal tubules and myofibroblasts as evidenced by its high expression in renal tubules and co-location with α-SMA in renal interstitial; In addition, I/R or FA injury reduced the expression of E-cadherin while EZH2-cKO or 3-DZNeP administration restored the expression of E-cadherin in the damaged kidneys (Figs. 2L–O, 3K–N, S3B–F, S4D–G). Taken together, these results suggested that EZH2 was involved in the development of renal interstitial fibrosis.

Fibrotic injury-induced EMT program in renal tubular epithelial cells (TECs) will lead to deregulated transporter activities [8]. Previous studies demonstrated that decreased expression of organic anion transporter 1 (OAT1) was linked to accelerated oxidative stress and inflammation, and loss of sodium/potassium-transporting ATPase subunit alpha-1 (ATPase) as well as aquaporin 1 (AQP1) in TECs are associated with renal fibrosis [38]. Immunoblot analysis results revealed that I/R or FA injury resulted in the depletion in several TEC transporters such as OAT1, ATPase, and AQP1, while EZH2-cKO or 3-DZNeP administration restored their expressions (Figs. 2P–S, 3O–R, S3G–J, S4H–K). These data supported that EZH2 promoted the EMT program induced loss of TEC transporters, and blockade of EZH2 prevented it.

### EZH2 conditional knockout or pharmacological depletion upregulates the expression of PTEN, thus blocking the activation of EGFR/ERK1/2/STAT3 signaling pathway in I/R or FA induced AKI-to-CKD transition mice models

**Fig. 4:**
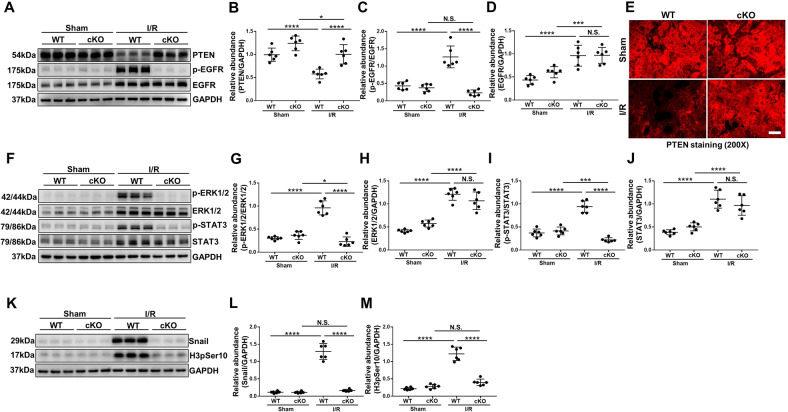
EZH2 conditional knockout upregulates the expression of PTEN, thus blocking the activation of EGFR/ERK1/2/STAT3 signaling pathway in I/R induced AKI-to-CKD transition mouse model. **A** Kidney tissue lysates from I/R mice were prepared and subjected to immunoblotting analysis with antibodies against PTEN, p-EGFR, EGFR, and GAPDH. **B**–**D** Expression levels of PTEN, p-EGFR, EGFR in different groups were quantified by densitometry and normalized with GAPDH and EGFR respectively. **E** Photomicrographs showed the immunofluorescent staining of PTEN in different groups. **F** Kidney tissue lysates from I/R mice were prepared and subjected to immunoblotting analysis with antibodies against p-ERK1/2, ERK1/2, p-STAT3, STAT3, and GAPDH. **G**–**J** Expression levels of p-ERK1/2, ERK1/2, p-STAT3, STAT3 in different groups were quantified by densitometry and normalized with GAPDH, ERK1/2, and STAT3, respectively. **K** Kidney tissue lysates from I/R mice were prepared and subjected to immunoblotting analysis with antibodies against Snail, H3pSer10, and GAPDH. **L**, **M** Expression levels of Snail and H3pSer10 in different groups were quantified by densitometry and normalized with GAPDH. Data were expressed as means ± SEM. *P < 0.05; **P < 0.01; ***P < 0.001; ****P < 0.0001. N.S., statistically not significant, with the comparisons labeled. All scale bars = 50 μm.

PTEN has been reported to be associated with the dephosphorylation of EGFR [16]. We examined the effect of EZH2 on the expression of PTEN in the I/R or FA injury kidney. In consistent with immunofluorescent results (Fig. 4E), an abundance of PTEN was detected in the sham-operated kidney, but its expression level was significantly downregulated in the I/R or FA injury kidneys; EZH2-cKO or treatment with 3-DZNeP preserved the expression of PTEN in the kidney subjected to I/R or FA injury (Fig. 4A, B, S5A, B, S6A, B, S7A, B). EGFR is cell surface receptors involved in renal fibroblast activation and proliferation [16]. The phosphorylated EGFR at Tyr1068 and the activation of its downstream signaling pathways (STAT3 and ERK1/2) were greatly increased in the kidney after I/R or FA injury, whereas EZH2-cKO or 3-DZNeP administration blocked their phosphorylation (Figs. 4A–J, S5A–I, S6A–F, S7A–F).

The activation of EGFR signaling pathway could activate EMT transcription factor Snail [39]. Recent studies have demonstrated that epithelial cell cycle arrest at G2/M phase is the functional consequence of partial EMT, and gene knockout of Snail could interrupt this process [8]. Phosphorylated histone H3 at serine 10 (H3pSer10) is a hallmark of cells arrested at the G2/M phase [40]. Therefore, we investigated the expression levels of Snail and H3pSer10 in the I/R or FA injury kidneys by immunoblot analysis. Snail and H3pSer10 were significantly increased after I/R or FA injury. EZH2-cKO or treatment with 3-DZNeP completely blocked their expressions (Figs. 4K–M, S5J–L, S6G–I, S7G–I). These data suggest that EZH2-mediated silencing of PTEN may contribute to the activation of EGFR/ERK1/2/STAT3 pathway, thus activating transcription factor Snail, and promoting epithelial cell cycle arrest at G2/M phase.

### Genetic or pharmacological depletion of EZH2 inhibits H_2_O_2_ induced partial EMT of cultured human tubular epithelial cells

**Fig. 5:**
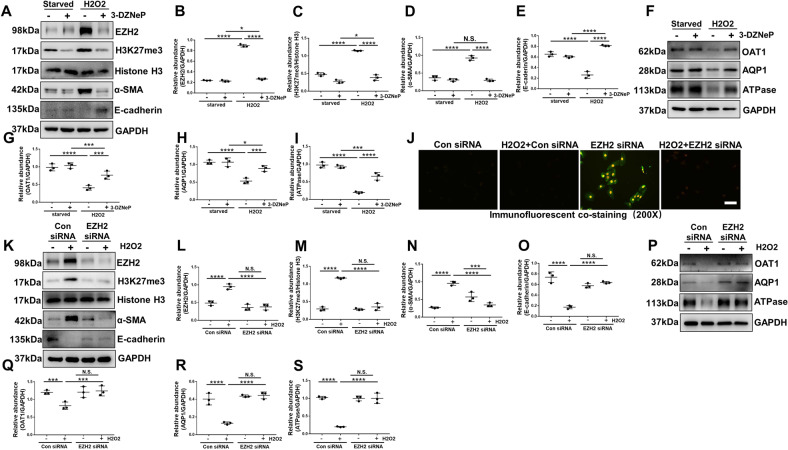
Genetic or pharmacological depletion of EZH2 inhibits H_2_O_2_ induced partial EMT of cultured human tubular epithelial cells. Starved HK2 cells were exposed to H_2_O_2_ (0.5 mM) in the presence of 3-DZNeP (5 μM) for 24 h before cell harvesting. **A** Cell lysates treated with 3-DZNeP were subjected to immunoblotting analysis with antibodies against EZH2, H3K27me3, Histone H3, α-SMA, E-cadherin, and GAPDH. **B**–**E** Expression levels of EZH2, H3K27me3, α-SMA, E-cadherin were quantified by densitometry and normalized with GAPDH and Histone H3. **F** Cell lysates treated with 3-DZNeP were subjected to immunoblotting analysis with antibodies against OAT1, AQP1, ATPase, and GAPDH. **G**–**I** Expression levels of OAT1, AQP1, ATPase were quantified by densitometry and normalized with GAPDH. **J** Photomicrographs showed the immunofluorescent co-staining of EZH2 and α-SMA in the HK2 cells of each group. HK2 cells were transfected with EZH2 siRNA and scrambled siRNA for 6 h, and then incubated with or without H_2_O_2_ (0.5 mM) for an additional 24 h before being harvested for analysis. **K** Cell lysates transfected with EZH2 siRNA were subjected to immunoblotting analysis with antibodies against EZH2, H3K27me3, Histone H3, α-SMA, E-cadherin, and GAPDH. **L**–**O** Expression levels of EZH2, H3K27me3, α-SMA, E-cadherin were quantified by densitometry and normalized with GAPDH and Histone H3. **P** Cell lysates transfected with EZH2 siRNA were subjected to immunoblotting analysis with antibodies against OAT1, AQP1, ATPase, and GAPDH. **Q**–**S** Expression levels of OAT1, AQP1, ATPase were quantified by densitometry and normalized with GAPDH. Data were expressed as means ± SEM. *P < 0.05; **P < 0.01; ***P < 0.001; ****P < 0.0001. N.S., statistically not significant, with the comparisons labeled. All scale bars = 50 μm.

In vitro, to further understand the role of EZH2 in AKI-to-CKD transition, we examined the effect of 3-DZNeP or siRNA specifically targeting EZH2 on H_2_O_2_-induced EMT in HK2 cells. As shown in Fig. 5A–C, K–M, H_2_O_2_ increased the expression of EZH2 and H3K27me3, and blockade of EZH2 with 3-DZNeP or siRNA decreased the expression of EZH2 and H3K27me3. H_2_O_2_ stimulation significantly increased the expression of α-SMA and suppressed E-cadherin expression levels, blockade of EZH2 inhibited upregulation of α-SMA and downregulation of E-cadherin (Fig. 5A, D, E, K, N, O). Immunofluorescent staining indicated that EZH2 and α-SMA expressions were markedly increased in renal tubular cells under the stimulation of H_2_O_2_, 3-DZNeP treatment significantly reduced their expressions (Fig. 5J). Collectively, these data suggested that EZH2 activation was critically involved in the EMT of HK2 cells. OAT1, ATPase, and AQP1 are expressed in normal TECs [8]. H_2_O_2_ significantly decreased the expressions of OAT1, ATPase, and AQP1, and inhibition of EZH2 blocked these downregulations (Fig. 5F–I, P–S).

### EZH2 binds to the PTEN promoter and regulates its transcription, thus increasing the activation of EGFR/ERK1/2/STAT3 signaling pathway in cultured human tubular epithelial cells

**Fig. 6:**
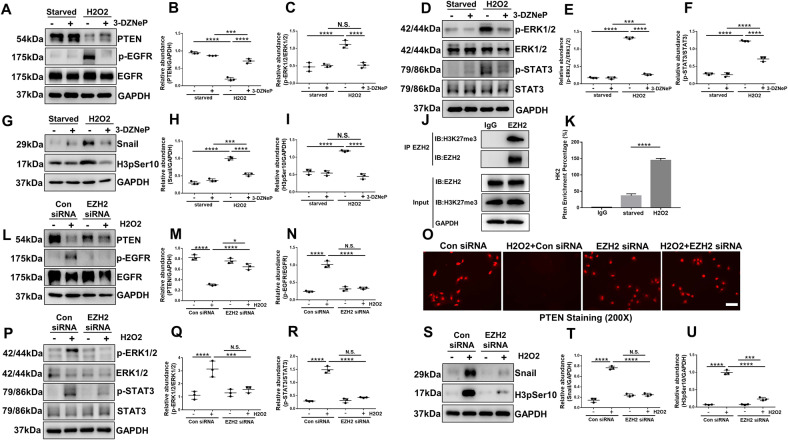
EZH2 binds to the PTEN promoter and regulates its transcription, thus increasing the activation of EGFR/ERK1/2/STAT3 signaling pathway in cultured human tubular epithelial cells. **A** Cell lysates treated with 3-DZNeP were subjected to immunoblotting analysis with antibodies against PTEN, p-EGFR, EGFR, and GAPDH. **B**, **C** Expression levels of PTEN and p-EGFR were quantified by densitometry and normalized with GAPDH and EGFR, respectively. **D** Cell lysates treated with 3-DZNeP were subjected to immunoblotting analysis with antibodies against p-ERK1/2, ERK1/2, p-STAT3, STAT3, and GAPDH. **E**, **F** Expression levels of p-ERK1/2 and p-STAT3 were quantified by densitometry and normalized with ERK1/2 and STAT3, respectively. **G** Cell lysates treated with 3-DZNeP were subjected to immunoblotting analysis with antibodies against Snail, H3pSer10, and GAPDH. **H**, **I** Expression levels of Snail and H3pSer10 were quantified by densitometry and normalized with GAPDH. **J** 0.5 mM H_2_O_2_-treated HK2 cell lysates were subjected to immunoprecipitation with IgG or EZH2 antibody, followed by EZH2 and H3K27me3 immunoblotting. **K** Content of PTEN promoter enriched by IgG or H3K27me3 antibody in HK2 cells treated with or without 0.5 mM H_2_O_2_ by CUT & TAG analysis. **L** Cell lysates transfected with EZH2 siRNA were subjected to immunoblotting analysis with antibodies against PTEN, p-EGFR, EGFR, and GAPDH. **M**, **N** Expression levels of PTEN and p-EGFR were quantified by densitometry and normalized with GAPDH and EGFR respectively. **O** Photomicrographs showed the immunofluorescent staining of PTEN in the HK2 of each group. **P** Cell lysates transfected with EZH2 siRNA were subjected to immunoblotting analysis with antibodies against p-ERK1/2, ERK1/2, p-STAT3, STAT3, and GAPDH. **Q**, **R** Expression levels of p-ERK1/2 and p-STAT3 were quantified by densitometry and normalized with ERK1/2 and STAT3, respectively. **S** Cell lysates transfected with EZH2 siRNA were subjected to immunoblotting analysis with antibodies against Snail, H3pSer10, and GAPDH. **T**, **U** Expression levels of Snail and H3pSer10 were quantified by densitometry and normalized with GAPDH. Data were expressed as means ± SEM. *P < 0.05; **P < 0.01; ***P < 0.001; ****P < 0.0001. N.S., statistically not significant, with the comparisons labeled. All scale bars = 50 μm.

In vitro, H_2_O_2_ stimulation led to the activation of EGFR/ERK1/2/STAT3 signaling pathway in HK2 cells, while blockade of EZH2 with 3-DZNeP or siRNA inhibited these changes (Fig. 6A, C–F, L, N, P–R). Immunofluorescent staining indicated that PTEN expression was markedly decreased in renal tubular cells under the stimulation of H_2_O_2_, while blockade EZH2 with siRNA increased its expression (Fig. 6O). Immunoblot analysis results were consistent with it (Fig. 6A, B, L, M). In order to further explore the regulatory role of EZH2 on PTEN, CO-IP, and CUT & Tag assay were performed. CO-IP assay indicated that EZH2 interacts with H3K27me3 in HK2 cells (Fig. 6J). CUT & Tag assay revealed that H3K27me3 was enriched in the PTEN promoter region in HK2 cells treated with H_2_O_2_ (Fig. 6K). These results suggested that EZH2 binds to the PTEN promoter to regulate its transcription, thus regulating the activation of EGFR/ERK1/2/STAT3 signaling pathway. As shown in Fig. 6G–I, S–U, H_2_O_2_ increased the expression of Snail and H3pSer10 indicating that HK2 cells were arrested in G2/M stage, and blockade of EZH2 with 3-DZNeP or siRNA interrupted this process.

### Genetic or pharmacological depletion of EZH2 inhibits M2 macrophage polarization through STAT6 and PI3K/AKT pathways in Raw264.7 cells

Inflammation initiates as a protective response to injury, however, when it persists, it may contribute to fibrotic progression [8]. Apart from inflammatory cell infiltration, damaged TECs can be transformed into a secretory phenotype and act as proinflammatory mediators [7]. An earlier study [41] that explored the interaction between activated renal tubule cells expressing myofibroblast markers and peritubular cells (particularly inflammatory cells), suggesting a relation between EMT and recruitment of inflammatory cells. Grande et al. [42] and Lovisa et al. [8, 43] further observed decreased secretion of cytokines and chemokines in tubular cells, and evidently reduction in the presence of F4/80 macrophages and the level of M2 macrophages (which are thought to be pro-fibrogenic during tissue repair) after EMT inhibition by deletion of Snail. Taken together, these data suggest that EMT promotes the related secretion of TECs and increases subsequent inflammation. Therefore, in order to explore the regulatory effects of various proinflammatory factors secreted by TECs underwent EMT on macrophages, we cultured RAW264.7 cells with or without 10% (vol/vol) pre-collected cell culture media from HK2 cells treated with H_2_O_2_.

**Fig. 7:**
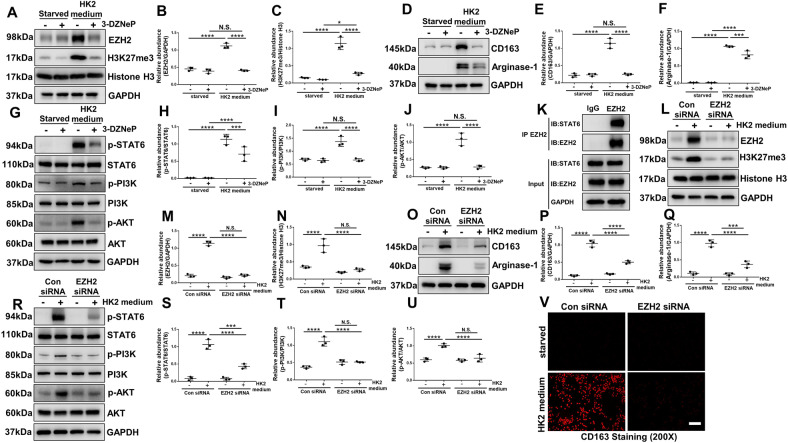
Genetic or pharmacological depletion of EZH2 inhibits M2 macrophage polarization through STAT6 and PI3K/AKT pathways in Raw264.7 cells. Starved Raw264.7 cells were cultured with or without 10% (vol/vol) pre-collected cell culture media from HK2 cells treated with 0.5 mM H_2_O_2_ in the presence of 3-DZNeP (5 μM) for 24 h before cell harvesting. **A** Cell lysates treated with 3-DZNeP were subjected to immunoblotting analysis with antibodies against EZH2, H3K27me3, Histone H3, and GAPDH. **B**, **C** Expression levels of EZH2 and H3K27me3 were quantified by densitometry and normalized with GAPDH and Histone H3. **D** Cell lysates treated with 3-DZNeP were subjected to immunoblotting analysis with antibodies against CD163, Arginase-1, and GAPDH. **E**, **F** Expression levels of CD163 and Arginase-1 were quantified by densitometry and normalized with GAPDH. **G** Cell lysates treated with 3-DZNeP were subjected to immunoblotting analysis with antibodies against p-STAT6, STAT6, p-PI3K, PI3K, p-AKT, AKT, and GAPDH. **H**–**J** Expression levels of p-STAT6, p-PI3K, and p-AKT were quantified by densitometry and normalized with STAT6, PI3K, and AKT. **K** Raw264.7 cell lysates cultured with 10% (vol/vol) pre-collected cell culture media from HK2 cells treated with 0.5 mM H_2_O_2_ were subjected to immunoprecipitation with IgG or EZH2 antibody, followed by EZH2 and STAT6 immunoblotting. RAW264.7 cells were transfected with EZH2 siRNA and scrambled siRNA for 6 h, and then incubated with or without 10% (vol/vol) pre-collected cell culture media from HK2 cells treated with 0.5 mM H_2_O_2_ for an additional 24 h before being harvested for analysis. **L** Cell lysates transfected with EZH2 siRNA were subjected to immunoblotting analysis with antibodies against EZH2, H3K27me3, Histone H3, and GAPDH. **M**, **N** Expression levels of EZH2 and H3K27me3 were quantified by densitometry and normalized with GAPDH and Histone H3. **O** Cell lysates transfected with EZH2 siRNA were subjected to immunoblotting analysis with antibodies against CD163, Arginase-1, and GAPDH. (**P**, **Q**) Expression levels of CD163 and Arginase-1 were quantified by densitometry and normalized with GAPDH. **R** Cell lysates transfected with EZH2 siRNA were subjected to immunoblotting analysis with antibodies against p-STAT6, STAT6, p-PI3K, PI3K, p-AKT, AKT, and GAPDH. **S**–**U** Expression levels of p-STAT6, p-PI3K, and p-AKT were quantified by densitometry and normalized with STAT6, PI3K, and AKT. **V** Photomicrographs showed the immunofluorescent staining of CD163 in the RAW264.7 of each group. Data were expressed as means ± SEM. *P < 0.05; **P < 0.01; ***P < 0.001; ****P < 0.0001. N.S., statistically not significant, with the comparisons labeled. All scale bars = 50 μm.

Immunoblot analysis revealed that co-cultured with 10% (vol/vol) pre-collected cell culture media from HK2 cells treated with H_2_O_2_ increased the expression of EZH2 and its substrate H3K27me3, and administration of 3-DZNeP or siRNA could effectively inhibit their expressions (Fig. 7A–C, L–N). Co-cultured with HK2 medium could induce M2 macrophage polarization, which was manifested as increased expressions of M2 phenotype macrophage-specific markers, Arginase-1 and CD163 [44], while deletion of EZHZ reduced their expressions (Fig. 7D–F, O–Q). Immunofluorescent analysis showed that CD163-positive macrophages increased after co-cultured with HK2 medium (Fig. 7V). Previous studies have demonstrated that STAT6 and PI3K/AKT pathways regulated the polarization of macrophages toward M2 phenotype [28]. In our research, we found that co-cultured with HK2 medium led to the activation of STAT6 and PI3K/AKT pathways in RAW264.7 cells, while blockade of EZH2 with 3-DZNeP or siRNA inhibited these changes (Fig. 7G–J, R–U). In order to further explore the regulatory role of EZH2 on STAT6, CO-IP assay were performed. CO-IP assay indicated that EZH2 interacted with STAT6 in RAW264.7 cells co-cultured with HK2 medium (Fig. 7K). These results further verified the regulatory role of EZH2 on STAT6 signaling pathway.

### EZH2 conditional knockout or pharmacological depletion inhibits M2 macrophage polarization through STAT6 and PI3K/AKT pathways in I/R or FA induced AKI-to-CKD transition mice models

**Fig. 8:**
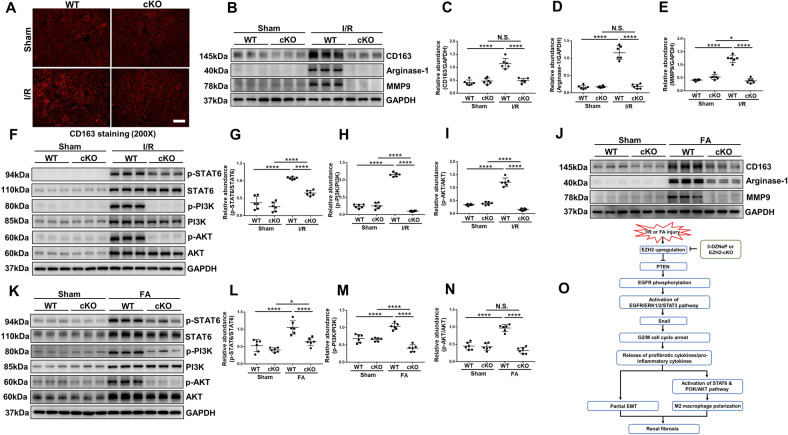
EZH2 conditional knockout inhibits M2 macrophage polarization through STAT6 and PI3K/AKT pathways in I/R or FA induced AKI-to-CKD transition mice models. **A** Photomicrographs showed the immunofluorescent staining of CD163 in different groups. **B** Kidney tissue lysates from I/R mice were prepared and subjected to immunoblotting analysis with antibodies against CD163, Arginase-1, MMP9, and GAPDH. **C**–**E** Expression levels of CD163, Arginase-1, MMP9 in different groups were quantified by densitometry and normalized with GAPDH. **F** Kidney tissue lysates from I/R mice were prepared and subjected to immunoblotting analysis with antibodies against p-STAT6, STAT6, p-PI3K, PI3K, p-AKT, AKT, and GAPDH. **G**–**I** Expression levels of p-STAT6, p-PI3K, and p-AKT were quantified by densitometry and normalized with STAT6, PI3K, and AKT. **J** Kidney tissue lysates from FA mice were prepared and subjected to immunoblotting analysis with antibodies against CD163, Arginase-1, MMP9, and GAPDH. **K** Kidney tissue lysates from FA mice were prepared and subjected to immunoblotting analysis with antibodies against p-STAT6, STAT6, p-PI3K, PI3K, p-AKT, AKT, and GAPDH. **L**–**N** Expression levels of p-STAT6, p-PI3K, and p-AKT were quantified by densitometry and normalized with STAT6, PI3K, and AKT. **O** The role and mechanisms of EZH2 in AKI-to-CKD transition. Data were expressed as means ± SEM. *P < 0.05; **P < 0.01; ***P < 0.001; ****P < 0.0001. N.S., statistically not significant, with the comparisons labeled. All scale bars = 50 μm.

Macrophage infiltration is considered to be an important driver of AKI progression and there is increasing evidence that activation of M2 macrophages is involved in the progression from AKI to CKD [12, 13]. In this research, phenotypes, and functions of M2 macrophage, including the expressions of surface markers (CD163 and Arginase-1) [45] and secretion protein of M2 macrophage such as metalloproteinase 9 (MMP-9) were assayed [46]. Immunofluorescent analysis (Fig. 8A) revealed that CD163-positive macrophages increased in fibrotic kidney induced by I/R injury which was in consistence with the immunoblot analysis results (increased expressions of Arginase-1, CD163, and MMP-9), while EZH2-cKO or administration of 3-DZNeP markedly inhibited these changes (Figs. 8B–E, J, S8A–D, S9A–D). These results indicated that M2 polarization macrophage participated in AKI to CKD progression, and EZH2 played a regulatory role in this process. Furthermore, I/R or FA injury increased the phosphorylation of STAT6, PI3K, and AKT, while deletion of EZH2 blocked their phosphorylation (Figs. 8F–I, K–N, S8E–K, S9E–K). These results suggested that EZH2 inhibition reduced M2 polarization macrophage via suppressing the activation of STAT6 and PI3K/AKT pathway in AKI-to-CKD transition.

Finally, we verified the safety and adverse reaction of PCI-34051 treatment in FA model. H&E staining showed that there was no obvious pathological damage in heart, liver, spleen, lung, and intestine after PCI-34051 treatment (Figure S10), which paved the way for the later clinical trials.

## Discussion

AKI is a common clinical syndrome, and while the majority of patients may recover from AKI, 29.4% of patients with AKI progress to CKD stage 3 or higher within the subsequent one year, resulting in a substantial significant financial burden on public health [47]. Previous studies of our research group have revealed that blockade of EZH2 protects against AKI [33, 48] and EZH2 could sustainably promote the progression of renal fibrosis in CKD mice (renal injury induced by hyperuricemia and UUO) [16, 49, 50]. However, EZH2 was only inhibited by the specific inhibitor in the previous in vivo studies and the mechanism of the transition from AKI to CKD is unclear. In this research, we found that EZH2 and H3K27me3 highly upregulated in kidneys from patients with ANCA-associated glomerulonephritis, and expressed positively with fibrotic lesion and negatively with renal function. Given that the mechanism of the AKI-CKD transition remains elusive, investigation of different AKI-CKD models may provide a comprehensive insight into the mechanism of the AKI-CKD transition. Therefore, we used EZH2 specific inhibitor (3-DZNeP) and EZH2 conditional knockout mice to explore the role and mechanism of EZH2 in renal fibrosis after acute kidney injury in IR and FA mice models (two common models used for studying AKI-CKD transition) [51]. Further investigation in mechanism revealed that blockade of EZH2 was effective in suppressing EMT via binding to the PTEN promoter and regulating its transcription, thus reducing the activation of EGFR/ERK1/2/STAT3 signaling pathway. Furthermore, blockade of EZH2 inhibited M2 macrophage polarization via reducing the phosphorylation of STAT6, PI3K, and AKT, ultimately leading to attenuated fibrosis. These data provided the evidence supporting EZH2 as a promising therapeutic target of AKI-to-CKD transition.

Kidneys encounter different types of AKI that can damage their TECs, then damaged TECs release growth factors, cytokines, chemokines, and MMPs that mediates the initiation of the EMT program and the initial influx of macrophages [52]. The functional consequence of EMT program during fibrotic injury is an arrest in the G2/M phase and decreased expression of several transporters in TECs [8]. Previous studies demonstrate that decreased expression of OAT1 is linked to accelerated oxidative stress and inflammation [52], and loss of ATPase and AQP1 in TECs are associated with renal fibrosis [8]. In our research, we demonstrated that blockade of EZH2 inhibited TECs arrested at the G2/M phase and prevented the loss of transporters, leading to protection of TEC integrity, which was helped in restoring proliferation and de-differentiation-associated repair and regeneration, as well as attenuating myofibroblast accumulation and fibrosis [8].

EGFR as a growth factor receptor is critically involved in renal fibrogenesis [53, 54], and inhibition of EGFR have been proved to account for the anti-fibrotic effect of 3-DZNeP observed in UUO-induced renal fibrosis [16]. However, it remains unclear how EZH2 is coupled to EGFR and regulates its phosphorylation and activation during AKI to CKD progression. PTEN is a protein tyrosine phosphatase that can dephosphorylate EGFR [16]. In this research, we found that upregulation of PTEN and phosphorylation inhibition of EGFR via deletion of EZH2 could reduce subsequently renal fibrosis after I/R or FA injury, and vitro experiments confirmed that EZH2 binding to the PTEN promoter to regulate its transcription in HK2 cells under H_2_O_2_ injury. Therefore, we hypothesized that after AKI injury, EZH2 regulates PTEN transcription in TECs, thereby affecting its expression, and the subsequently phosphorylation of EGFR to regulate renal fibrosis. In addition, blockade of EZH2 also affects the activation of downstream signaling pathways of EGFR, including STAT3 and ERK1/2. On one hand, the reduction of STAT3 and ERK1/2 phosphorylation might be secondary to its suppression on their upstream receptor tyrosine kinase; on the other hand, previous research has proved that EZH2 could regulate the activation of STAT3 by directly inducing STAT3 methylation [55].

Kidney fibrosis is a multi-factorial and multi-cellular disease. All cell types present in the kidney including epithelial cells, endothelial cells, mesangial cells, as well as immune cells contribute to the progression of chronic kidney diseases after AKI [8]. AKI attack damages TECs, and subsequently induces the EMT program as well as the transformation of TECs into a pathological secretome [8, 56, 57], leading to the secretion of various proinflammatory cytokines, including IL-1β, interleukin-6 (IL-6), tumor necrosis factor-α (TNF-α) [58], IL-4, and IL-10 [59]. The local cytokine milieu can orientate macrophage polarization. M2 macrophages play a dominant role in fibrotic kidney diseases, including Alport syndrome, Orellanus syndrome, Balkan nephropathy, Chinese herb nephropathy, and chronic allograft failure [60]. Previous studies have proved that M2 macrophages can mediate renal fibrosis by secreting anti-inflammatory cytokines IL-10 and TGF-β1 [60–62], as well as vascular endothelial growth factor (VEGF) promoting angiogenesis [63], or the transition of monocyte/macrophage to myofibroblast [64]. TGF-β1 secreted by M2 macrophages can further induce the development of EMT and result in a vicious cycle of damage and host response, leading to chronic fibrosis [8]. M2 macrophages, which are anti-inflammatory, can be polarized by IL-4 and IL-13 via activating STAT6 through the IL-4 receptor alpha [65]. Activation of STAT6 after binding to the receptor involves multiple steps. First, the association of STAT6 with tyrosine phosphorylated regions of cytokine receptors occurs through its SH2 domain. Then, STAT6 is phosphorylated on Y641 [66] and dimerized [67]. Subsequently, phosphorylated STAT6 translocates to the nucleus, binding to specific consensus sequences and facilitating downstream gene transcription [68, 69]. In our research, we found that blockade of EZH2 reduced M2 macrophage polarization and STAT6 phosphorylation in I/R or FA models. We further confirmed the interaction between EZH2 and STAT6 through Co-IP assay. It has been demonstrated that STAT6 activity is regulated by arginine methylation of Arg27 [70]. In the absence of methylation, STAT6 phosphorylation is significantly decreased in response to IL-4, and there is no nuclear translocation or DNA-binding activity, indicating that methylation of STAT6 is critical for its function. Therefore, we speculate that EZH2, as a histone methyltransferase, is involved in regulating the activation of STAT6 through H3K27me3, and the specific mechanism remains to be further verified.

In addition, the activation of PI3K/AKT signaling pathway also results in an M2 phenotype, thus increasing TGF-β1 upregulation as well as fibroblast activation [71]. In our research, EZH2 inhibition suppressed the activation of PI3K/AKT, thus reducing M2 macrophage polarization. PTEN is a typical upstream suppressor of PI3K/AKT pathway [72]. Our research revealed that EZH2 could directly bind to the promoter of PTEN and reduce its expression, thus PTEN inhibition further promoted the activation of PI3k/AKT pathway. In addition, PI3K Interacting Protein 1 (PIK3IP1), which binds to the p110 catalytic subunit of PI3K and can subsequently reduce its activity [73], is also a direct substrate for EZH2 [74]. EZH2 inhibition upregulates PIK3IP1 expression, thereby inhibiting PI3K/AKT signaling.

Increasing evidence has indicated that EZH2 plays an essential role in mediating tissue fibrosis [16, 75, 76]. In the current study, we provided strong evidence that EZH2 is critically involved in the AKI to CKD progression. Up to now, several EZH2 inhibitors have been in the treatment of tumors in clinical trials [77, 78]. Our study further validated the anti-fibrosis efficacy of EZH2 inhibitors in animal models of renal disease, and provided theoretical support for the use of EZH2 inhibitors in clinical trials.

In conclusion, we demonstrated the important role of EZH2 in mediating the progression from AKI to CKD by promoting EMT via binding to the PTEN promoter and regulating its transcription, thus promoting the activation of EGFR/ERK1/2/STAT3 signaling pathway, and activating the polarization of M2 macrophages via STAT6 and PI3K/AKT pathway, eventually led to kidney fibrosis. Targeted inhibition of EZH2, therefore, could be a novel therapy for preventing the progression from AKI to CKD.

## Supplementary information

Supplementary Materials

Reproducibility checklist

Original Data File

## Data Availability

The experimental data sets generated and/or analyzed during the current study are available from the corresponding author upon reasonable request. No applicable resources were generated during the current study.
