# Supplementary material for: Enhancer of zeste homolog 2 promotes renal fibrosis after acute kidney injury by inducing epithelial-mesenchymal transition and activation of M2 macrophage polarization
Source: Cell Death Dis. 2023 Apr 7;14(4):253. doi: 10.1038/s41419-023-05782-4 (PMC10081989; doi:10.1038/s41419-023-05782-4)
Supplement: Supplementary file 1 — Supplementary Materials [file 41419_2023_5782_MOESM1_ESM.docx]

Supplementary Materials for

**Enhancer of zeste homolog 2 promotes renal fibrosis after acute kidney injury by induing epithelial-mesenchymal transition and activation of M2 macrophage polarization**

Xun Zhou^1*^, Hui Chen^1*^, Yan Hu^1*^, Xiaoyan Ma^1^, Jinqing Li^1^, Yingfeng Shi^1^, Min Tao^1^, Yi Wang^1^, Qin Zhong^1^, Danying Yan^1^, Shougang Zhuang^1,2^, Na Liu^1^

**Correspondence and offprint requests to:** **Na Liu**, M.D., Ph.D., Department of Nephrology, Shanghai East Hospital, Tongji University School of Medicine, 150 Jimo road, Pudong new district, Shanghai 200120, China. **E-mail**: naliubrown@163.com.

**This PDF file includes:**

**1. Supplementary Material and Methods**

**2. Supplementary Figures**

**3. Supplementary Tables**

1. **Supplementary Material and Methods**

**Human renal biopsy samples**

Renal biopsies had been performed as part of routine clinical diagnostic investigation and collected as described in Table S1. For the detection of EZH2 and H3K27me3 expression levels, we enrolled 9 patients who were diagnosed with ANCA-associated vasculitis by renal biopsy. The samples of renal biopsies were obtained from Department of Nephrology, Shanghai East Hospital affiliated with Tongji University from January 2017 to May 2022. Control samples (n=9) were obtained from the healthy kidney tissues of individuals who underwent tumor nephrectomies without diabetes or chronic renal disease. We obtained the specimens from surgical tissues and conducted immunohistochemical staining of EZH2 and H3K27me3. The information involved laboratory examination was obtained from case system of Shanghai East Hospital. This study was approved by the Medical Ethics Committee of Shanghai East Hospital and was conducted in accordance with the Declaration of Helsinki. Written informed consent was obtained from each patient. And we have obtained the registration number from the Chinese Clinical Trial Register (ChiCTR): ChiCTR2200055136.

**Reagents and kits**

3-DZNeP was purchased from Selleckchem (Houston, TX, USA). FITC and Texas Red for immunofluorescent staining were purchased from Thermo Fisher Scientific (Waltham, MA, UK). Serum creatinine and BUN biochemical reagent kits were purchased from Nanjing Jiancheng Bioengineering Institute (Nanjing, China). EZH2 siRNA was purchased from GenePharma (Shanghai, China). Lipofectamine 3000 was purchased from Invitrogen (Grand Island, NY, USA). TGF-β1 was purchased from R&D Systems (Minneapolis, MN, USA). RIPA lysis buffer and IgG were purchased from Beyotime (Shanghai, China). Protein A/G beads were purchased from Santa Cruz Biotechnology, (Santa Cruz, CA, USA). Folic acid, secondary antibodies for Western blot, and all other chemicals were purchased from Sigma (St. Louis, MO, USA).

**Animals and treatment**

All animal work was performed at Tongji University School of Medicine (Shanghai, China). C57/black mice (provided by Shanghai Super-B&K Laboratory Animal Corp. Ltd, Shanghai, China) weighed 20-25g were maintained in a pathogen-free facility under a 12h light-dark cycle with abundant food and water supplied. Two mice models of AKI-to-CKD transition induced by ischemia-reperfusion (I/R) or folic acid (FA) were established. The I/R model was established by clipping the bilateral renal arteries of mice for 30 minutes^1^. The animals were under general anesthesia induced by intraperitoneal injection of a mixture of ketamine hydrochloride (100 mg/kg) and xylazine hydrochloride (10 mg/kg), then a midline abdominal incision was made, and bilateral renal pedicles were clipped for 30 minutes using microaneurysm clamps for severe AKI, respectively. After removal of the clamps, reperfusion of the kidneys was visually confirmed. The incision was then closed in two layers. During the ischemic period, body temperature was maintained between approximately 37°C and 38°C using a temperature-controlled heating system. The animals were observed and fed for 4 weeks after operation. The FA model was established by one intraperitoneal dose of folic acid (250 mg/kg) dissolved in 300 mM NaHCO3^2^, while the control group was injected with an identical voluminal vehicle (300 mM NaHCO3, i.p.) for four weeks. To investigate the effect of 3-DZNeP on renal fibrosis, mice in I/R or FA model were intraperitoneally injected with 3-DZNeP (1 mg/kg) in saline every day. The sham group was injected with an equal volume of saline as a control. Mice were randomly divided into four groups in each model: (1) mice injected with an equivalent amount of saline intraperitoneally and DMSO (n=6), defined as the sham group; (2) mice injected an equivalent amount of saline intraperitoneally and 3-DZNeP (n=6), defined as sham + 3-DZNeP group; (3) mice with I/R injury or injected FA intraperitoneally (n=6), defined as I/R or FA group; (4) mice with I/R injury or injected FA intraperitoneally (n=6) and injected 3-DZNeP intraperitoneally, defined as I/R or FA + 3-DZNeP group (n=6). After four weeks for I/R or FA, the animals were sacrificed by exsanguination under anesthesia with inhaled 5% isoflurane in room air and the kidney samples were collected for protein analysis and histological examination. Blood was taken for the measurement of BUN and serum creatinine. The animal protocol was reviewed and approved by the Institutional Animal Care and Use Committee at Tongji University (Shanghai, China).

**EZH2 conditional knockout mouse model**

To generate compound mice EZH2*^fl/fl^*Cdh16-Cre^+/-^(EZH2-cKO), heterozygous EZH2^+/^*^flox^* mice (Stock No:022616) purchased from The Jackson Laboratory (Bar Harbor, ME, USA), were crossed with cadherin 16-cre mice (Tg(Cdh16-cre)91Igr/J, Stock No: 012237), purchased from The Jackson Laboratory (Bar Harbor, ME, USA). Both of EZH2-cKO mice and EZH2-WT mice were clipped the bilateral renal arteries for 30 minutes to establish I/R-induced AKI-to-CKD transition model. Another part of mice was injected with a single dose of folic acid (250 mg/kg, dissolved in 300 mM NaHCO3, i.p.) for four weeks to establish FA-induced AKI-to-CKD transition model.

**Transfection of siRNA**

The EZH2 siRNA was synthesis by GenePharma (Shanghai, China). The sequences of EZH2 siRNA were listed in Table S2. Transfection of siRNA was performed according to the manufacturer’s protocol, respectively. Briefly, HK2 cells or RAW264.7 cells were seeded to 70-80% confluence in the antibiotic-free medium and grown followed by transfection with EZH2 siRNA (60 pmol) using Lipofectamine 3000 (CA, USA). In parallel, scramble siRNA (60 pmol) was used as a control for off-target changes in HK2 cells or RAW264.7 cells. 6 hours after transfection, the medium was changed to DMEM with F12 containing 0.5% FBS or DMEM with 1640 containing 0.5% FBS for starvation and then cells were incubated with or without H2O2 (0.5mM) or 10% (vol/vol) pre-collected cell culture media from HK2 cells for an additional 24 hours before being harvested for analysis. All of the in vitro experiments were repeated for at least three times.

**Co-immunoprecipitation (Co-IP)**

HK2 cells or RAW264.7 cells were collected and lysed with RIPA lysis buffer (Beyotime, Shanghai, China) and prepared for Co-IP assay. After incubation with 2µg anti-EZH2 antibody or IgG (Beyotime, Shanghai, China) as negative control for 2 hours at 4°C, 20µl protein A/G beads (Santa Cruz Biotechnology, Santa Cruz, CA, USA) were added and incubated overnight at 4°C. The next day, after centrifuged, the deposit was collected and washed with lysis buffer for three times, then incubated in 40µl 1×loading buffer and boiled at 100°C for 10 minutes for subsequent immunoblotting analysis.

**Cut & Tag**

Cut & Tag was done using the Yeasen (Shanghai, China) Cut & Tag kit (12598ES04/12/48) following the manufacturer’s protocol. Briefly, 100,000 cells were harvested, and bound to concanavalin A-coated magnetic beads and incubated in an anti-H3K27me3 antibody for 2 hours. After removing the primary antibody on a magnetic stand, a goat anti-rabbit IgG was incubated for 1 hour, followed by incubation with TAG buffer mixed with pA/G-Transposome for 1 hour. Next, the cells were incubated with activating buffer for 1 hour. Then, proteinase K and Terminate Solution were used at 55°C for 30 minutes to stop the tagmentation. DNA was then extracted with DNA selection beads. The amount of immunoprecipitated DNA for each specific antibody was quantified in triplicate by quantitative PCR. Values of retrieved DNA were related to the amount of input DNA to quantify the efficiency of the immunoprecipitation. Negative controls included the same samples incubated with rabbit IgG as the primary antibody. Primer sequences are listed in Table S2.

**Immunoblotting analysis**

Samples were all equilibrated to 20μg and run on a 4-15% SDS-PAGE gel, transferred to a nitrocellulose membrane, and blocked in 5% nonfat biological grade powdered milk dissolved in 25mM Tris HCl (pH 7.4), 137 mM NaCl, and 0.1% TWEEN20 (TBST) for 30 minutes. Blots were washed with TBST and incubated with primary antibody in 5% BSA with TBST for 1 hours or overnight. All primary antibodies were listed in Table S3. The membranes were then washed three times at 10 minutes intervals with TBST prior to addition of secondary antibody for 1 hours. Blots were developed with BeyoECL Plus Chemiluminescence Kit (Beyotime Biotechnology, Shanghai, China) according to the manufacturer’s instruction. The densitometry analysis of immunoblot results was conducted by using ImageJ software (National Institutes of Health, Bethesda, MD, USA).

**Renal function**

Blood samples were collected and centrifugated. Commercial assay kits from Nanjing Jiancheng (Nanjing, China) were used to detect the levels of serum creatinine and BUN. After the color developing agent was added, mixture was measured spectrophotometrically at different wavelengths respectively. According to the standard curve, their levels could be calculated.

**Pathological Assessment**

Formalin-fixed kidneys were embedded in paraffin and prepared in 3-μm-thick sections. For assessment of renal fibrosis, Masson’s trichrome staining was performed according to the protocol provided by the manufacture (Sigma, St. Louis, MO, USA). The collagen-positive tissue area (blue color in Masson trichrome) was quantitatively measured using Image Pro-Plus software (Media-Cybernetics, Silver Spring, MD, USA) by drawing a line around the perimeter of positive staining area, and the average ratio to each microscopic field was calculated and graphed. For general histology, sections were stained with PAS (Sigma, St. Louis, MO, USA). To assess the extent of tubular injury, morphologic damage (epithelial necrosis, luminal necrotic debris, and tubular dilation) in three sections per kidney and ten fields per section were quantified using the following scale: none=0, <30%=1, 30%-60%=2 and >60%=3.

**Hematoxylin and eosin (H&E) staining**

Mouse samples of heart, liver, spleen, lung and intestine were excised immediately, fixed in 4% paraformaldehyde (PFA), then paraffin-embedded. The paraffin sections were dewaxed in xylene and dehydrated with ethanol, then stained with hematoxylin and eosin (H&E) to assess general conditions of multiple organs after 3-DZNeP treatment. H&E-slide images were viewed with a microscope equipped with a digital camera (BX53, OLYMPUS, Shanghai, China).

**Immunohistochemical and immunofluorescent staining**

Immunohistochemical and immunofluorescent staining were performed according to procedures described in our previous studies^3^. For immunofluorescent staining of kidney, the tissue sections were rehydrated and labeled with primary antibodies, including α-SMA, EZH2, and E-cadherin etc. then exposed to Texas red-labeled or FITC green-labeled secondary antibodies. For immunofluorescent staining of HK2 or RAW264.7, cells were cultured on sterile glass cover slips in 24-well plates, washed three times with PBS, and fixed with 4% paraformaldehyde for 15 minutes. The fixed cells were then washed twice with PBS, permeabilized for 10 minutes with 0.2% Triton X-100, blocked for 1 hours in 5% BSA, and incubated with the appropriate primary antibodies overnight at 4°C. All primary antibodies are listed in Table S4. Cells were labeled for 1 hour using secondary antibodies conjugated to either FITC or Texas Red at room temperature followed by three washes with PBS. Cells were then counterstained with DAPI at room temperature for 5 minutes to visualize nuclear DNA. Slide or cell images were viewed with a microscope equipped with a digital camera (BX53, OLYMPUS, Shanghai, China). And the colocalization of EZH2 and α-SMA was viewed with confocal microscopy (SP8, Leica, Shanghai, China).

**Statistical Analyses**

All of the experiments were conducted at least three times. Data depicted in graphs represented the means ± SEM for each group. Student’s t-test was employed for comparisons between two groups and one-way analysis of variance (ANOVA) followed by Tukey’s post-test for multiple comparisons was used for groups of three or more. Correlation test analysis was used to study the relationship between EZH2 and clinical parameters. Pearson correlation analysis for bivariate normal distribution and Spearman correlation analysis for non-normal distribution data. All tests were two-tailed, and *P*<0.05 was considered statistically significant. The statistical analyses were conducted by using IBM SPSS Statistics 20.0 (Beijing, China).

**Supplementary Reference：**

1. Xiao L, Zhou D, Tan RJ, et al. Sustained Activation of Wnt/β-Catenin Signaling Drives AKI to CKD Progression. *J Am Soc Nephrol*. 2016;27:1727-40.

2. Wen X, Peng Z, Li Y, et al. One dose of cyclosporine A is protective at initiation of folic acid-induced acute kidney injury in mice. *Nephrol Dial Transplant*. 2012;27:3100-9.

3. Liu N, Wang L, Yang T, et al. EGF Receptor Inhibition Alleviates Hyperuricemic Nephropathy. *J Am Soc Nephrol*. 2015;26:2716-29.

1. **Supplementary Figures**


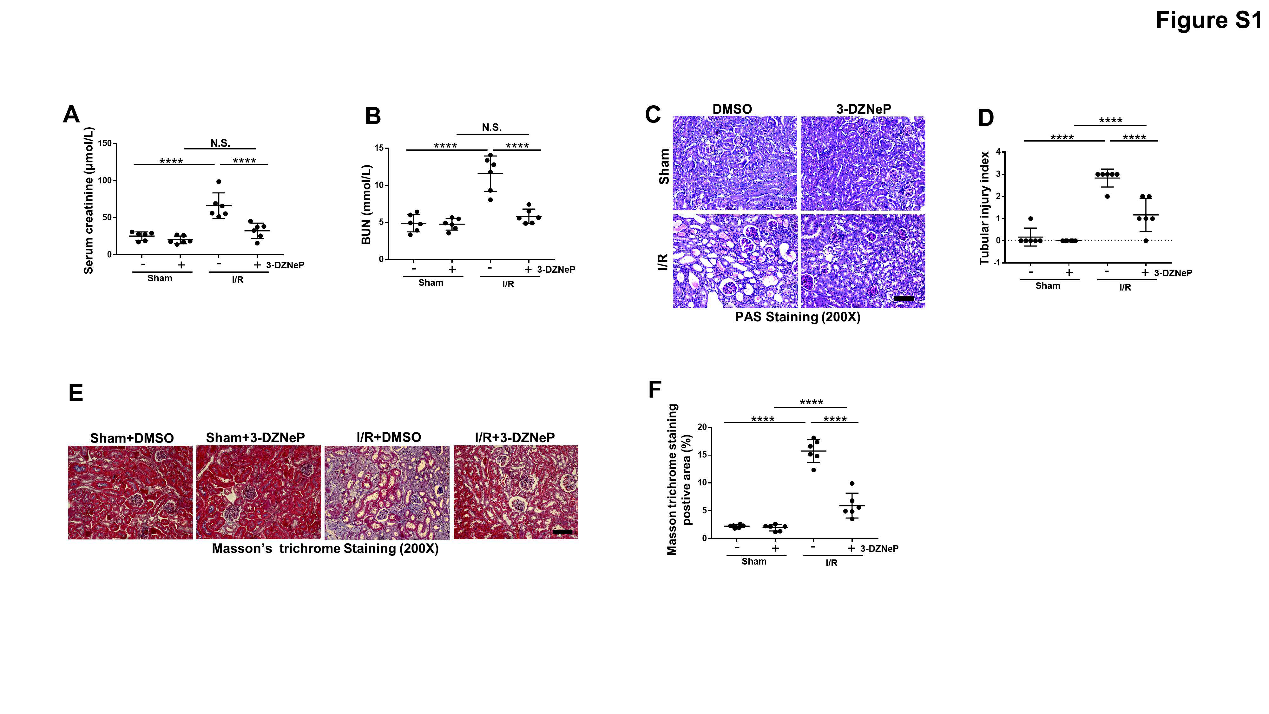
**Supplementary Fig. 1. 3-DZNeP preserves renal function and suppresses renal fibrosis in I/R induced AKI-to-CKD transition mouse model**

The mouse model of AKI-to-CKD transition induced by I/R was established by clipping the bilateral renal arteries of mice for 30 minutes, and the animals were observed and fed for four weeks after operation. (A) Serum creatinine of the mice in different groups. (B) BUN of the mice in different groups. (C) Photomicrographs showed the PAS staining of the kidneys. (D) Morphologic change of tubular injury was scored on the basis of PAS staining described in the Method section. (E) Photomicrographs showed the Masson’s trichrome staining of the kidneys. (F) The graph showed the positive areas (blue) of Masson’s trichrome staining. Data were expressed as means ± SEM. **P*<0.05; ***P*<0.01; ****P*<0.001; *****P*<0.0001. N.S., statistically not significant, with the comparisons labeled. All scale bars = 50 μm.


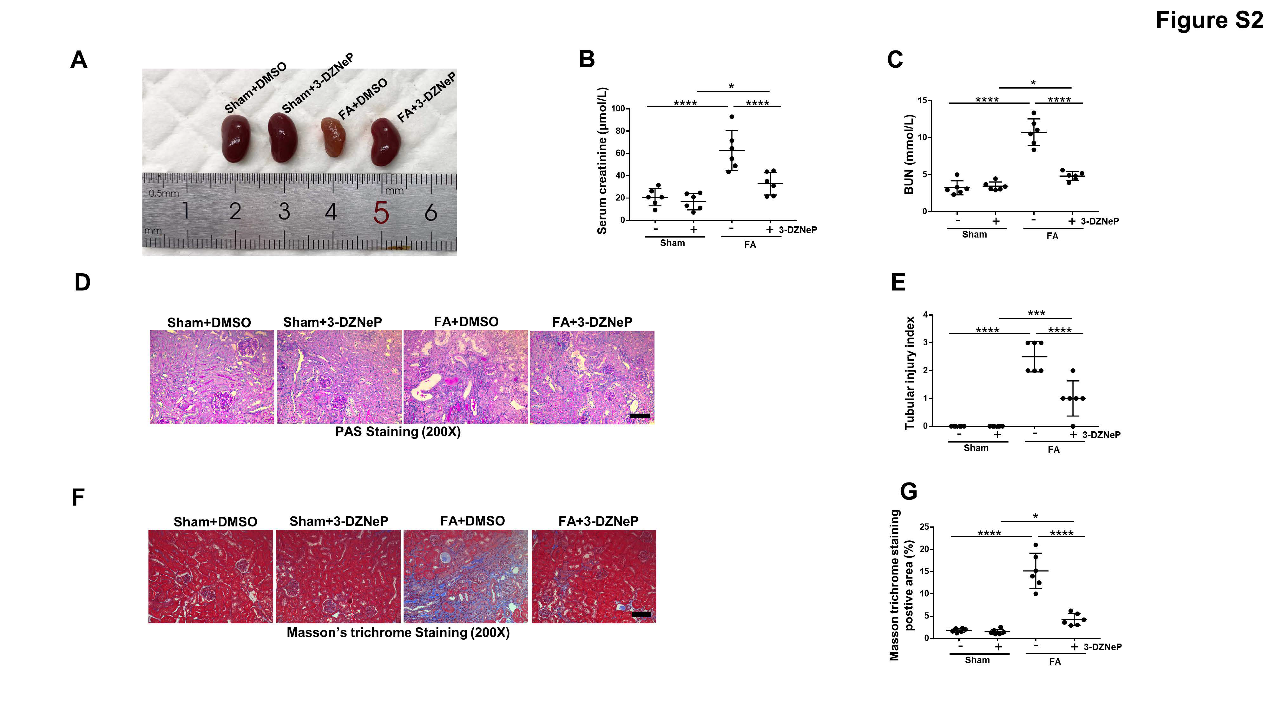


**Supplementary Fig. 2. 3-DZNeP preserves renal function and suppresses renal fibrosis in FA induced AKI-to-CKD transition mouse model**

The mice in FA model were injected with a single dose of folic acid (250 mg/kg, dissolved in 300 mM NaHCO3, i.p.), while the control group was injected with an identical voluminal vehicle (300 mM NaHCO3, i.p.) for four weeks. (A) Photograph showed the size, color and texture of kidney in each group. (B) Serum creatinine of the mice in different groups. (C) BUN of the mice in different groups. (D) Photomicrographs showed the PAS staining of the kidneys. (E) Morphologic change of tubular injury was scored on the basis of PAS staining described in the Method section. (F) Photomicrographs showed the Masson’s trichrome staining of the kidneys. (G) The graph showed the positive areas (blue) of Masson’s trichrome staining. Data were expressed as means ± SEM. **P*<0.05; ***P*<0.01; ****P*<0.001; *****P*<0.0001. N.S., statistically not significant, with the comparisons labeled. All scale bars = 50 μm.


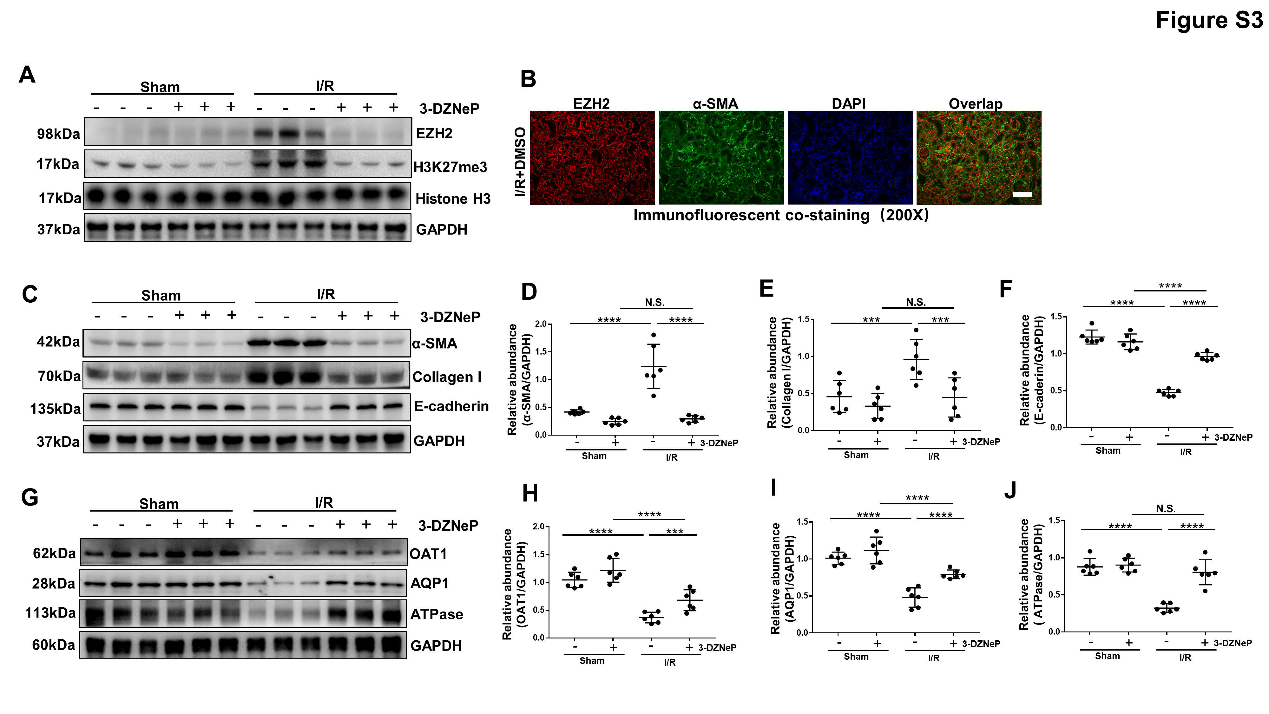


**Supplementary Fig. 3. 3-DZNeP suppresses EMT and loss of TEC transporters in I/R induced AKI-to-CKD transition mouse model**

(A) Kidney tissue lysates from I/R mice were subjected to immunoblotting analysis with specific antibodies against EZH2, H3K27me3, Histone H3 and GAPDH. (B) Photomicrographs showed the immunofluorescent co-staining of EZH2 and α-SMA in IR group. (C) Kidney tissue lysates from I/R mice were subjected to immunoblotting analysis with specific antibodies against α-SMA, Collagen I, E-cadherin and GAPDH. (D-F) Expression levels of α-SMA, Collagen I, E-cadherin in different groups were quantified by densitometry and normalized with GAPDH. (G) Kidney tissue lysates from I/R mice were subjected to immunoblotting analysis with specific antibodies against OAT1, AQP1, ATPase and GAPDH. (H-J) Expression levels of OAT1, AQP1, ATPase in different groups were quantified by densitometry and normalized with GAPDH. Data were expressed as means ± SEM. **P*<0.05; ***P*<0.01; ****P*<0.001; *****P*<0.0001. N.S., statistically not significant, with the comparisons labeled. All scale bars = 50 μm.


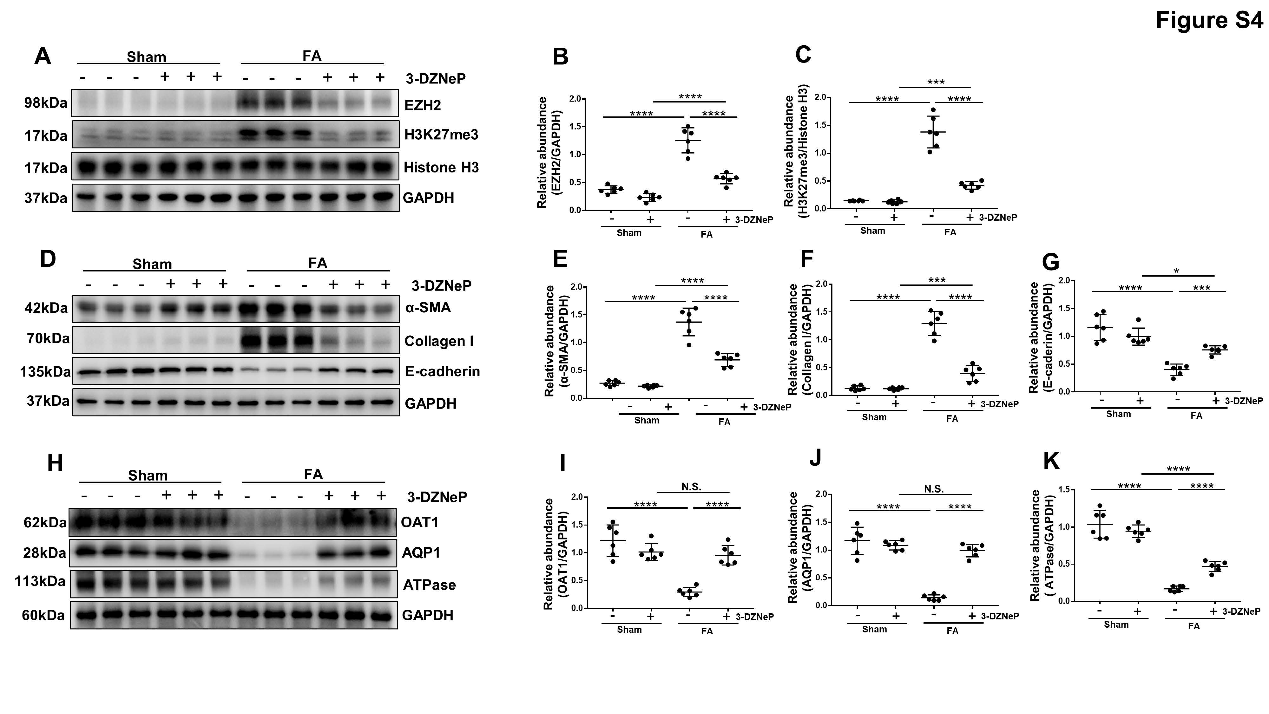


**Supplementary Fig. 4. 3-DZNeP suppresses EMT and loss of TEC transporters in FA induced AKI-to-CKD transition mouse model**

(A) Kidney tissue lysates from FA mice were subjected to immunoblotting analysis with specific antibodies against EZH2, H3K27me3, Histone H3 and GAPDH. (B, C) Expression levels of EZH2 and H3K27me3 in different groups were quantified by densitometry and normalized with GAPDH and Histone H3 respectively. (D) Kidney tissue lysates from FA mice were subjected to immunoblotting analysis with specific antibodies against α-SMA, Collagen I, E-cadherin and GAPDH. (E-G) Expression levels of α-SMA, Collagen I, E-cadherin in different groups were quantified by densitometry and normalized with GAPDH. (H) Kidney tissue lysates from FA mice were subjected to immunoblotting analysis with specific antibodies against OAT1, AQP1, ATPase and GAPDH. (I-K) Expression levels of OAT1, AQP1, ATPase in different groups were quantified by densitometry and normalized with GAPDH. Data were expressed as means ± SEM. **P*<0.05; ***P*<0.01; ****P*<0.001; *****P*<0.0001. N.S., statistically not significant, with the comparisons labeled.


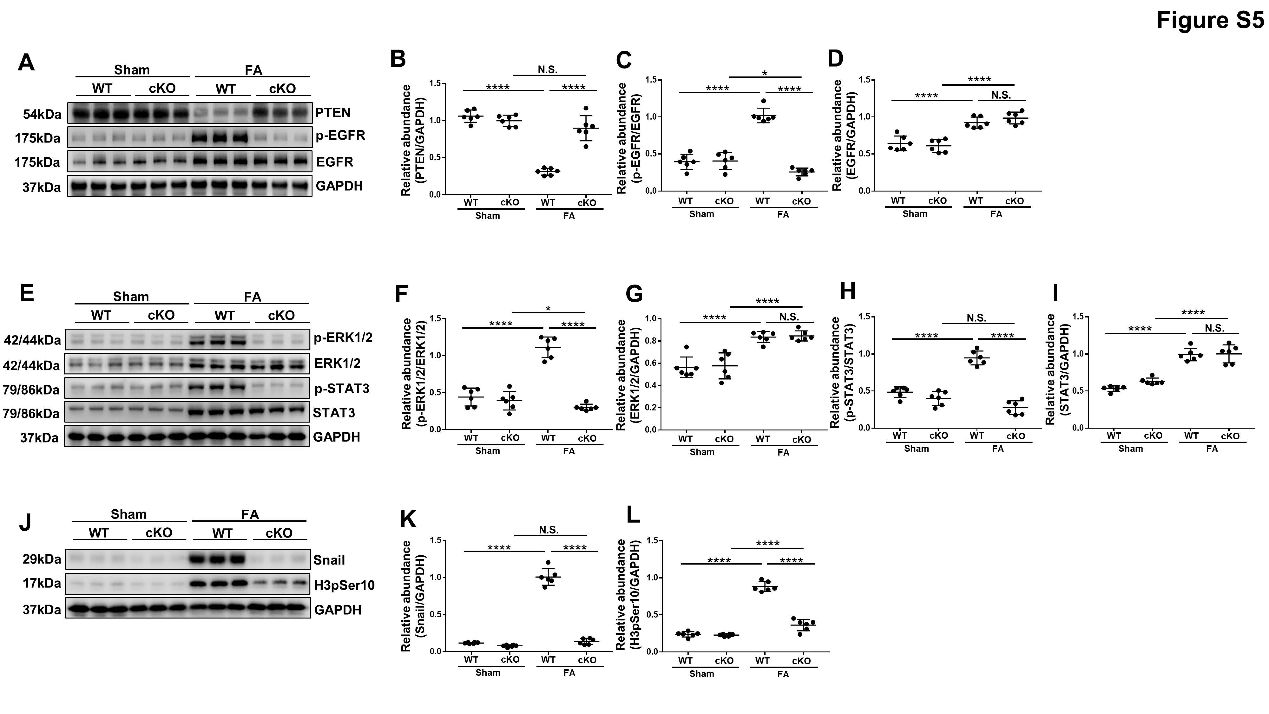
 **Supplementary Fig. 5. EZH2 conditional knockout upregulates the expression of PTEN, thus blocking the activation of EGFR/ERK1/2/STAT3 signaling pathway in FA induced AKI-to-CKD transition mouse model**

(A) Kidney tissue lysates from FA mice were prepared and subjected to immunoblotting analysis with antibodies against PTEN, p-EGFR, EGFR and GAPDH. (B-D) Expression levels of PTEN, p-EGFR, EGFR in different groups were quantified by densitometry and normalized with GAPDH and EGFR respectively. (E) Kidney tissue lysates from FA mice were prepared and subjected to immunoblotting analysis with antibodies against p-ERK1/2, ERK1/2, p-STAT3, STAT3 and GAPDH. (F-I) Expression levels of p-ERK1/2, ERK1/2, p-STAT3, STAT3 in different groups were quantified by densitometry and normalized with GAPDH, ERK1/2 and STAT3 respectively. (J) Kidney tissue lysates from FA mice were prepared and subjected to immunoblotting analysis with antibodies against Snail, H3pSer10 and GAPDH. (K, L) Expression levels of Snail and H3pSer10 in different groups were quantified by densitometry and normalized with GAPDH. Data were expressed as means ± SEM. **P*<0.05; ***P*<0.01; ****P*<0.001; *****P*<0.0001. N.S., statistically not significant, with the comparisons labeled.


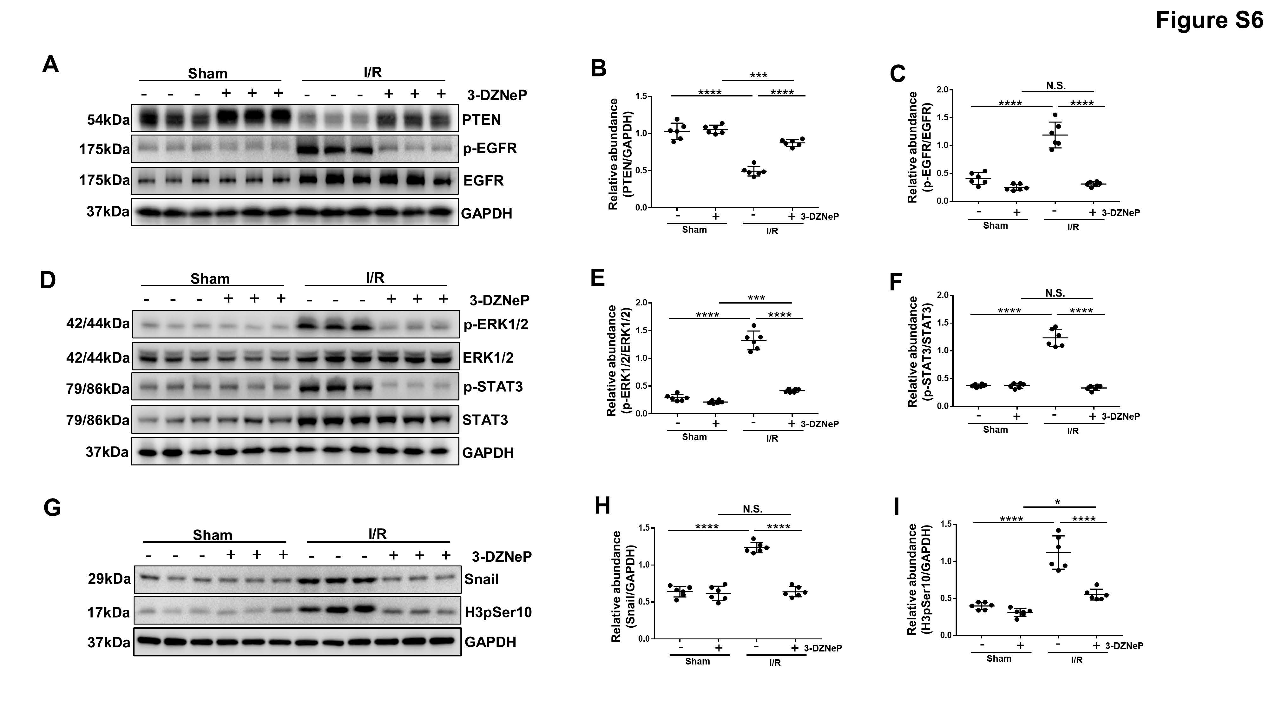


**Supplementary Fig. 6. 3-DZNeP preserves the expression of PTEN, thus suppressing the activation of EGFR/ERK1/2/STAT3 signaling pathway in I/R induced AKI-to-CKD transition mouse model**

(A) Kidney tissue lysates from I/R mice were prepared and subjected to immunoblotting analysis with antibodies against PTEN, p-EGFR, EGFR and GAPDH. (B, C) Expression levels of PTEN and p-EGFR in different groups were quantified by densitometry and normalized with GAPDH and EGFR respectively. (D) Kidney tissue lysates from I/R mice were prepared and subjected to immunoblotting analysis with antibodies against p-ERK1/2, ERK1/2, p-STAT3, STAT3 and GAPDH. (E, F) Expression levels of p-ERK1/2 and p-STAT3 in different groups were quantified by densitometry and normalized with ERK1/2 and STAT3 respectively. (G) Kidney tissue lysates from I/R mice were prepared and subjected to immunoblotting analysis with antibodies against Snail, H3pSer10 and GAPDH. (H, I) Expression levels of Snail and H3pSer10 in different groups were quantified by densitometry and normalized with GAPDH. Data were expressed as means ± SEM. **P*<0.05; ***P*<0.01; ****P*<0.001; *****P*<0.0001. N.S., statistically not significant, with the comparisons labeled.


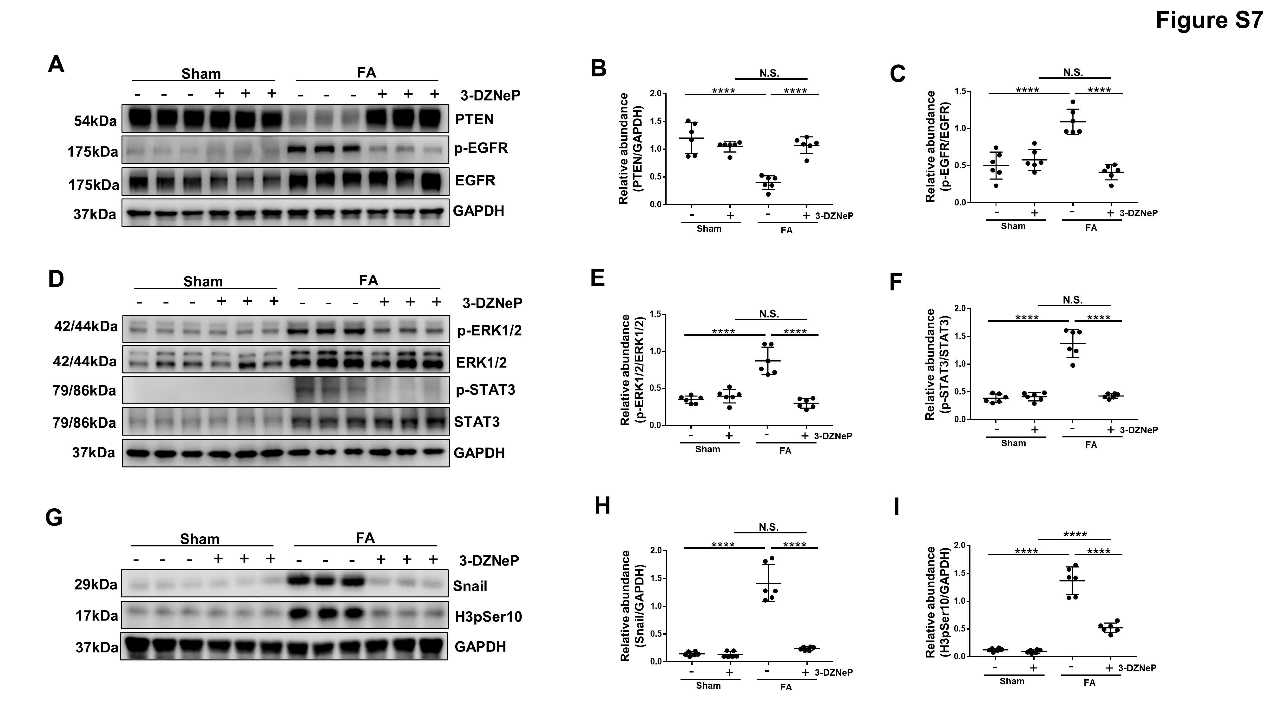


**Supplementary Fig. 7. 3-DZNeP preserves the expression of PTEN, thus suppressing the activation of EGFR/ERK1/2/STAT3 signaling pathway in FA induced AKI-to-CKD transition mouse model**

(A) Kidney tissue lysates from FA mice were prepared and subjected to immunoblotting analysis with antibodies against PTEN, p-EGFR, EGFR and GAPDH. (B, C) Expression levels of PTEN and p-EGFR in different groups were quantified by densitometry and normalized with GAPDH and EGFR respectively. (D) Kidney tissue lysates from FA mice were prepared and subjected to immunoblotting analysis with antibodies against p-ERK1/2, ERK1/2, p-STAT3, STAT3 and GAPDH. (E, F) Expression levels of p-ERK1/2 and p-STAT3 in different groups were quantified by densitometry and normalized with ERK1/2 and STAT3 respectively. (G) Kidney tissue lysates from FA mice were prepared and subjected to immunoblotting analysis with antibodies against Snail, H3pSer10 and GAPDH. (H, I) Expression levels of Snail and H3pSer10 in different groups were quantified by densitometry and normalized with GAPDH. Data were expressed as means ± SEM. **P*<0.05; ***P*<0.01; ****P*<0.001; *****P*<0.0001. N.S., statistically not significant, with the comparisons labeled.


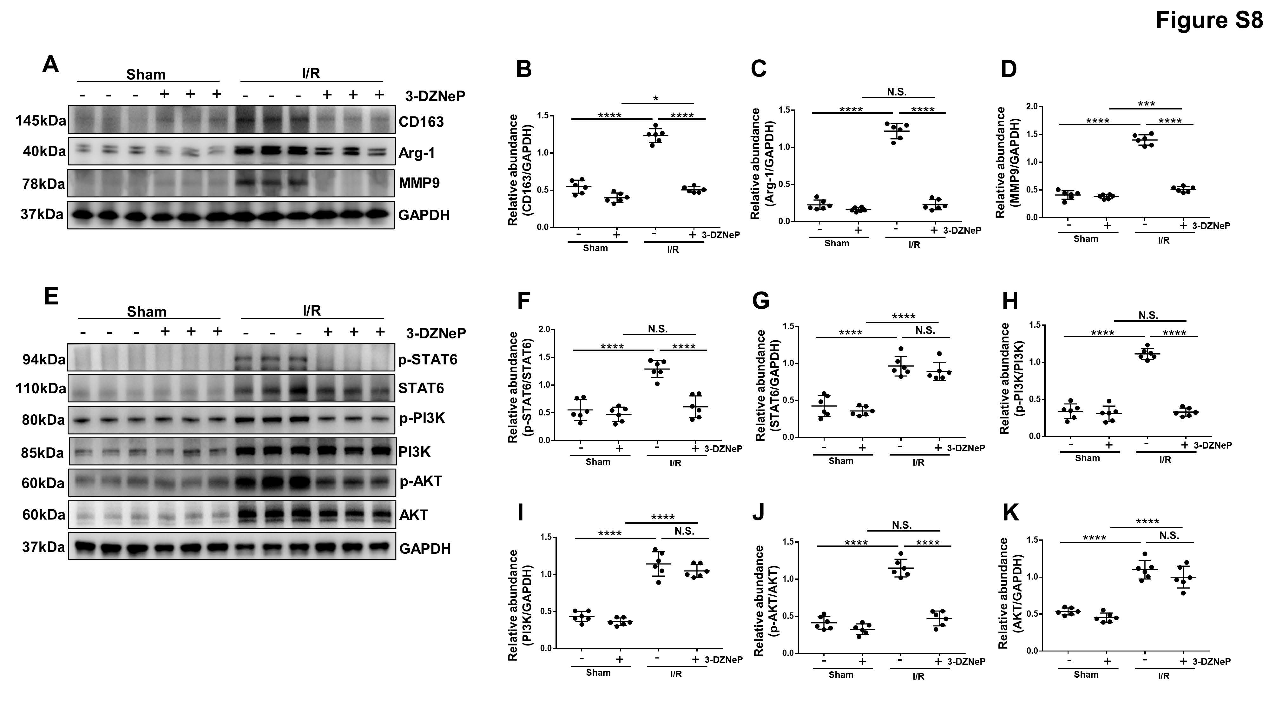


**Supplementary Fig. 8. 3-DZNeP reduces M2 macrophage polarization through STAT6 and PI3K/AKT pathways in I/R induced AKI-to-CKD transition mouse model**

(A) Kidney tissue lysates from I/R mice were prepared and subjected to immunoblotting analysis with antibodies against CD163, Arginase-1, MMP9 and GAPDH. (B-D) Expression levels of CD163, Arginase-1, MMP9 in different groups were quantified by densitometry and normalized with GAPDH. (E) Kidney tissue lysates from I/R mice were prepared and subjected to immunoblotting analysis with antibodies against p-STAT6, STAT6, p-PI3K, PI3K, p-AKT, AKT and GAPDH. (F-K) Expression levels of p-STAT6, STAT6, p-PI3K, PI3K, p-AKT, AKT were quantified by densitometry and normalized with GAPDH, STAT6, PI3K and AKT. Data were expressed as means ± SEM. **P*<0.05; ***P*<0.01; ****P*<0.001; *****P*<0.0001. N.S., statistically not significant, with the comparisons labeled.


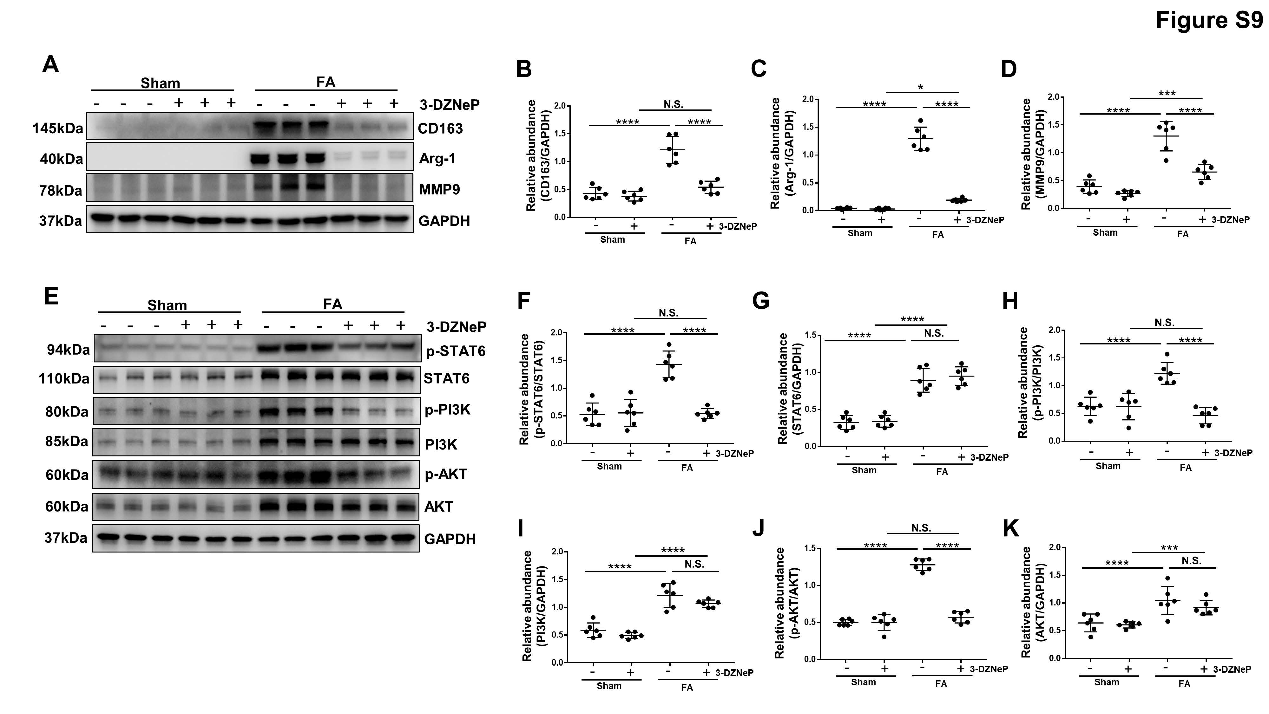


**Supplementary Fig. 9. 3-DZNeP reduces M2 macrophage polarization through STAT6 and PI3K/AKT pathways in FA induced AKI-to-CKD transition mouse model**

(A) Kidney tissue lysates from FA mice were prepared and subjected to immunoblotting analysis with antibodies against CD163, Arginase-1, MMP9 and GAPDH. (B-D) Expression levels of CD163, Arginase-1, MMP9 in different groups were quantified by densitometry and normalized with GAPDH. (E) Kidney tissue lysates from FA mice were prepared and subjected to immunoblotting analysis with antibodies against p-STAT6, STAT6, p-PI3K, PI3K, p-AKT, AKT and GAPDH. (F-K) Expression levels of p-STAT6, STAT6, p-PI3K, PI3K, p-AKT, AKT were quantified by densitometry and normalized with GAPDH, STAT6, PI3K and AKT. Data were expressed as means ± SEM. **P*<0.05; ***P*<0.01; ****P*<0.001; *****P*<0.0001. N.S., statistically not significant, with the comparisons labeled.


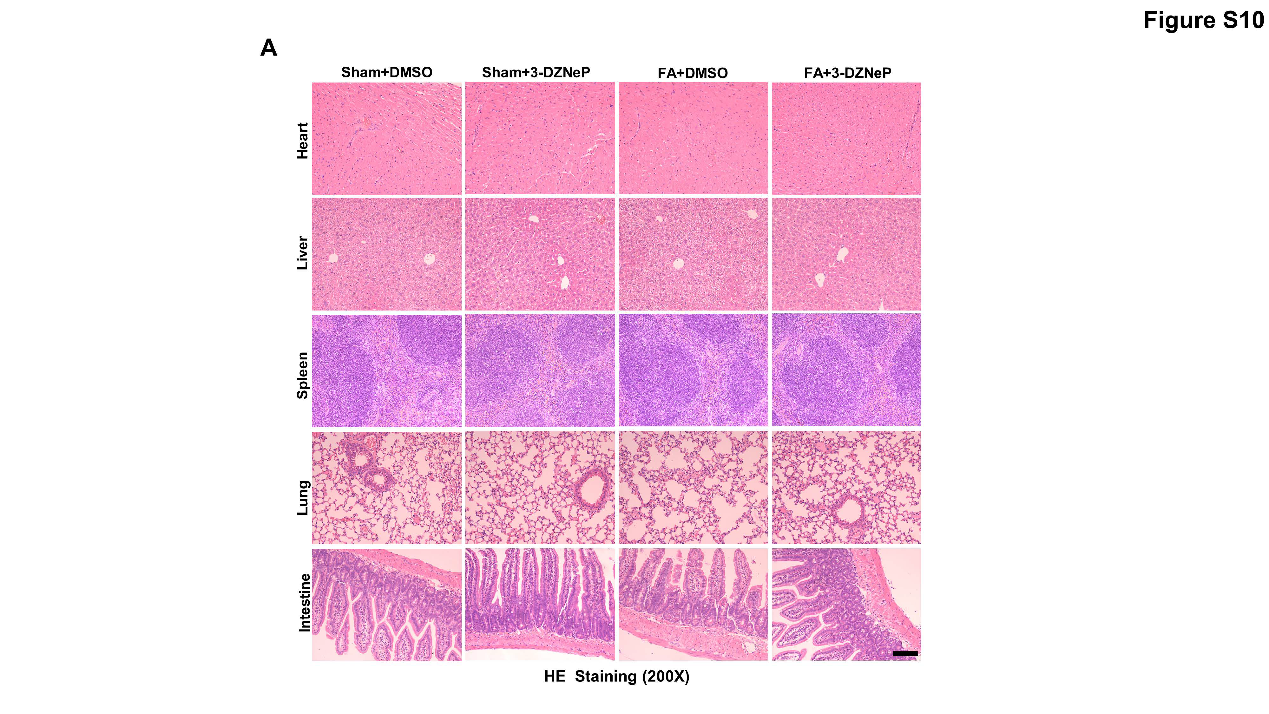


**Supplementary Fig. 10. Treatment with 3-DZNeP has no influence on other organs in FA mouse model.**

(A) HE staining of heart, liver, spleen, lung and intestine from each group in FA mouse model. Scale bars=50μm.

1. **Supplementary Tables**

**Supplementary Table 1. Clinical characteristics of ANCA-associated glomerulonephritis patients and control group**

| **Variables** | **Control group (n=9)** | **ANCA-associated glomerulonephritis (n=9)** | **P value** |
| --- | --- | --- | --- |
| General data |  |  |  |
| Male | 4（44.4%） | 5（55.6%） | 1.000 |
| Age | 68.11±7.06 | 66±8.6 | 0.587 |
| BMI (kg/m^2^) | 24.04±2.79 | 21.6±2.59 | 0.112 |
| Smoking | 2（22.2%） | 3（33.3%） | 0.332 |
| Drinking | 1（11.1%） | 2（22.2%） | 0.231 |
| Laboratory examination |  |  |  |
| Glucose (mmol/L) | 5.49±0.85 | 4.81±0.76 | 0.163 |
| Glycosylated hemoglobin (%) | 6.09±0.54 | 5.45±1.03 | 0.157 |
| Hemoglobin (g/L) | 130.63±17.4 | 96.67±13.38 | 0.002 |
| Albumin (g/L) | 42.84±4.73 | 35.6±3.96 | 0.010 |
| Creatinine (µmol/L) | 67.88±13.81 | 194.22±97.86 | 0.003 |
| BUN (mmol/L) | 5.06±1.08 | 11.45±4.90 | 0.003 |
| eGFR(ml/min/1.73m^2^) | 83.63±12.00 | 48.57±31.36 | 0.006 |
| Uric acid (µmol/L) | 309.25±74.23 | 382.33±77.19 | 0.066 |
| Cholesterol (mmol/L) | 4.65±0.98 | 4.49±0.72 | 0.779 |
| Triglyceride (mmol/L) | 1.87±0.52 | 1.64±0.88 | 0.650 |
| K^+^ (mmol/L) | 4.31±0.22 | 4.51±0.44 | 0.287 |
| Na^+^ (mmol/L) | 141.63±1.6 | 141.17±1.72 | 0.617 |

Abbreviations: ANCA, antineutrophil cytoplasmic antibody; BMI, body mass index; BUN, urea nitrogen; eGFR, estimated glomerular filtration rate.

**Supplementary Table 2.**

1. **siRNA sequence.**

| Species | siRNA | sequence (5’-3’) |  |
| --- | --- | --- | --- |
| Human | EZH2 siRNA | CAUCGAAAGAGAAAUGGAATT | |
|  | Scramble siRNA | UUCUCCGAACGUGUCACGUTT | |
| Mouse | EZH2 siRNA | AACACUGUUGCACUGGUUCTT | |
|  | Scramble siRNA | UUCUCCGAACGUGUCACGUTT | |

1. **Sequences of custom-designed primers for quantitative PCR**

| Species | Gene | Forward sequence (5' to 3') | Reverse sequence (5' to 3') |
| --- | --- | --- | --- |
| Human | PTEN | AGGTTTCCTCTGGTCCTGGT | CGACGGGAAGACAAGTTCAT |
| Human | DNA spike | GTTCCACTCCTGAAGTGTCAAG | ACTTCCGAGTCACAGGAGAATG |

**Supplementary Table 3. Details of primary antibodies used for Immunoblotting analysis.**

| **Antibody** | **Catalogue number** | **Supplier** | **Dilution** |
| --- | --- | --- | --- |
| EZH2 | #5246 | Cell Signaling Technology (Danvers, MA, USA) | 1:1000 |
| H3K27me3 | #9733 | Cell Signaling Technology (Danvers, MA, USA) | 1:1000 |
| PTEN | #9188 | Cell Signaling Technology (Danvers, MA, USA) | 1:1000 |
| E-cadherin | #14472 | Cell Signaling Technology (Danvers, MA, USA) | 1:1000 |
| Snail | #3879 | Cell Signaling Technology (Danvers, MA, USA) | 1:500 |
| p-EGFR | #3777 | Cell Signaling Technology (Danvers, MA, USA) | 1:1000 |
| EGFR | #4267 | Cell Signaling Technology (Danvers, MA, USA) | 1:1000 |
| p-STAT3 | #9138 | Cell Signaling Technology (Danvers, MA, USA) | 1:1000 |
| STAT3 | #9139 | Cell Signaling Technology (Danvers, MA, USA) | 1:1000 |
| p-ERK1/2 | #4370 | Cell Signaling Technology (Danvers, MA, USA) | 1:1000 |
| ERK1/2 | #4695 | Cell Signaling Technology (Danvers, MA, USA) | 1:1000 |
| PI3K | #4257 | Cell Signaling Technology (Danvers, MA, USA) | 1:1000 |
| p-PI3K | #17366 | Cell Signaling Technology (Danvers, MA, USA) | 1:1000 |
| AKT | #4691 | Cell Signaling Technology (Danvers, MA, USA) | 1:1000 |
| p-AKT | #4060 | Cell Signaling Technology (Danvers, MA, USA) | 1:1000 |
| STAT6 | #5397 | Cell Signaling Technology (Danvers, MA, USA) | 1:1000 |
| p-STAT6 | #56554 | Cell Signaling Technology (Danvers, MA, USA) | 1:1000 |
| GAPDH | sc-32233 | Santa Cruz Biotechnology (Santa Cruz, CA, USA) | 1:2000 |
| Collagen I (A2) | sc-393573 | Santa Cruz Biotechnology (Santa Cruz, CA, USA) | 1:500 |
| AQP1 | GB11310-1 | Servicebio (Wuhan, China) | 1:1000 |
| ATPase | GB11400-1 | Servicebio (Wuhan, China) | 1:1000 |
| Arginase-1 | GB11285 | Servicebio (Wuhan, China) | 1:1000 |
| CD163 | GB11340 | Servicebio (Wuhan, China) | 1:1000 |
| OAT1 | A3184 | ABclonal (Wuhan, China) | 1:500 |
| p-Histone H3 | ab5176 | Abcam (Cambridge, MA, USA) | 1:1000 |
| α-SMA | A2547 | Sigma-Aldrich (St. Louis, MO, USA) | 1:1000 |

**Supplementary Table 4. Details of primary antibodies used for Immunofluorescent and Immunohistochemical staining.**

| **Antibody** | **Catalogue number** | **Supplier** | **Dilution** |
| --- | --- | --- | --- |
| EZH2 | #5246 | Cell Signaling Technology (Danvers, MA, USA) | 1:100 |
| α-SMA | A2547 | Cell Signaling Technology (Danvers, MA, USA) | 1:200 |
| H3K27me3 | #9733 | Cell Signaling Technology (Danvers, MA, USA) | 1:800 |
| PTEN | #9188 | Cell Signaling Technology (Danvers, MA, USA) | 1:200 |
| CD163 | GB11340 | Servicebio (Wuhan, China) | 1:500 |
| E-cadherin | sc-7870 | Santa Cruz Biotechnology (Santa Cruz, CA, USA) | 1:100 |
