# Supplementary material for: Enhancer of zeste homolog 2 promotes renal fibrosis after acute kidney injury by inducing epithelial-mesenchymal transition and activation of M2 macrophage polarization
Source: Cell Death Dis. 2023 Apr 7;14(4):253. doi: 10.1038/s41419-023-05782-4 (PMC10081989; doi:10.1038/s41419-023-05782-4)

Figure 2I

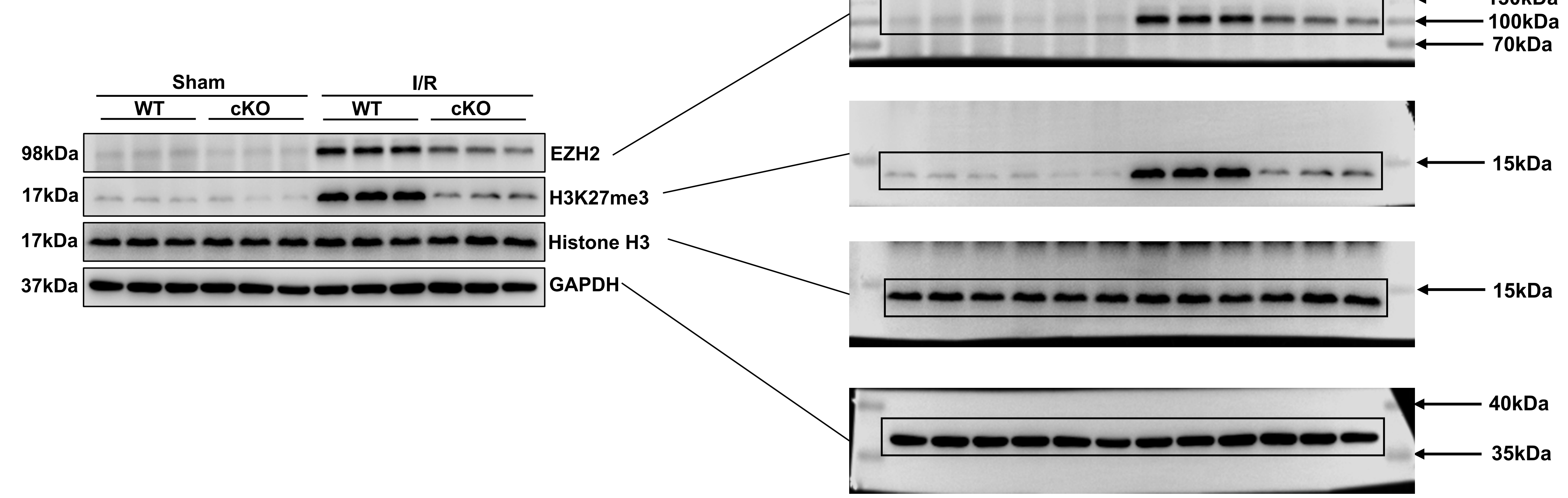

Figure 2L

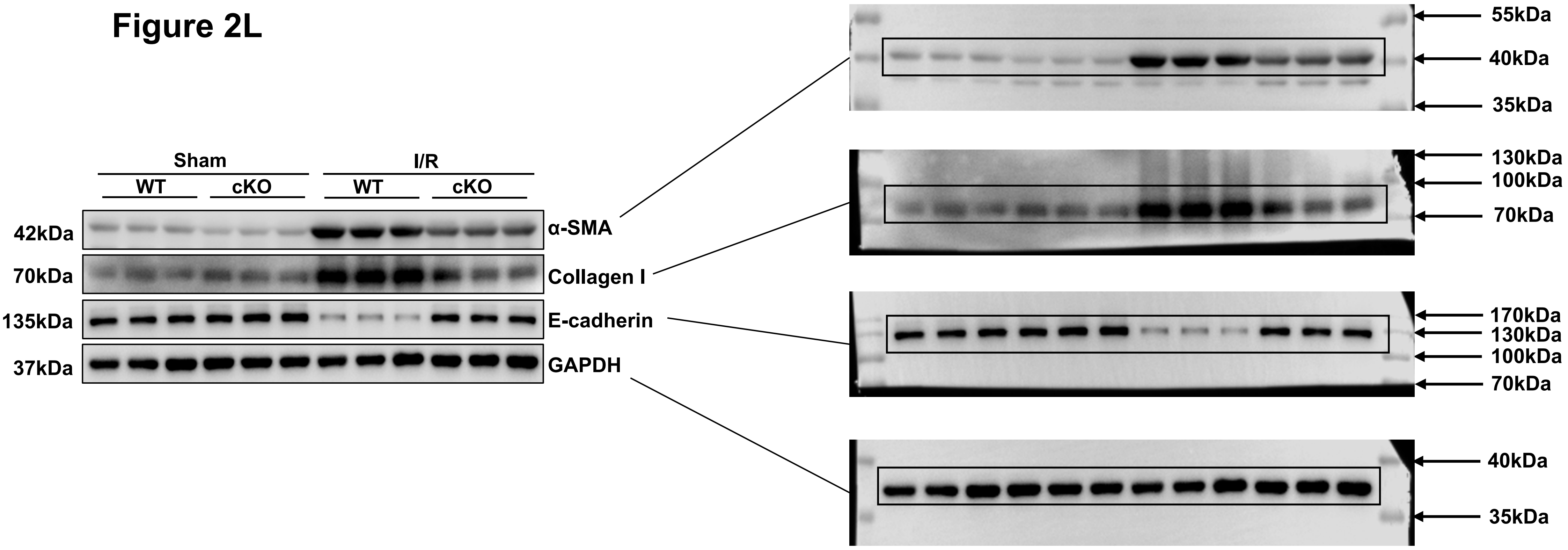

Figure 2P

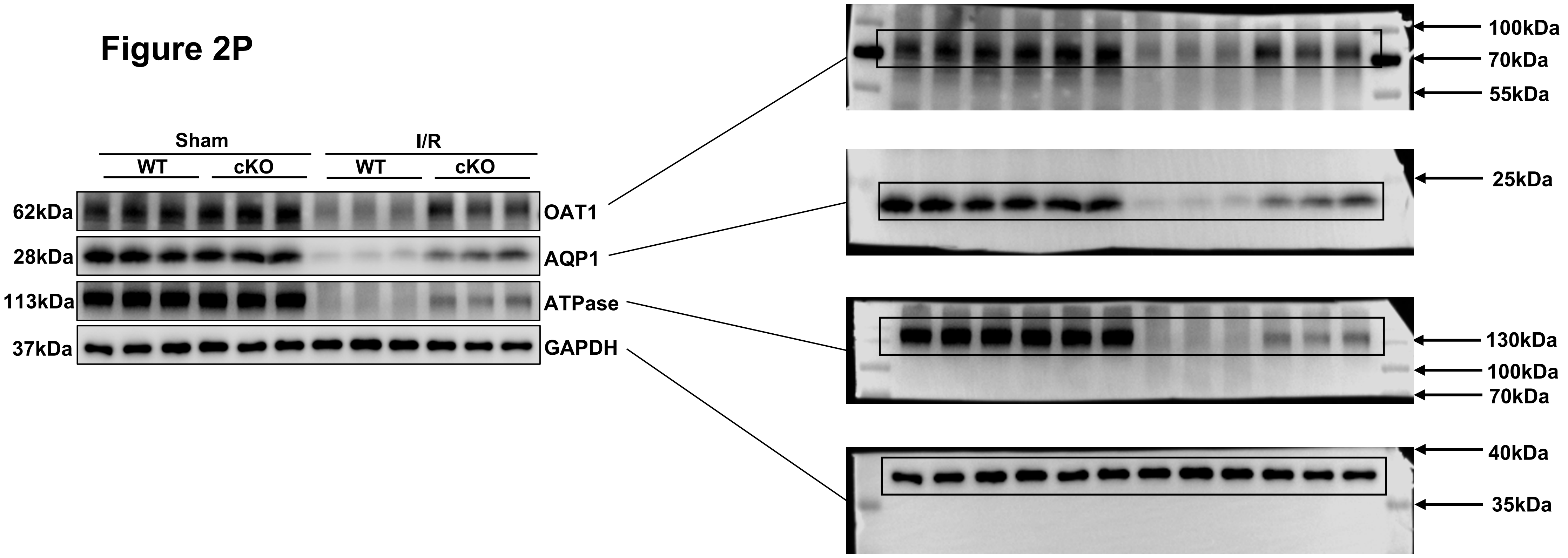

Figure 3H

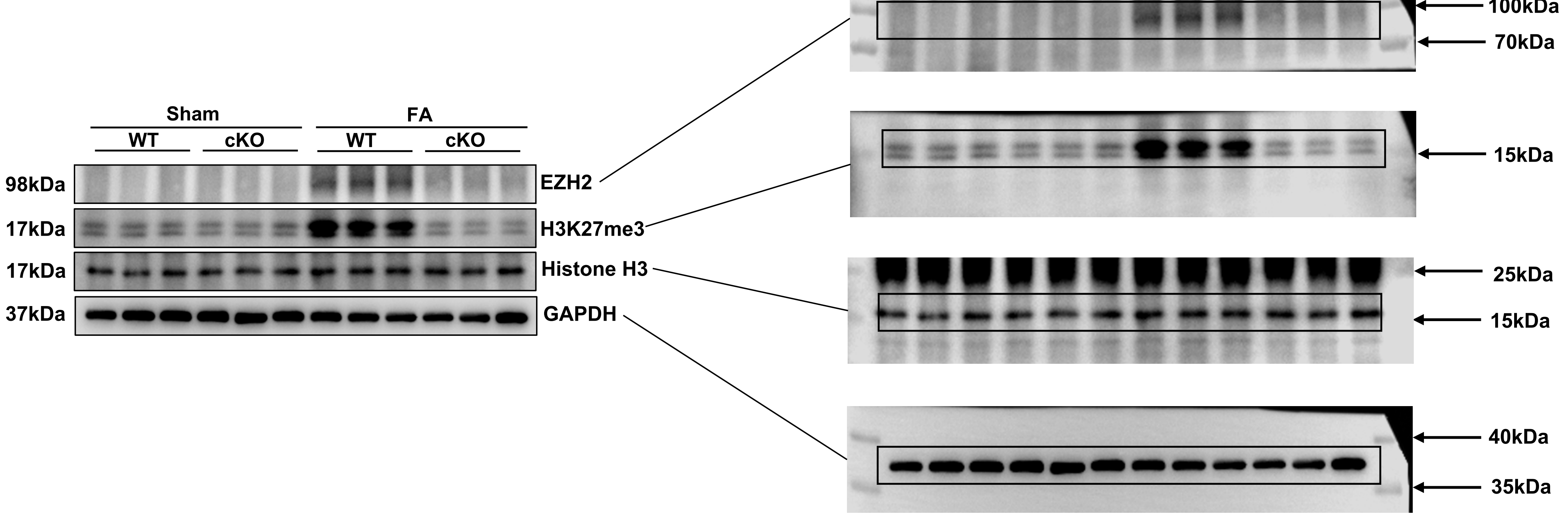

Figure 3K

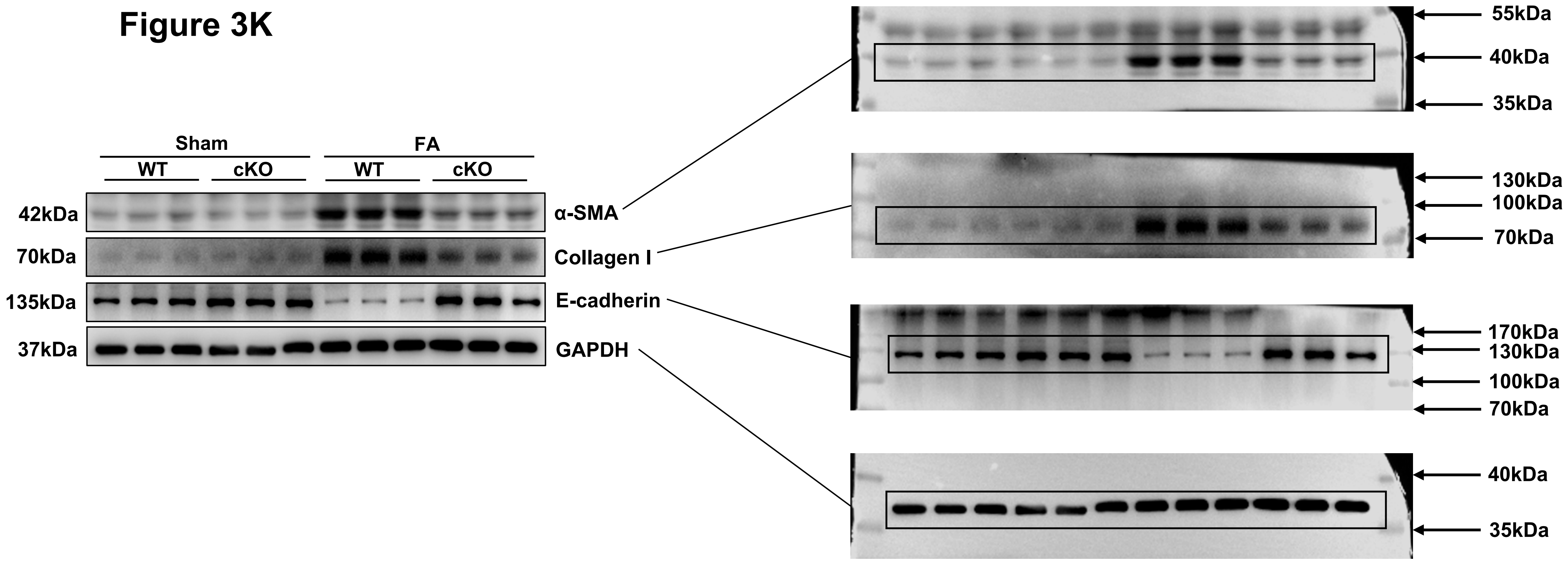

Figure 3O

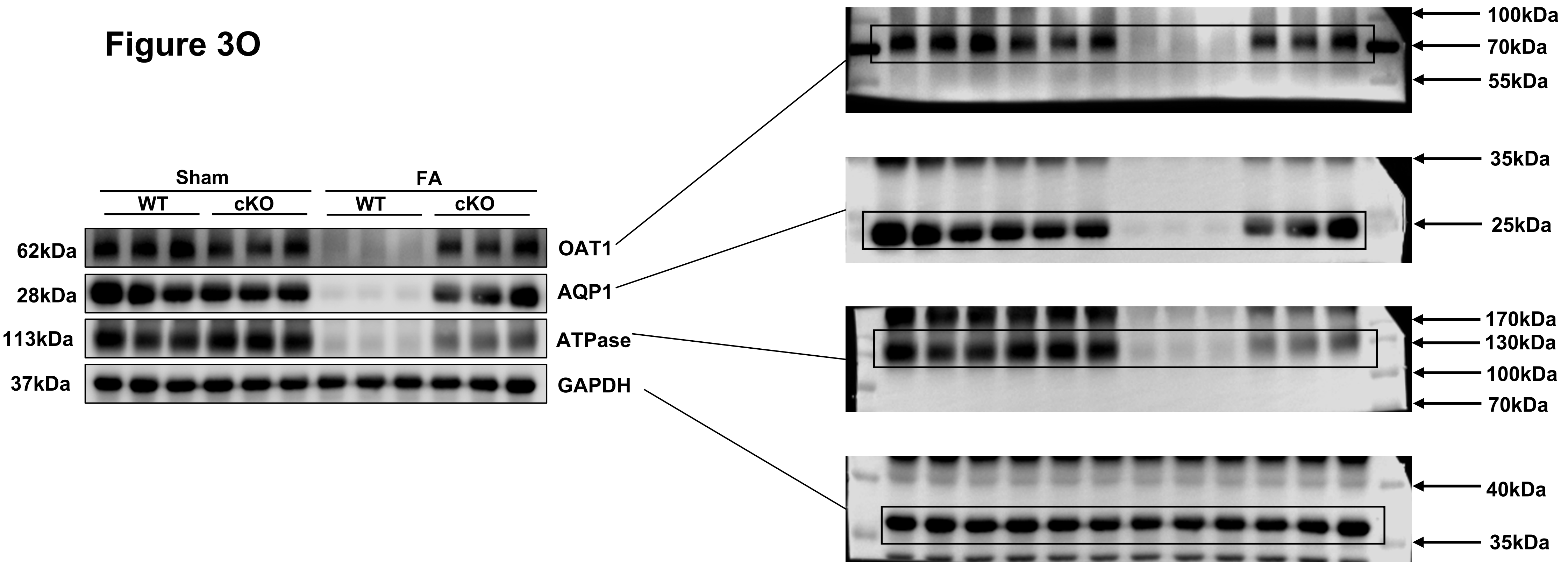

Figure 4A

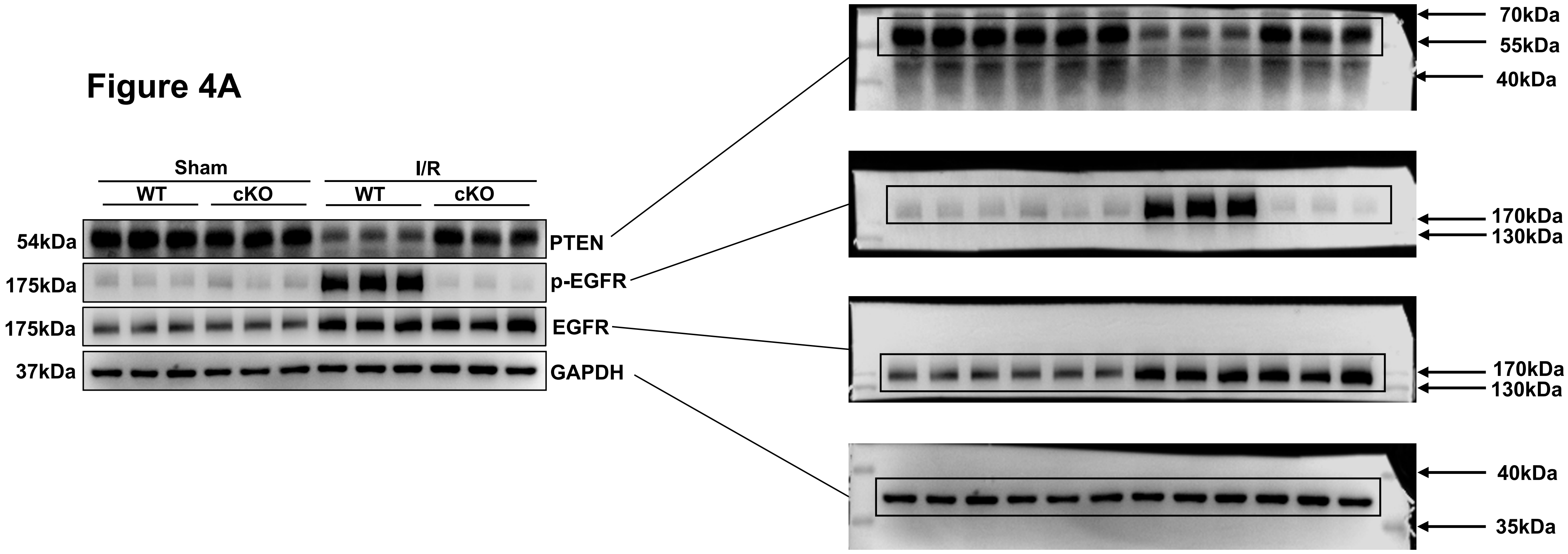

Figure 4F

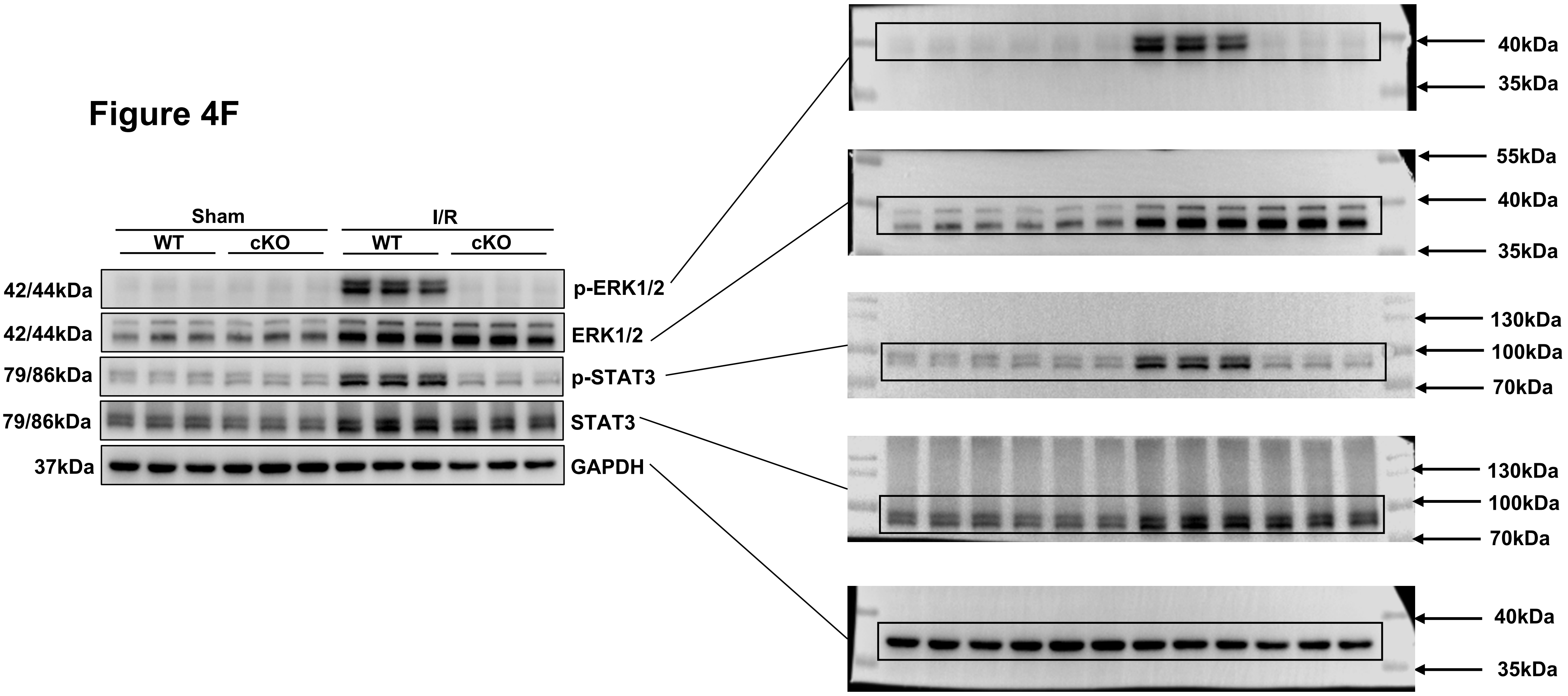

Figure 4K

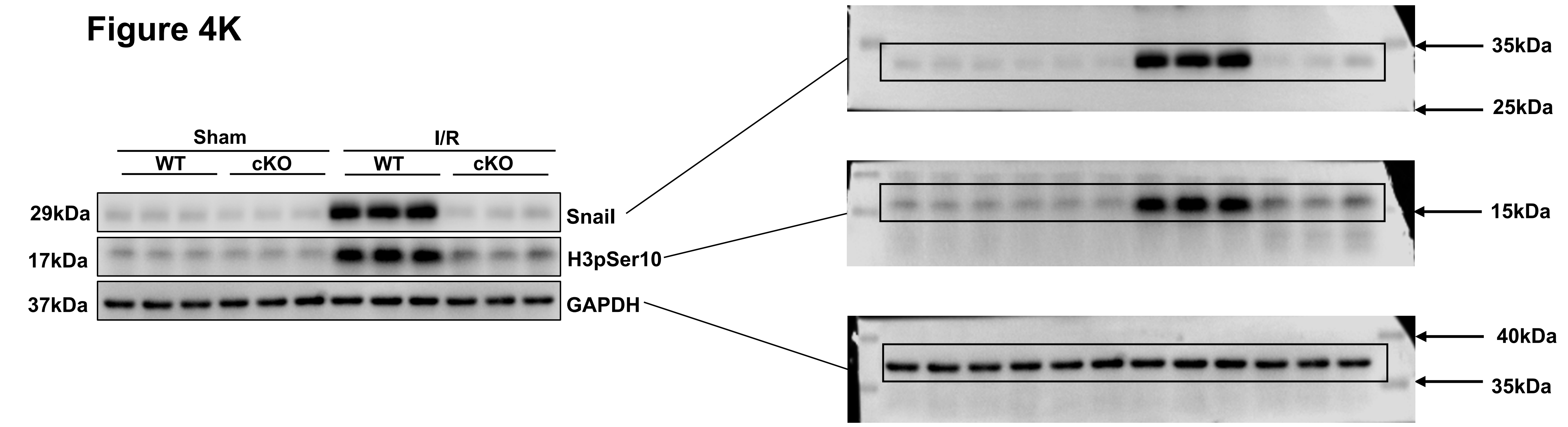

Figure 5A

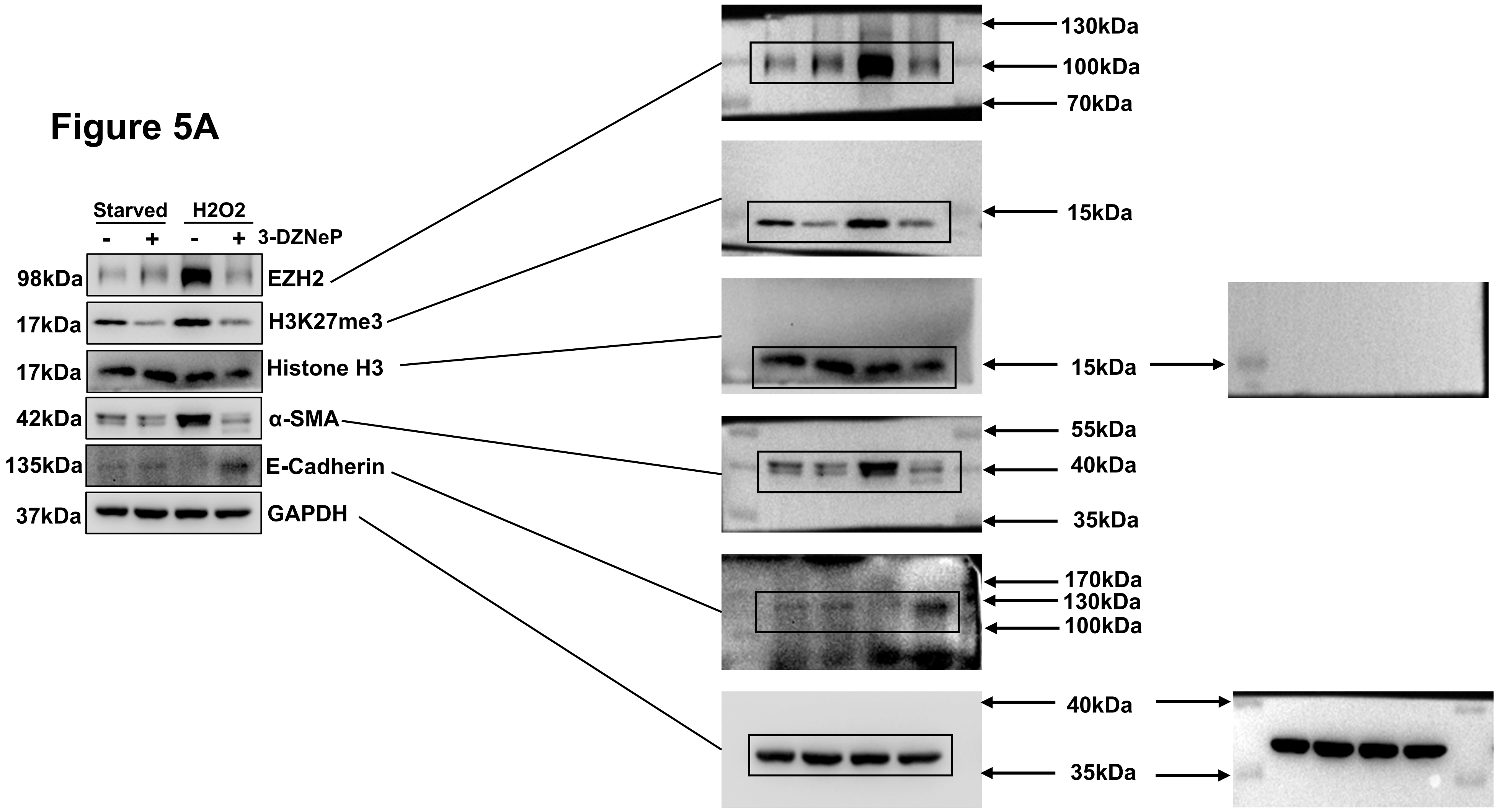

Figure 5F

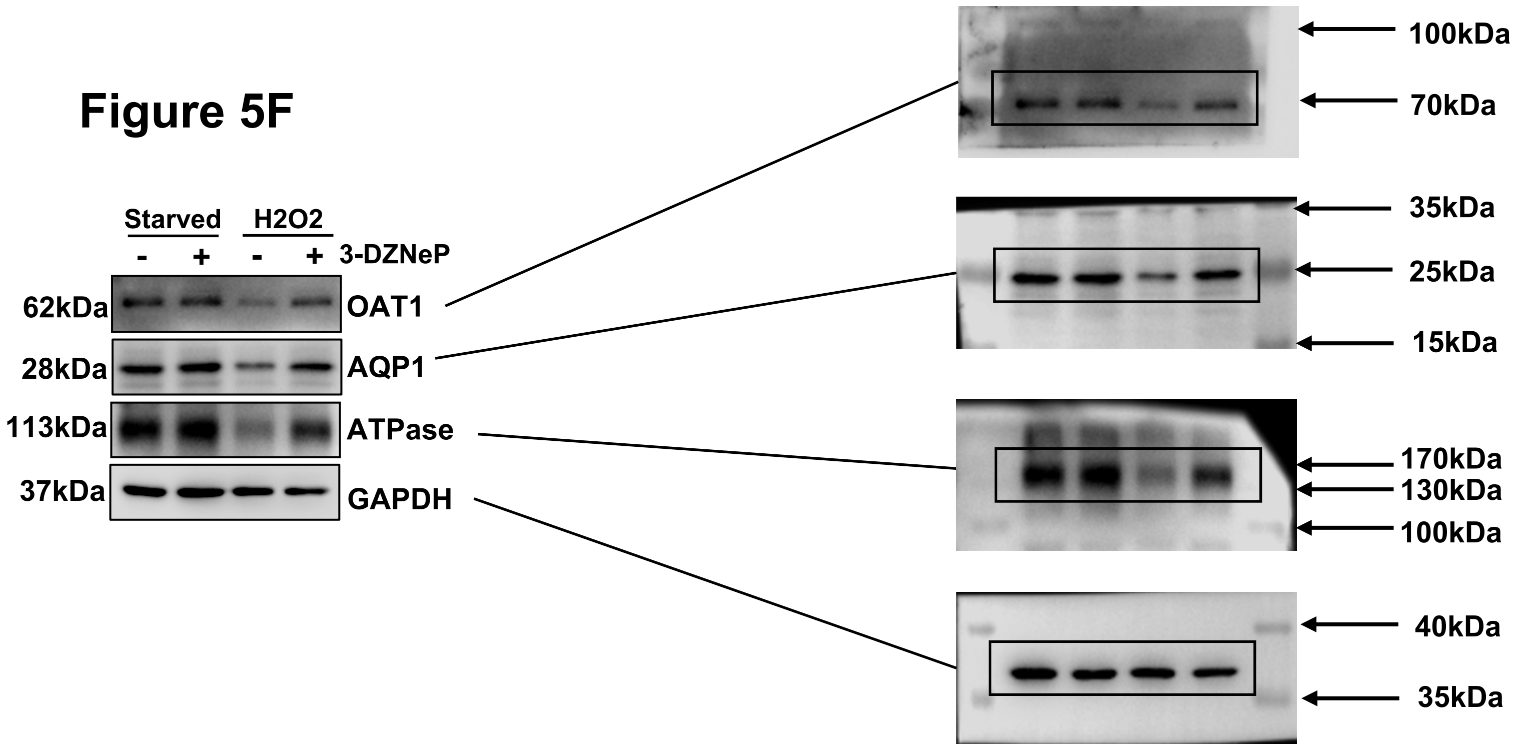

Figure 5K

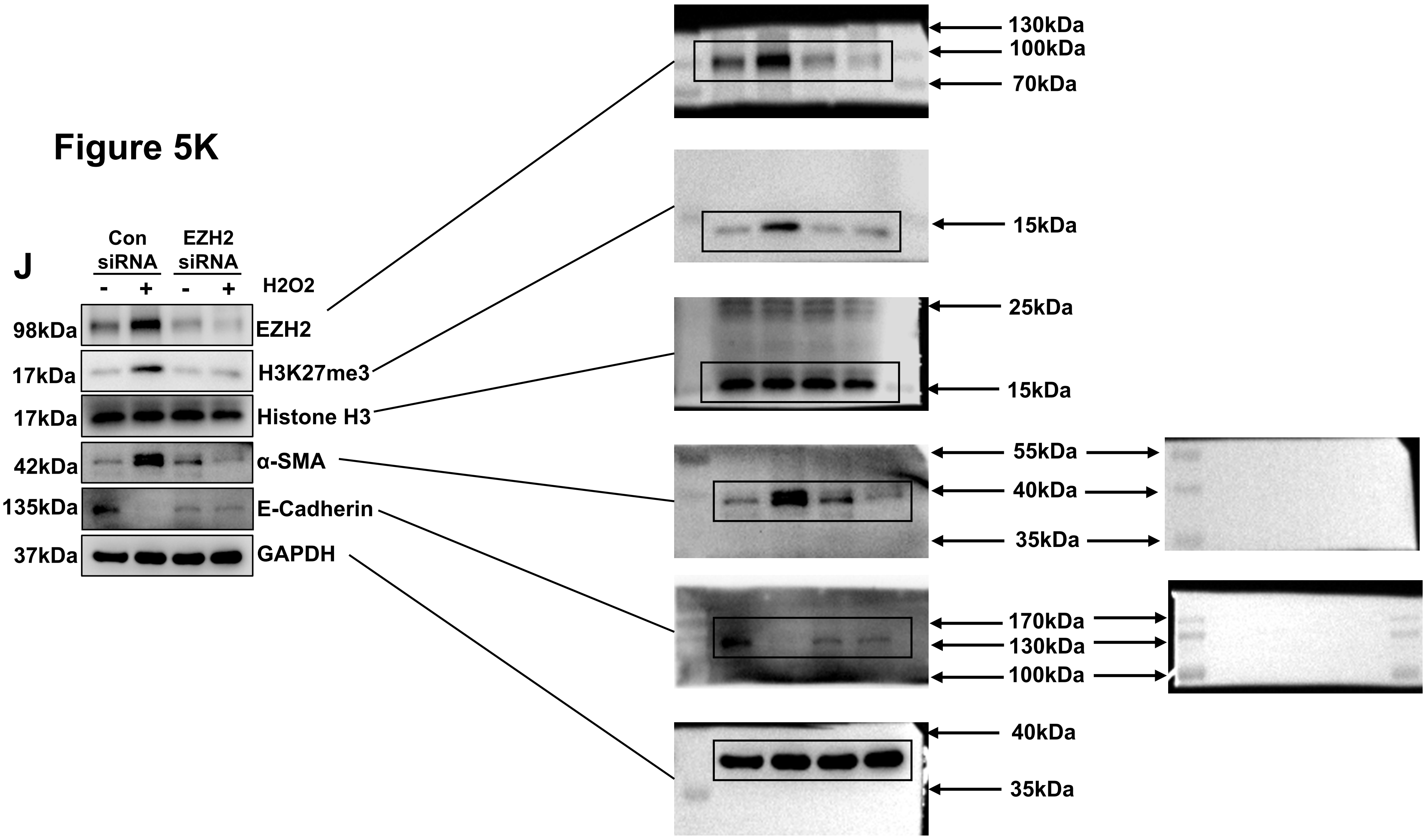

Figure 5P

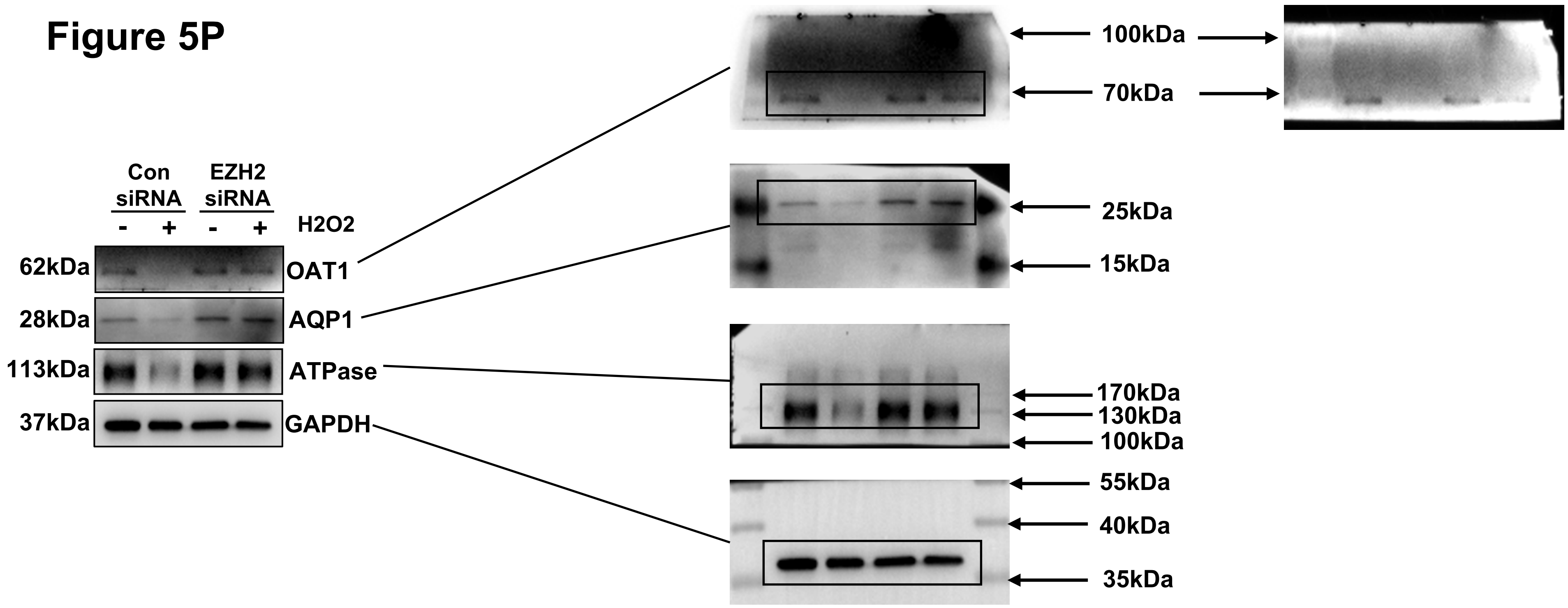

Figure 6A

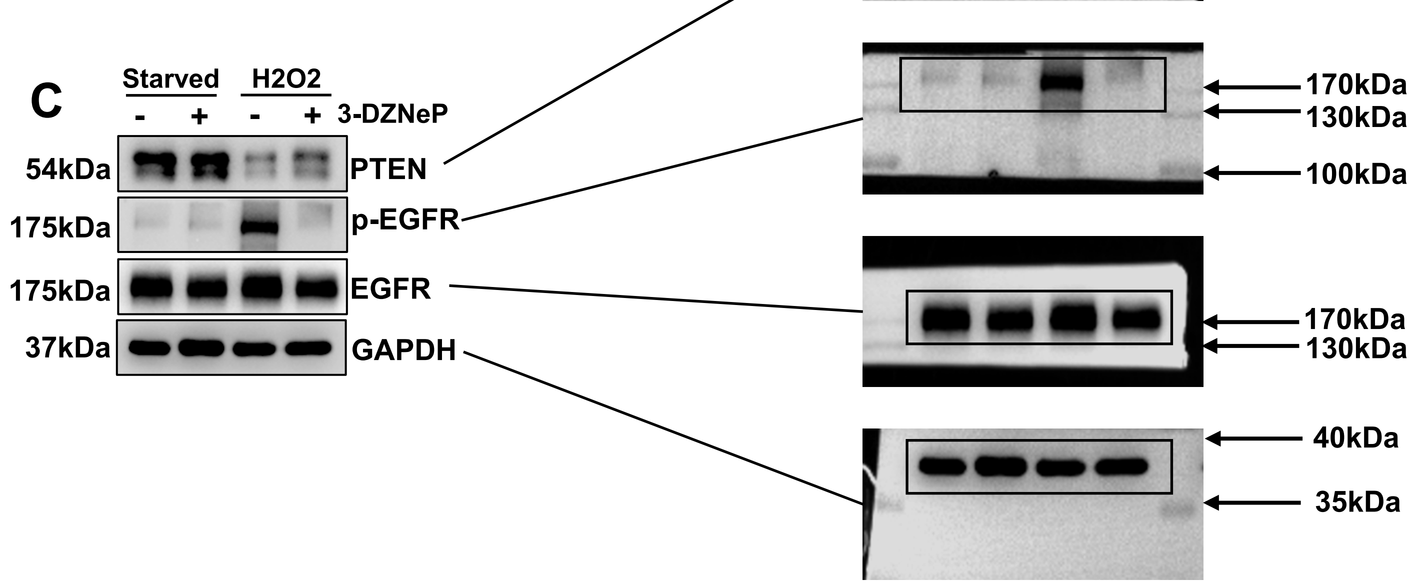

Figure 6D

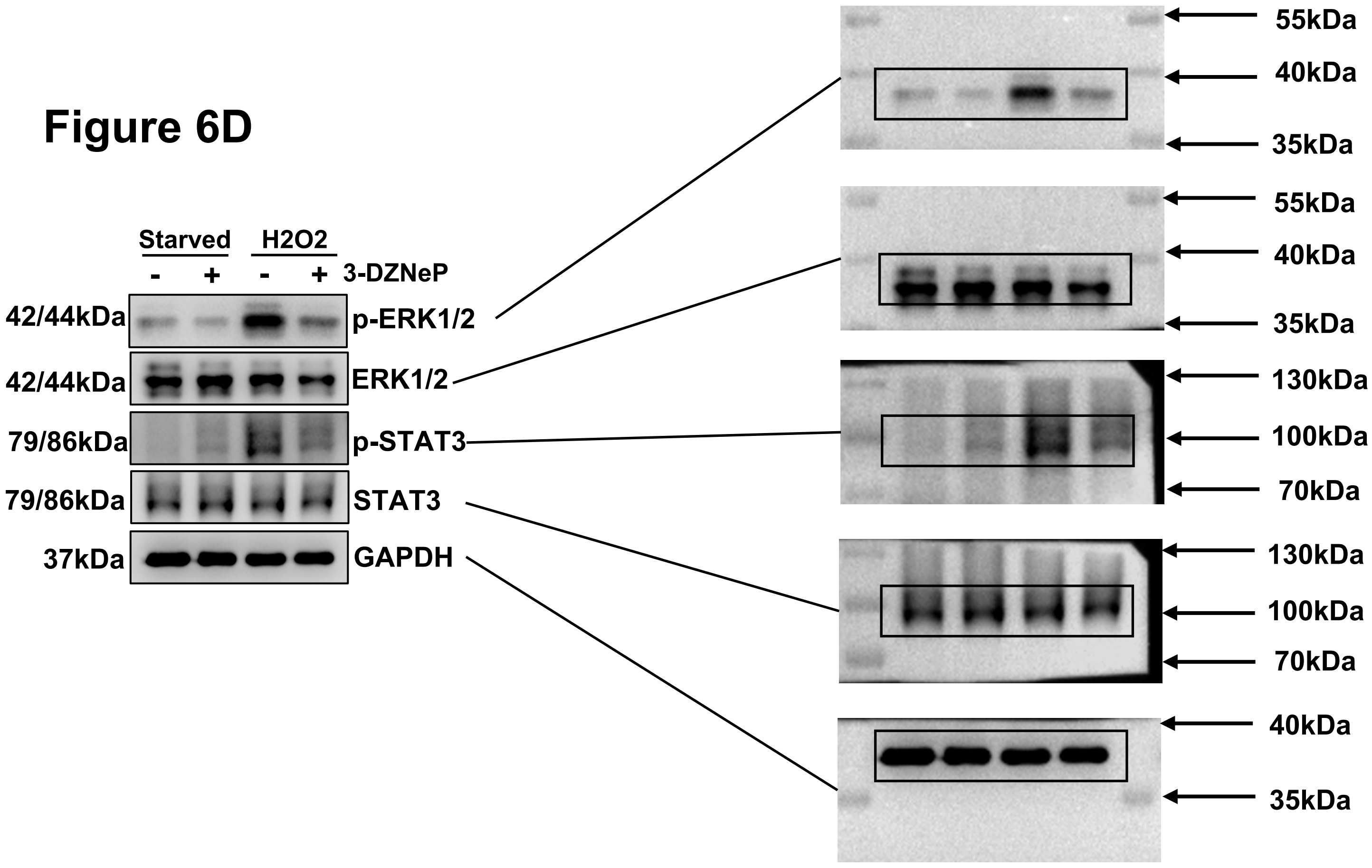

Figure 6G

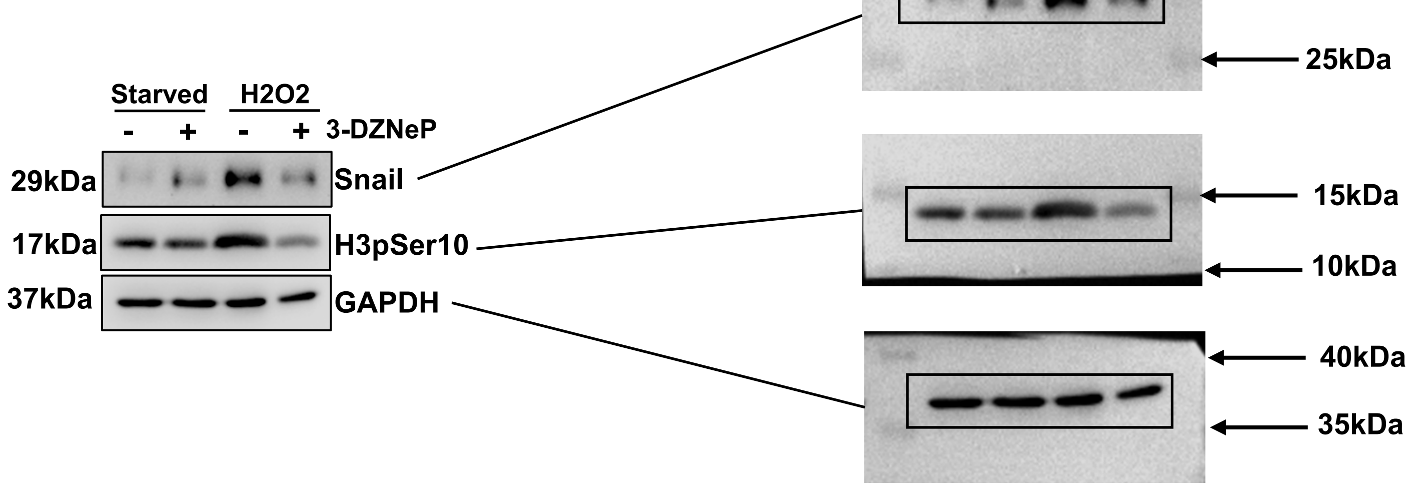

Figure 6J

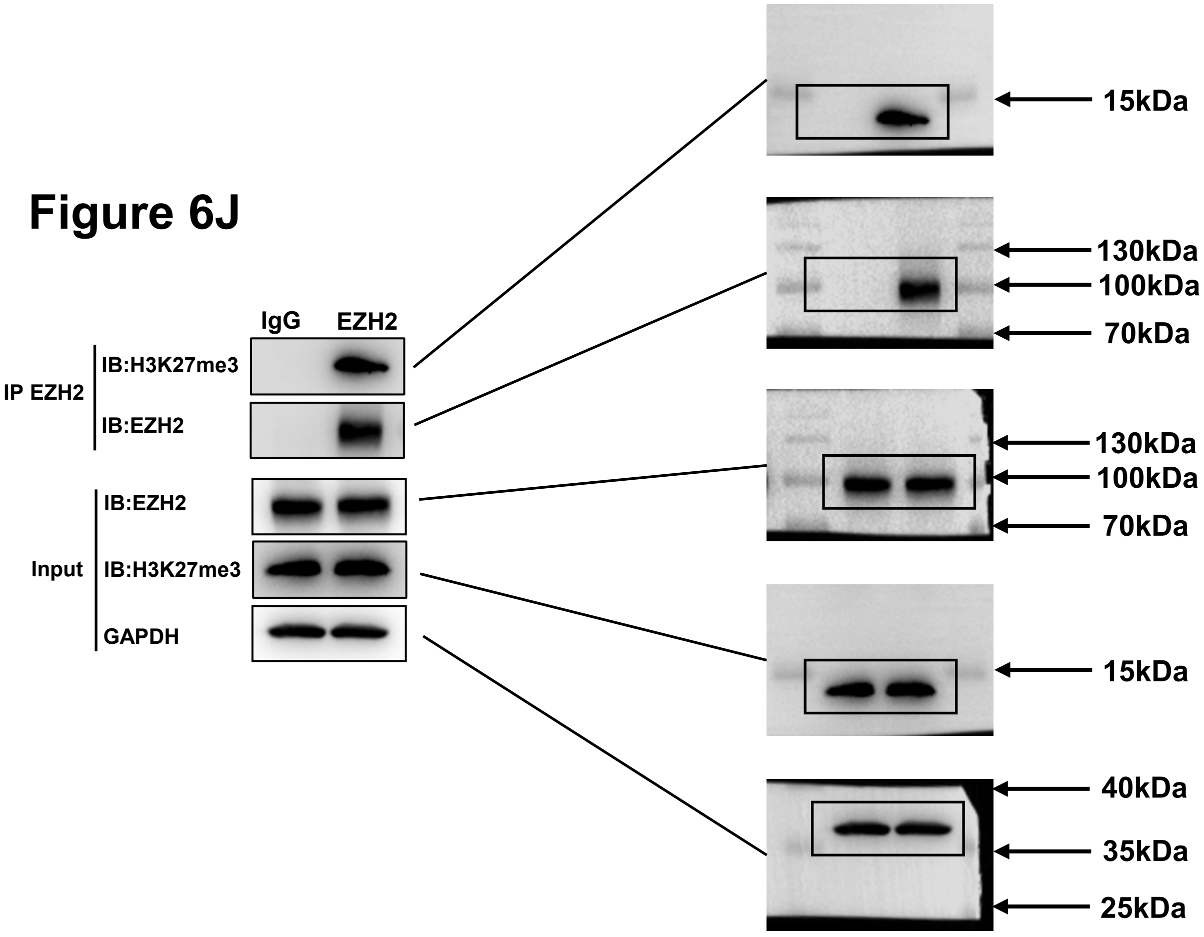

IgG
EZH2
IP EZH2
IB:H3K27me3
IB:EZH2
Input
IB:EZH2
IB:H3K27me3
GAPDH
15kDa
130kDa
100kDa
70kDa
130kDa
100kDa
70kDa
15kDa
40kDa
35kDa
25kDa

Figure 6L

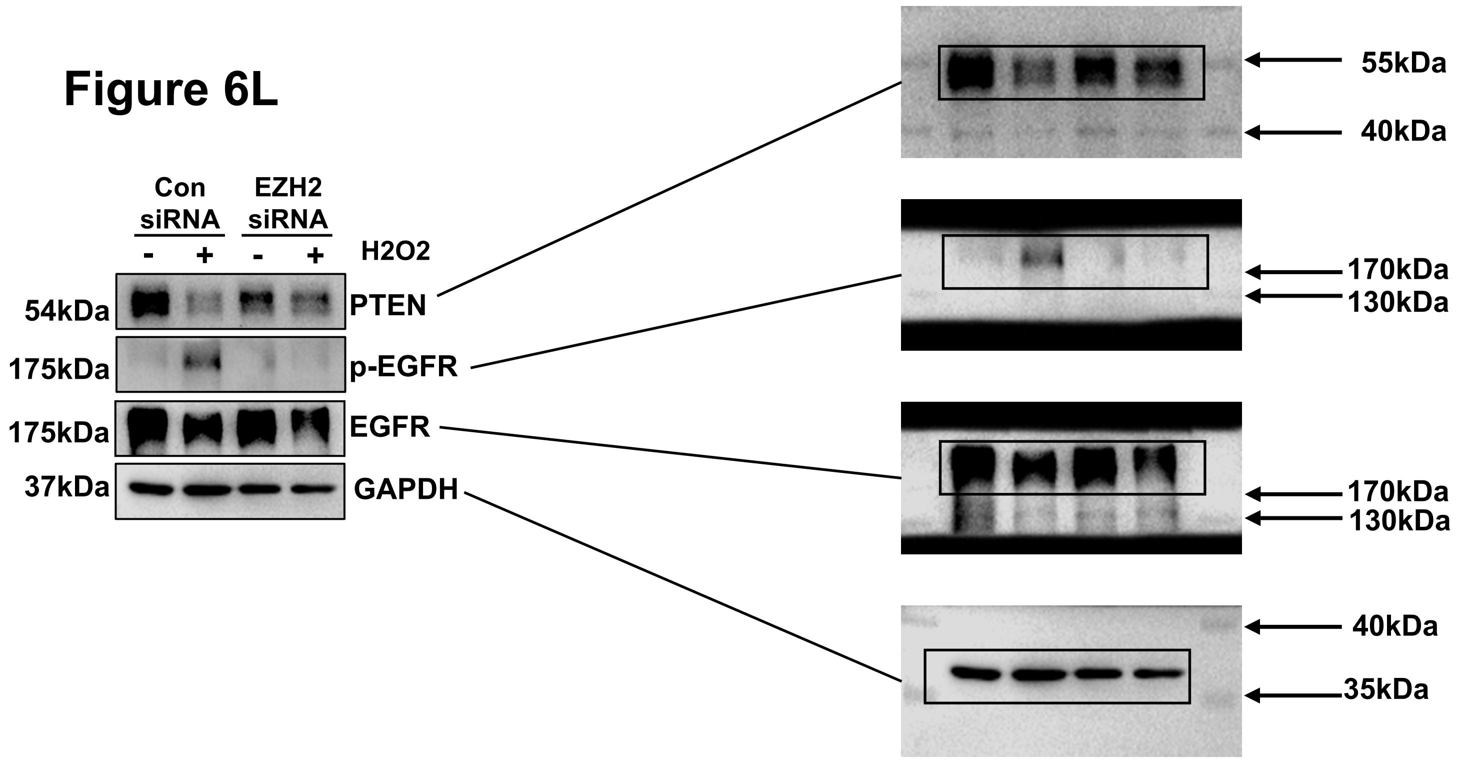

Figure 6P

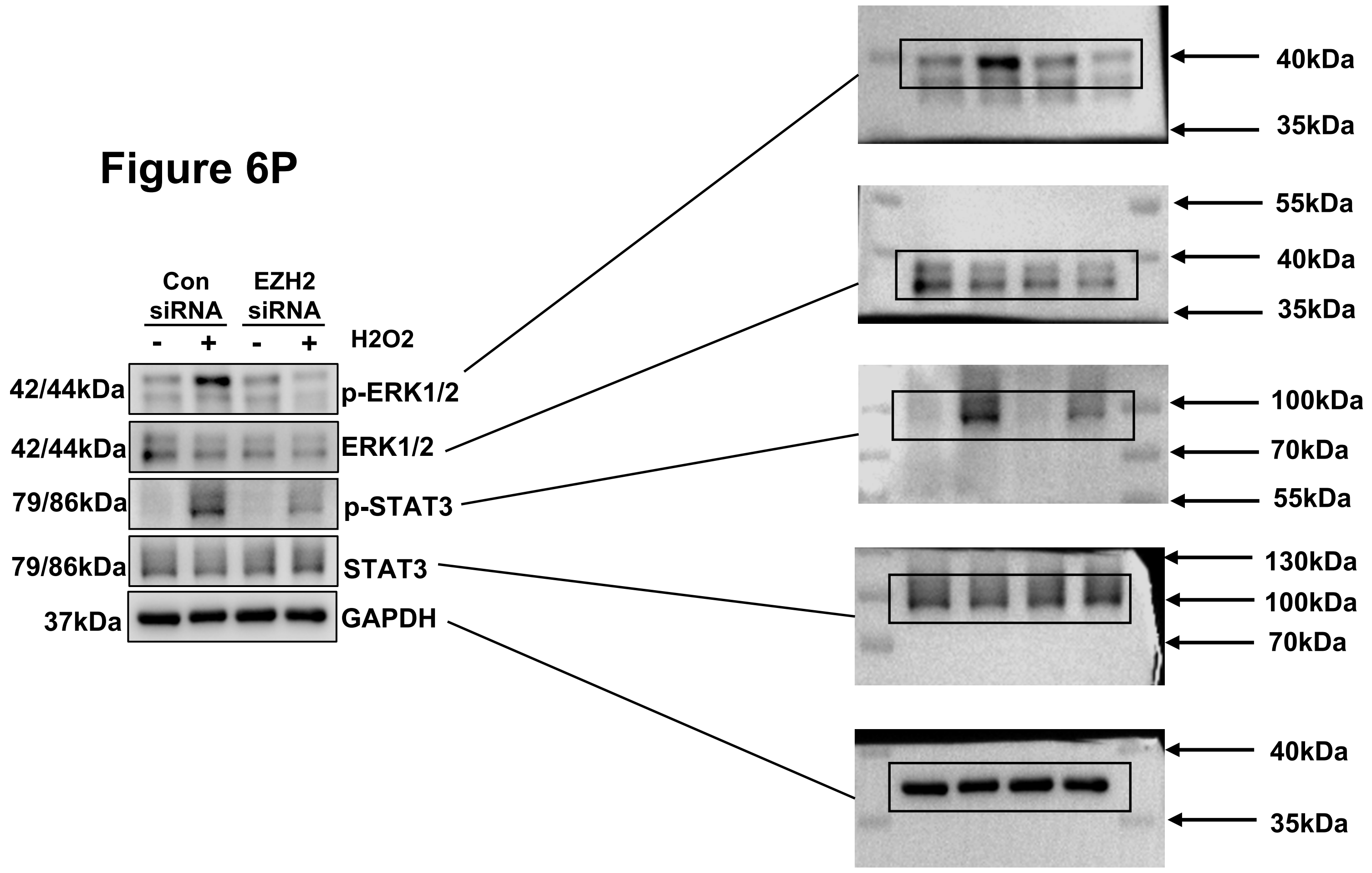

Figure 6S

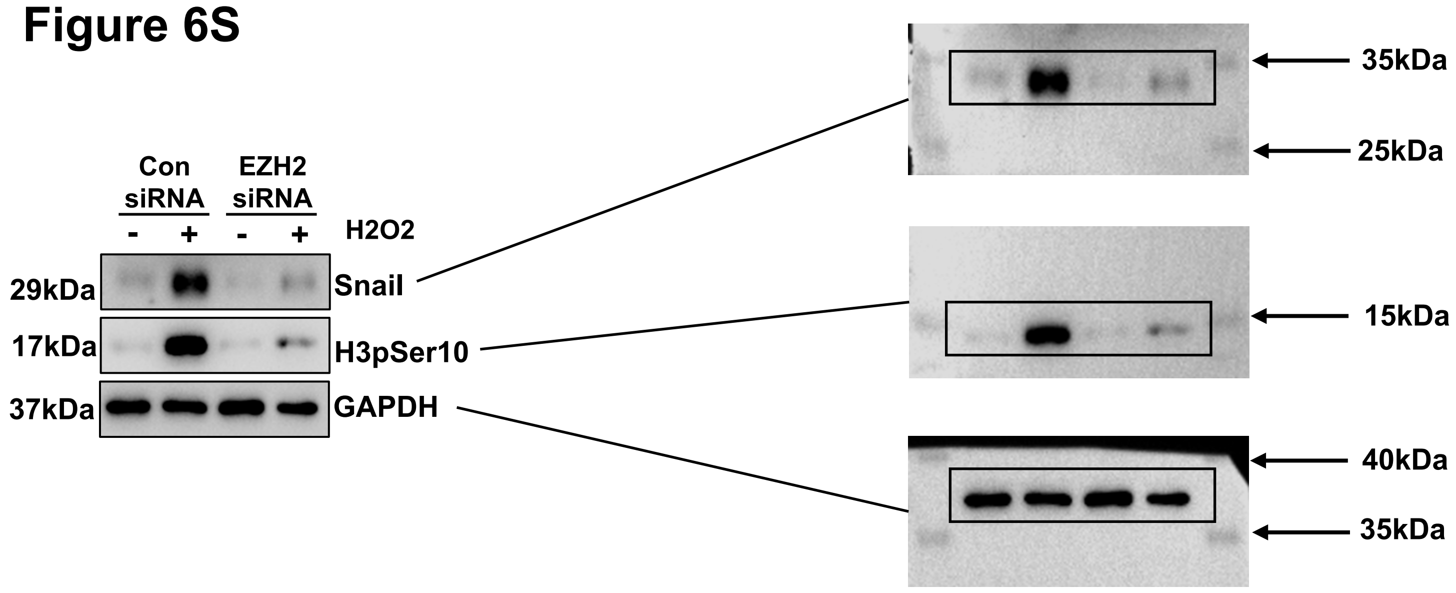

Figure 7A

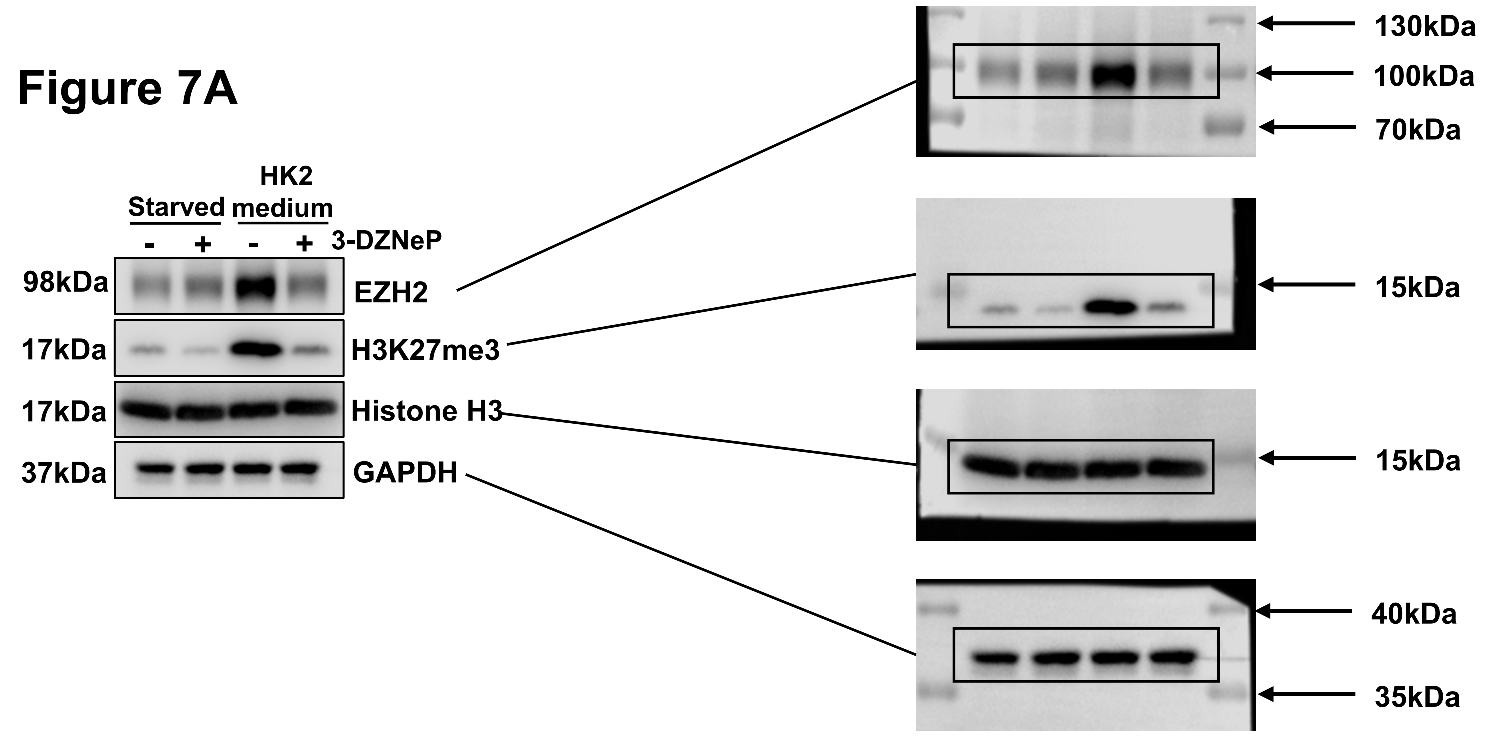

Figure 7D

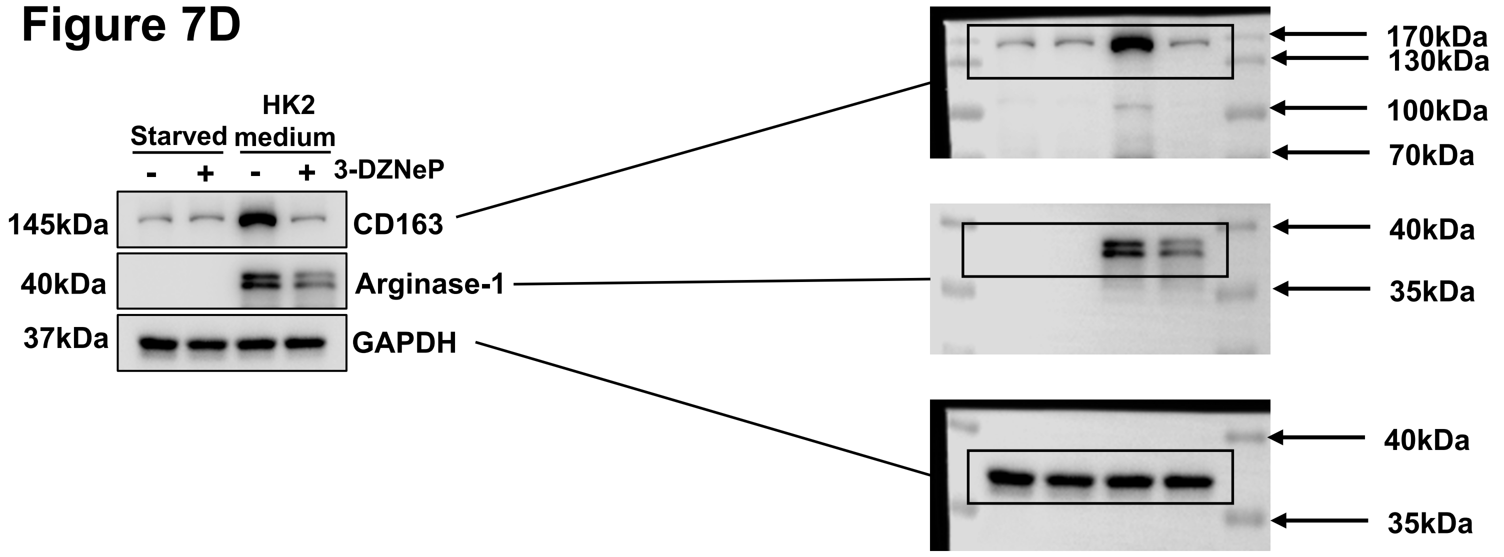

Figure 7G

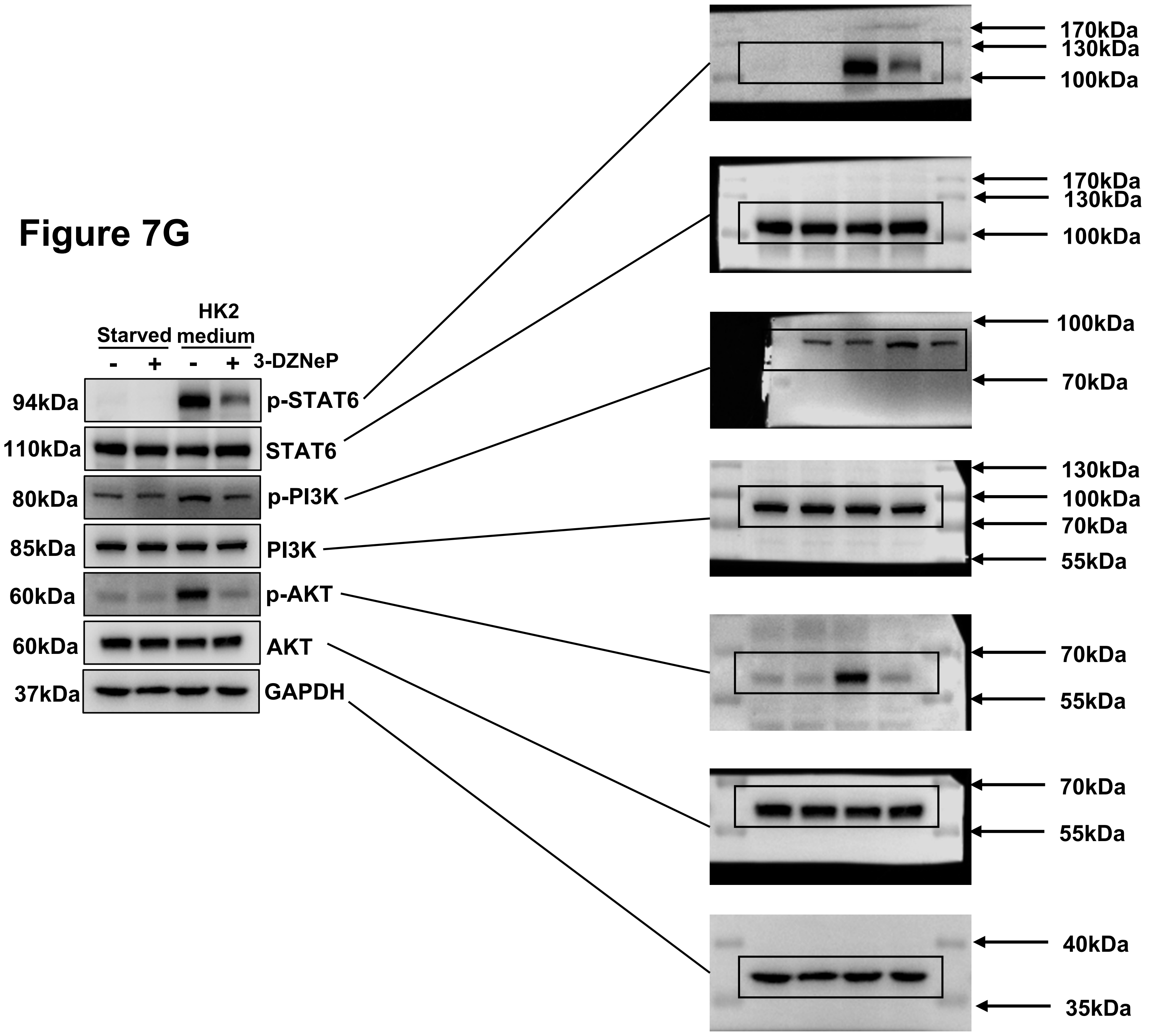

Figure 7K

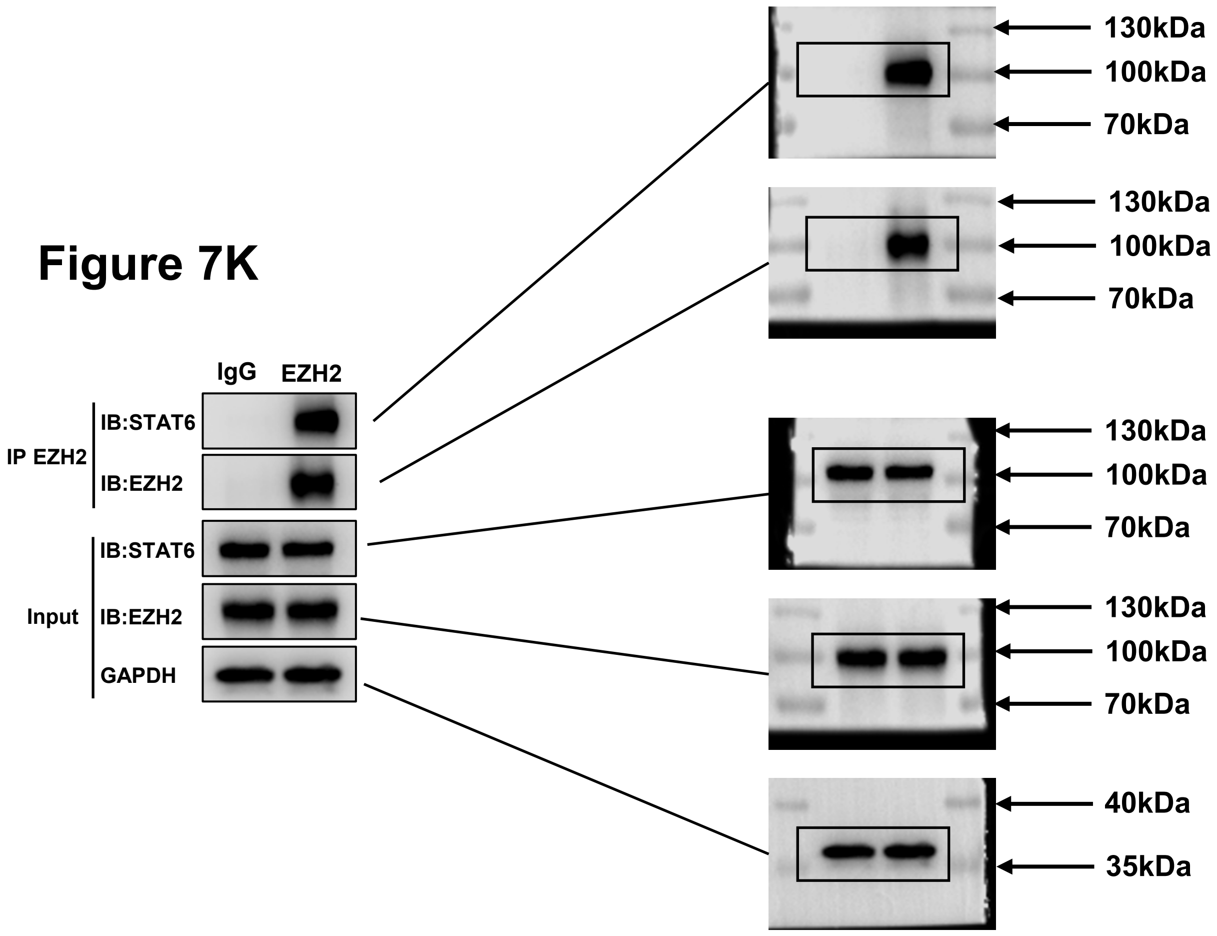
IgG
EZH2
IP EZH2
IB:STAT6
IB:EZH2
Input
IB:STAT6
IB:EZH2
GAPDH
130kDa
100kDa
70kDa
130kDa
100kDa
70kDa
130kDa
100kDa
70kDa
130kDa
100kDa
70kDa
40kDa
35kDa

Figure 7L

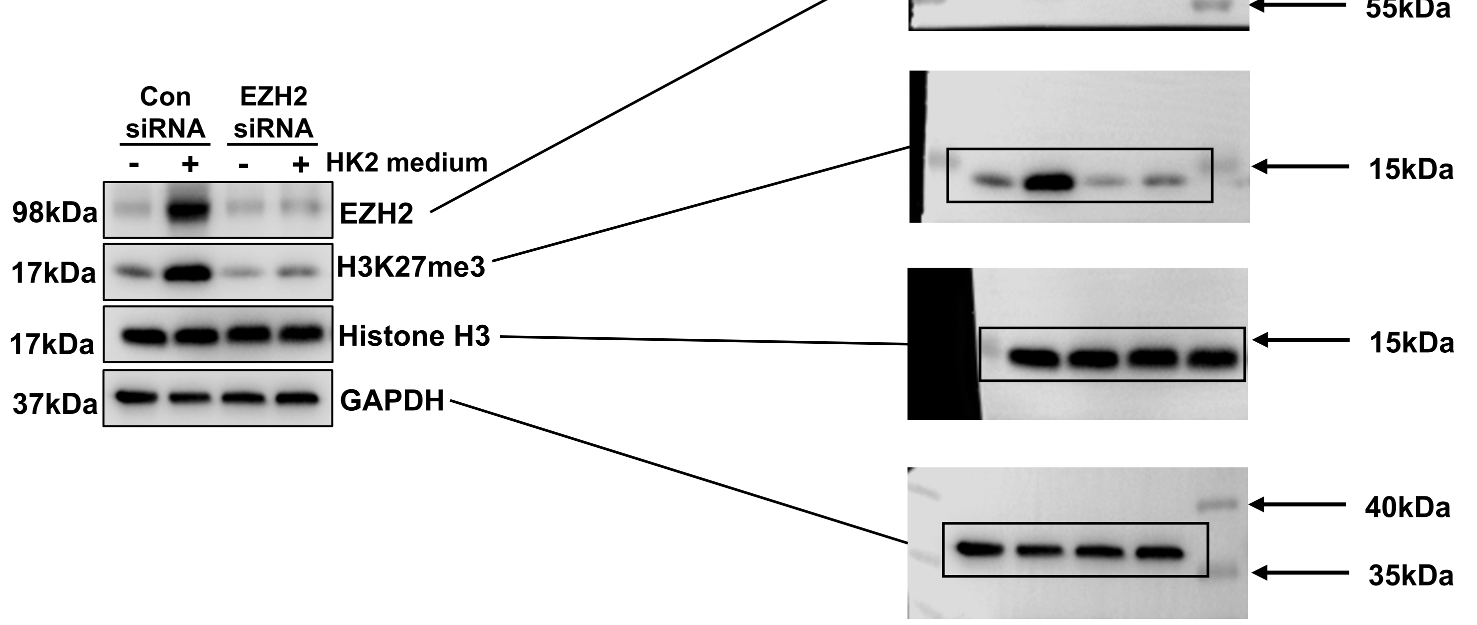

Figure 7O

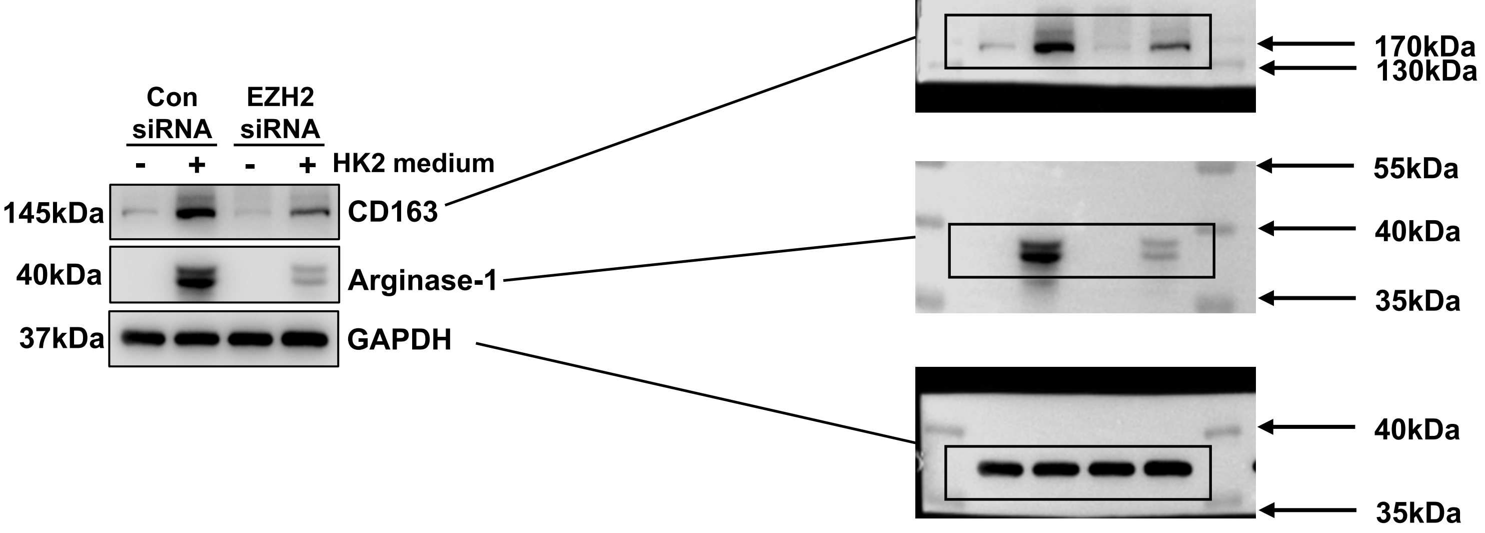

Figure 7R

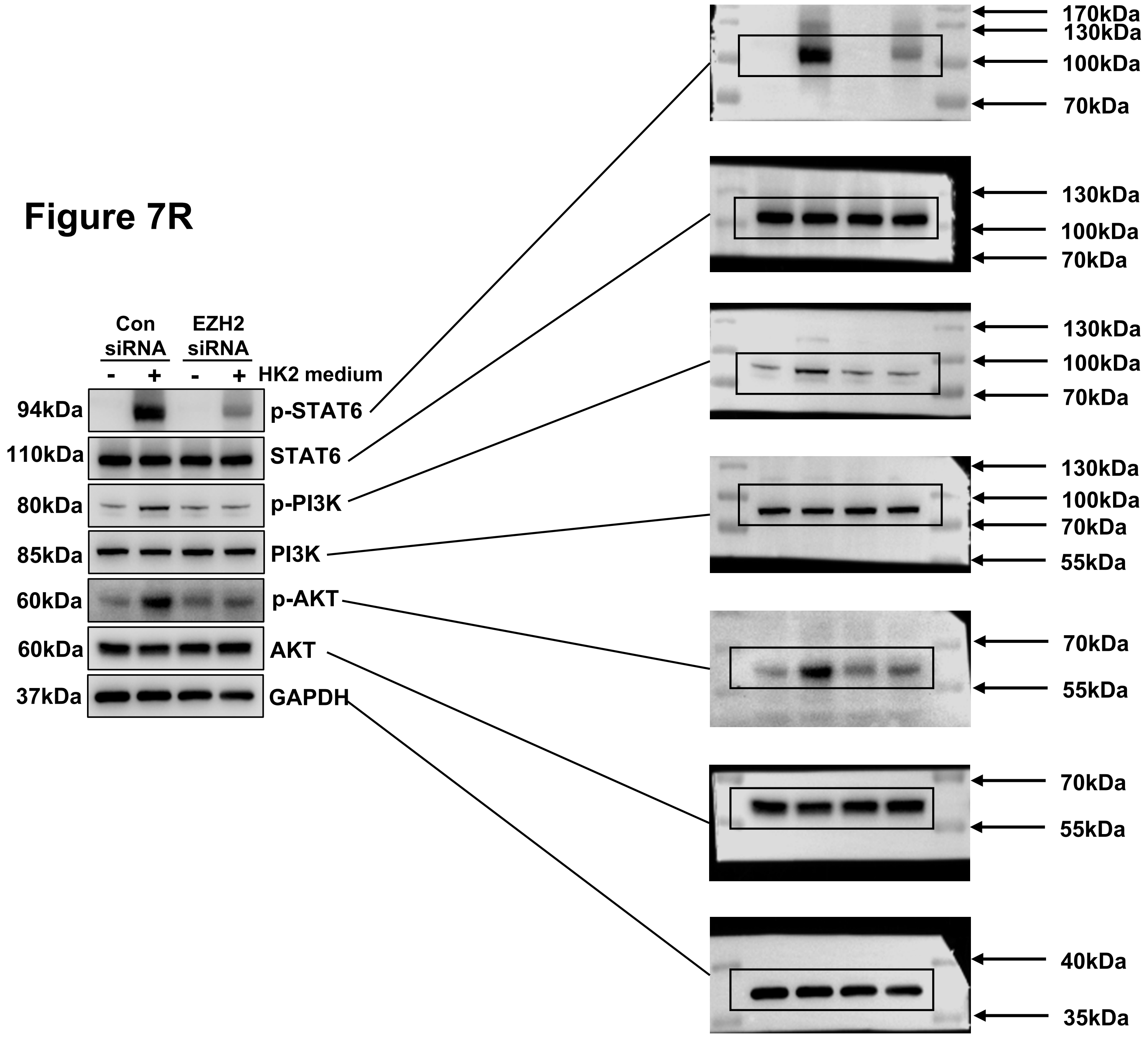

Figure 8B

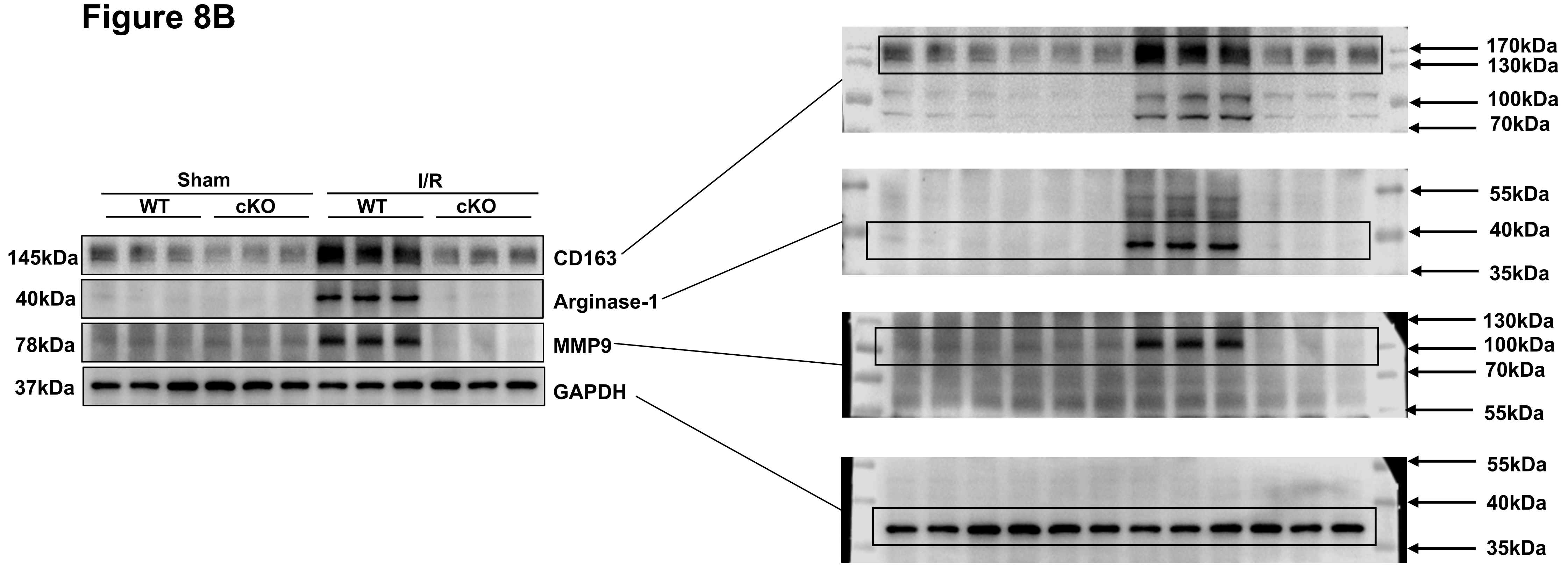

Figure 8F

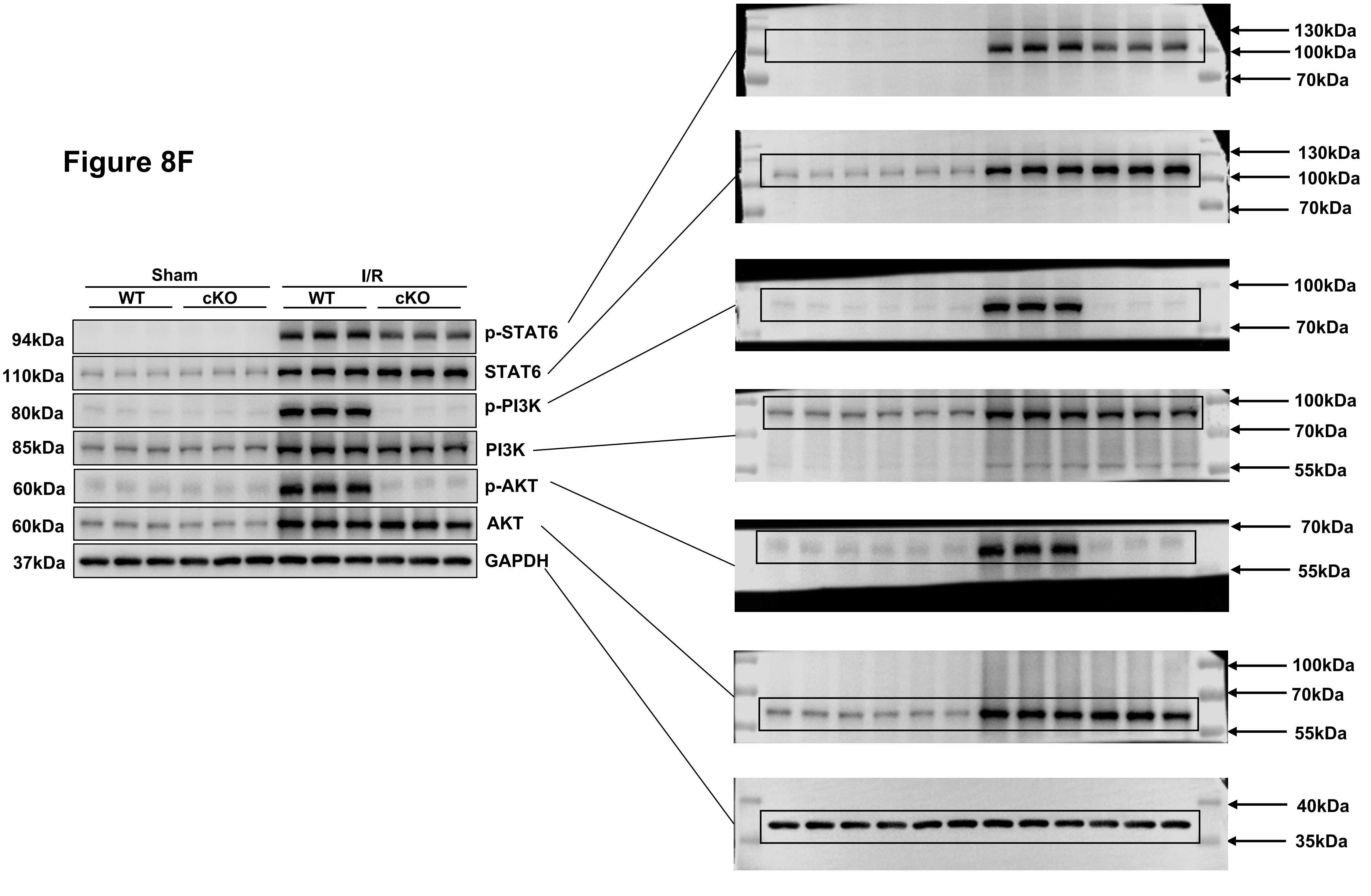

Figure 8J

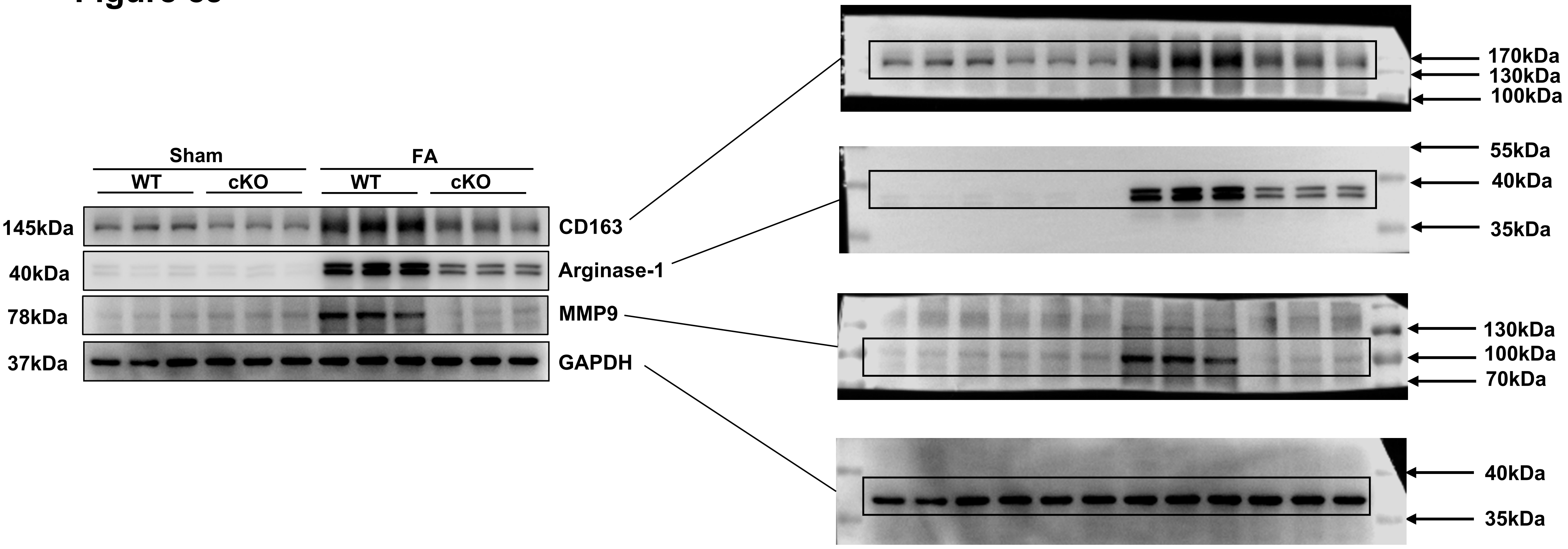

Figure 8K

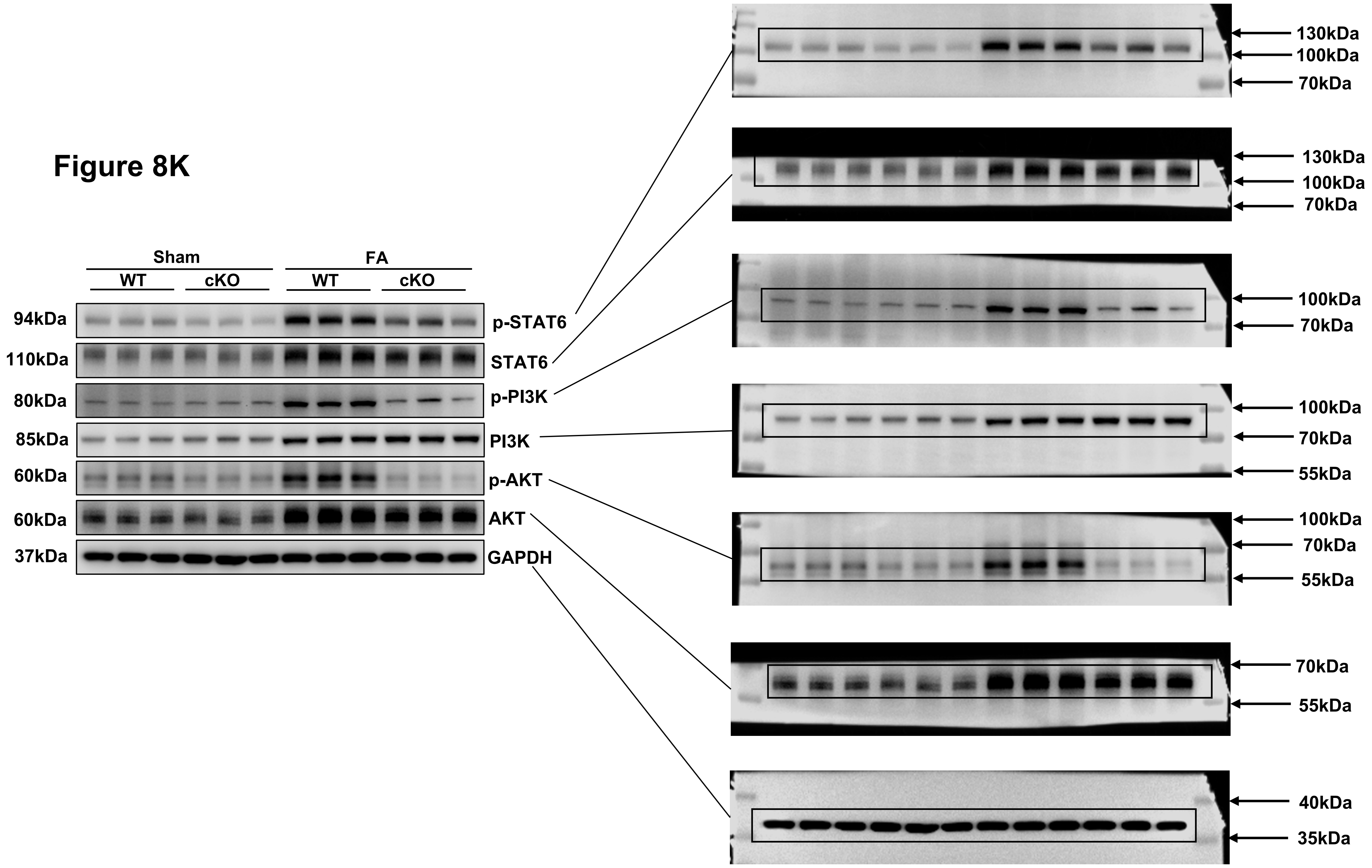

Figure S1E

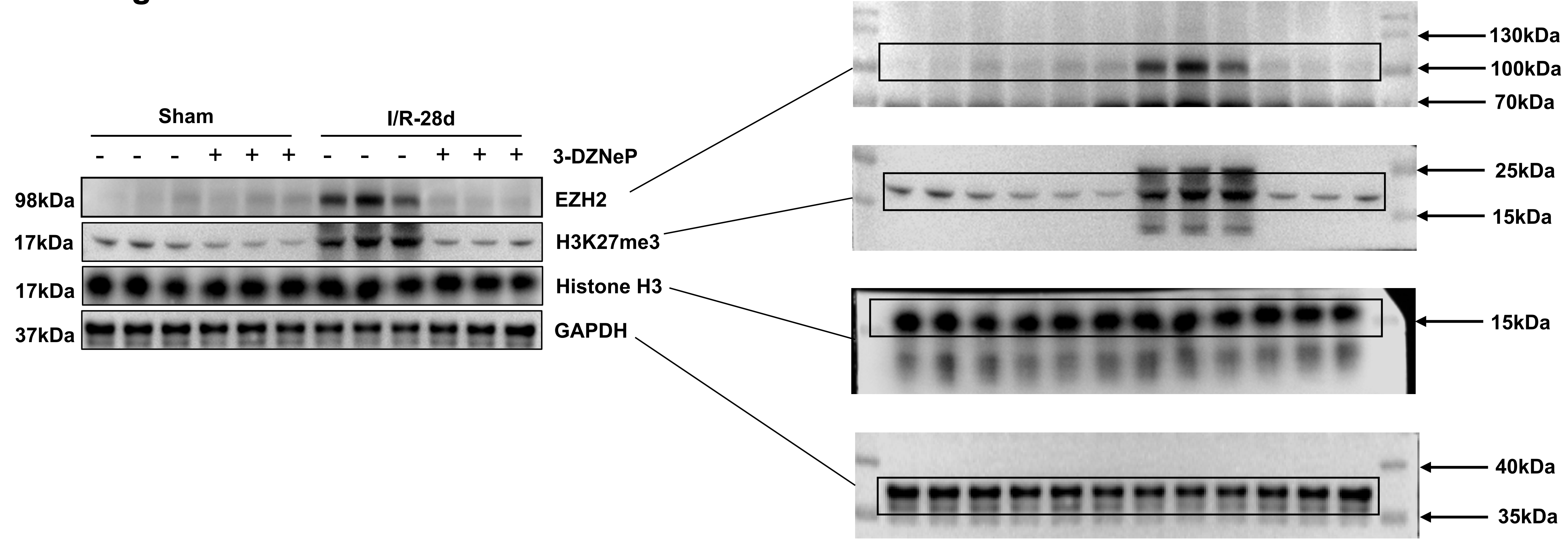

Figure S2C

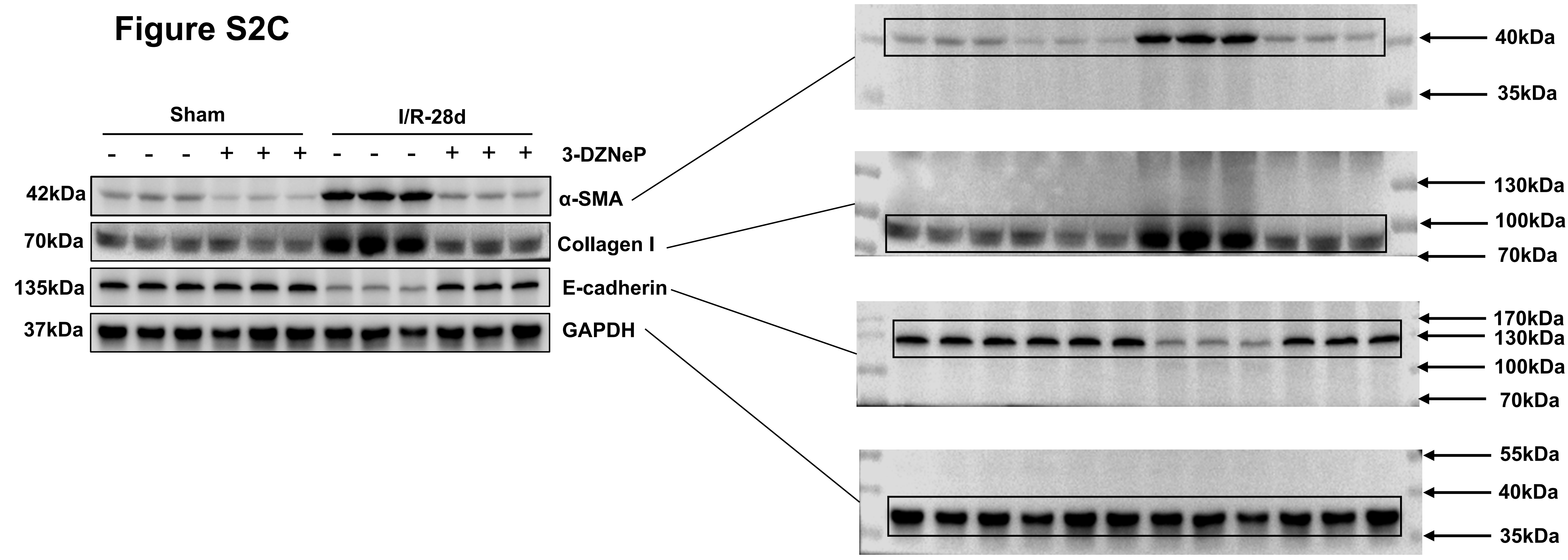

Figure S2H

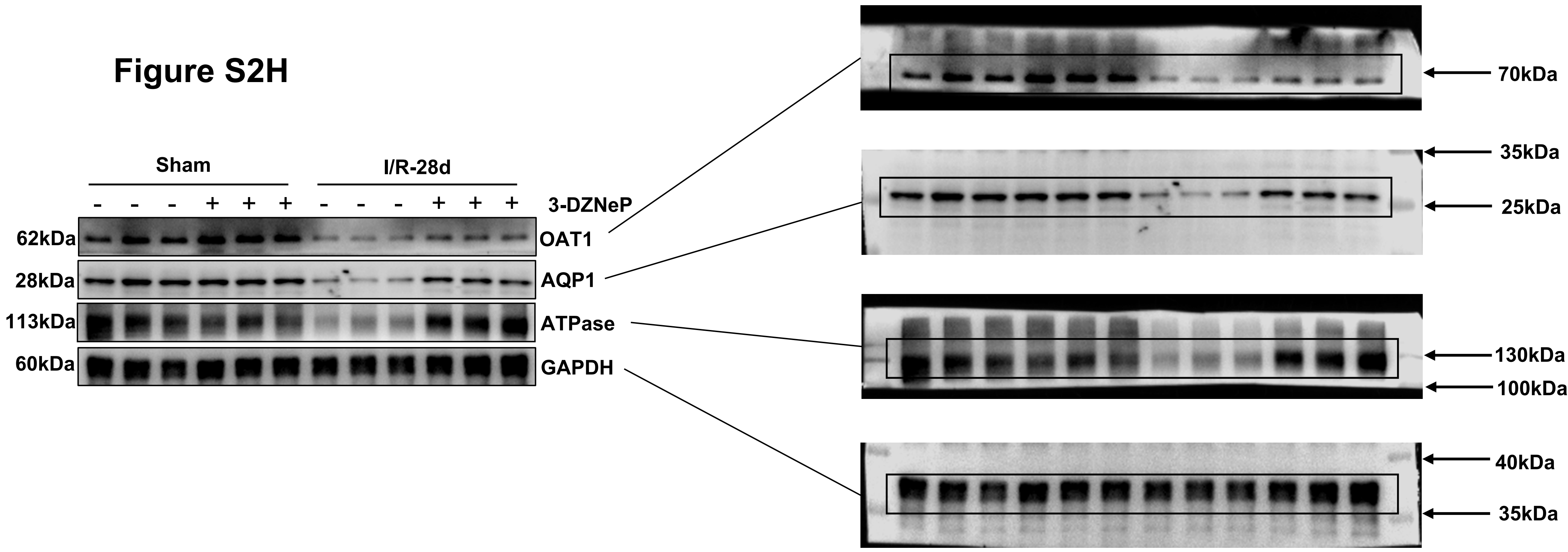

Figure S3F

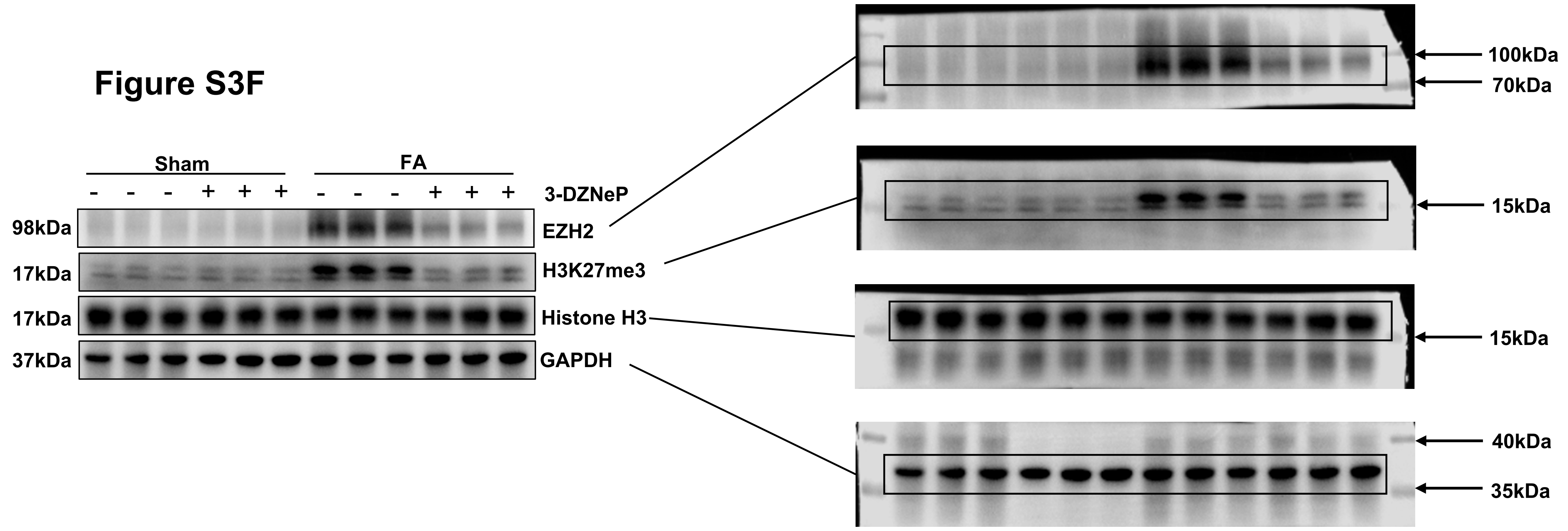

Figure S4C

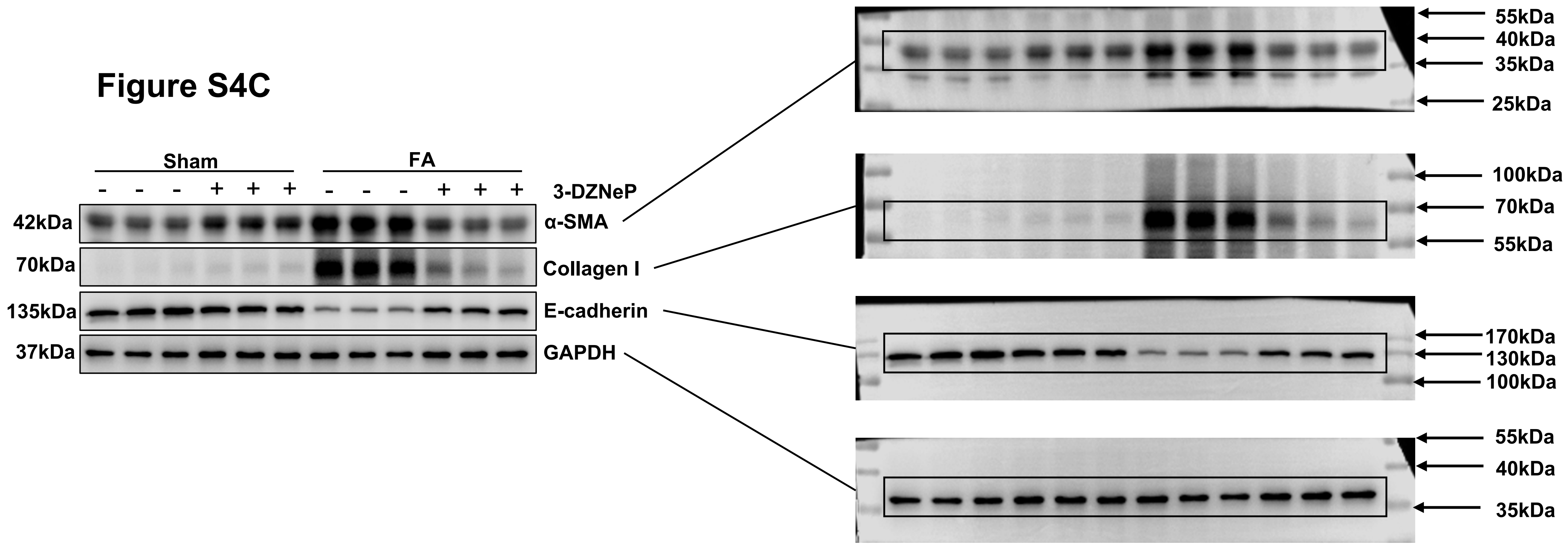

Figure S4G

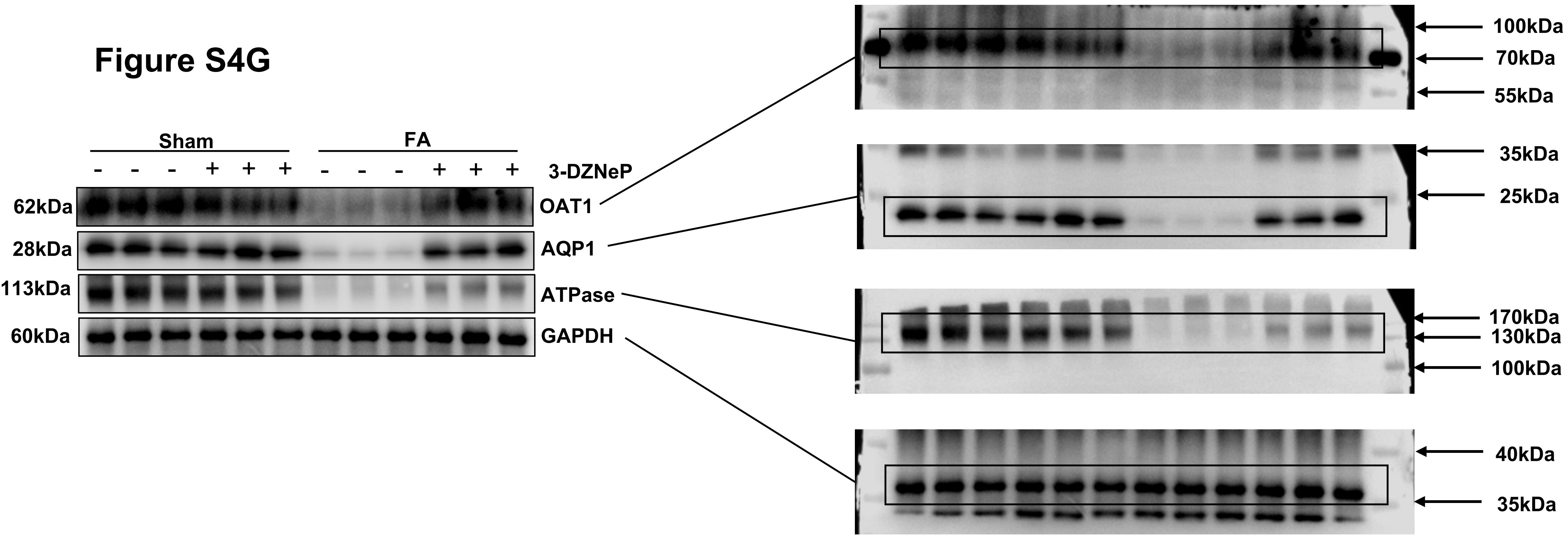

Figure S5A

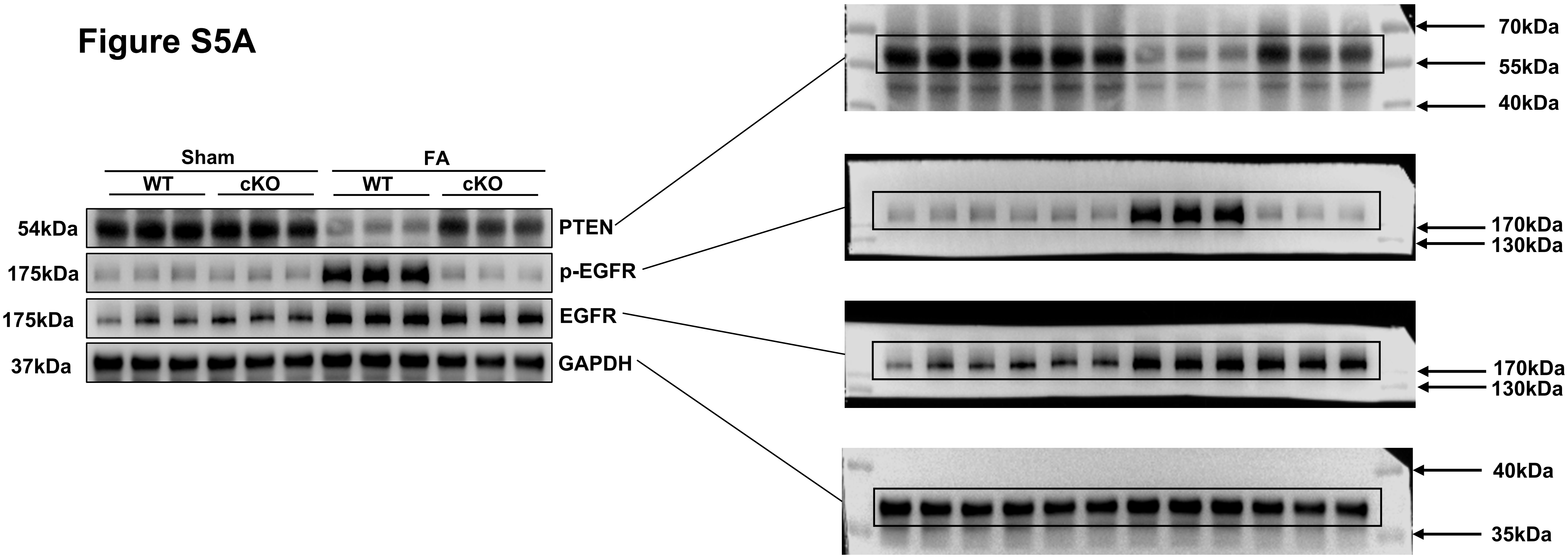

Figure S5E

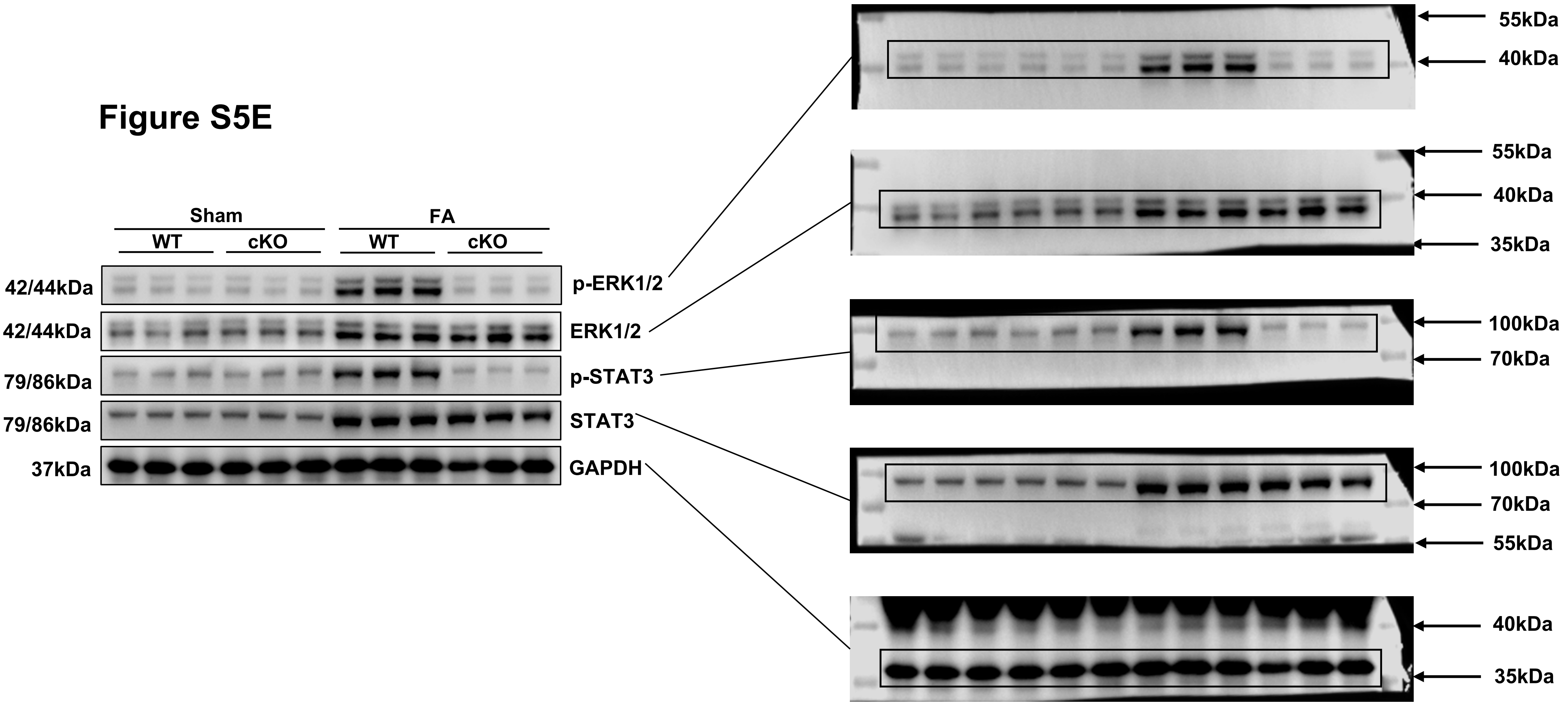

Figure S5J

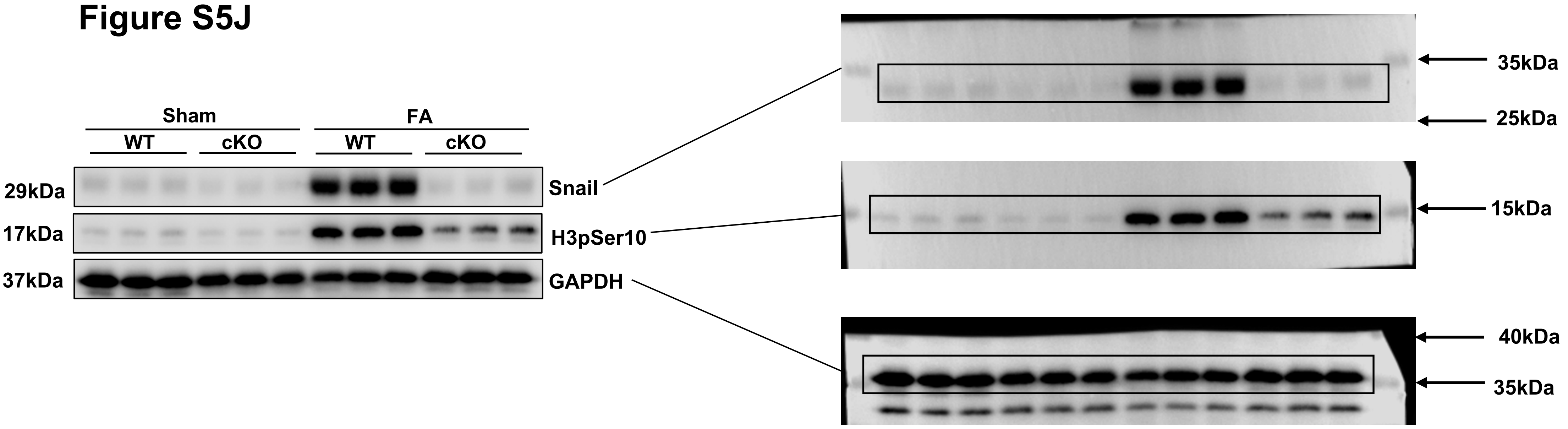

Figure S6A

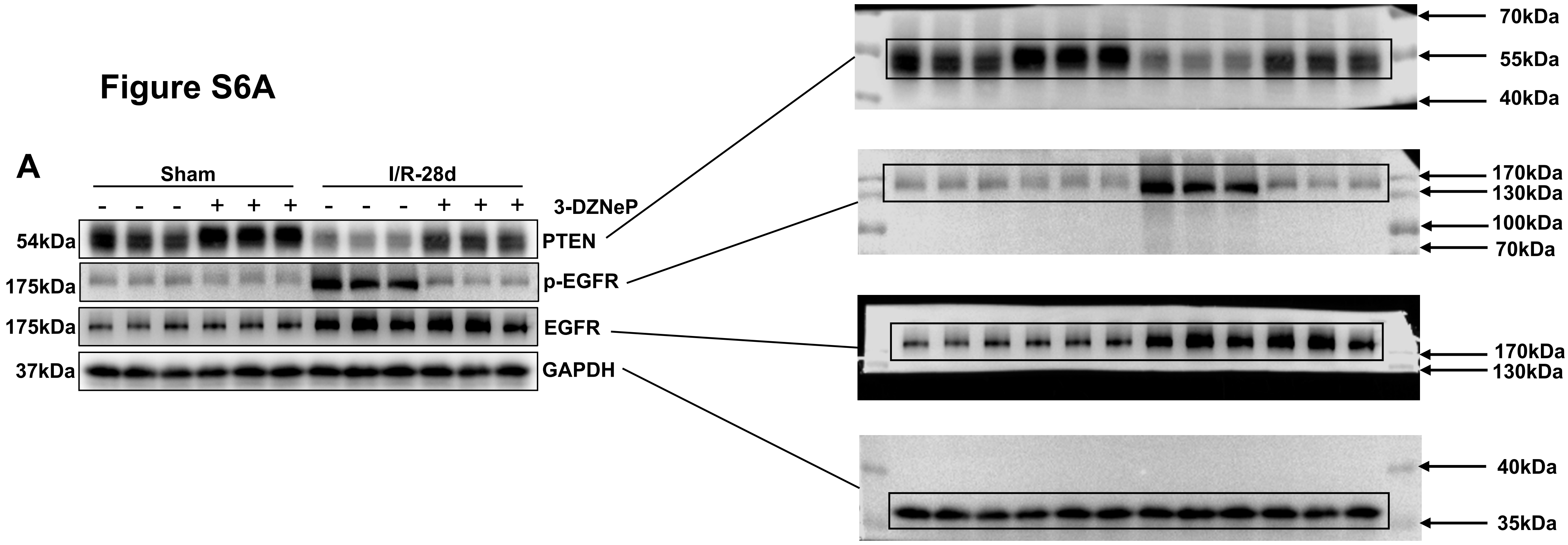

Figure S6D

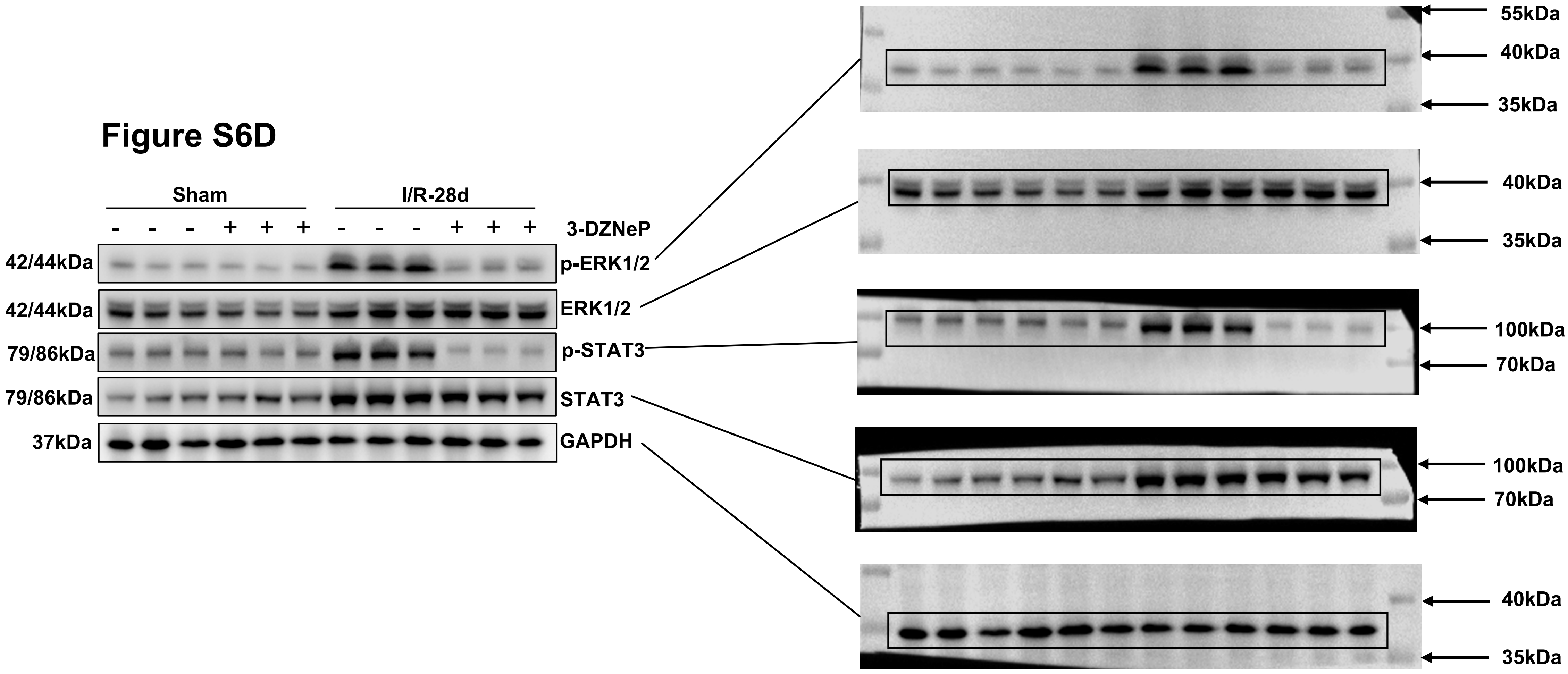

Figure S6G

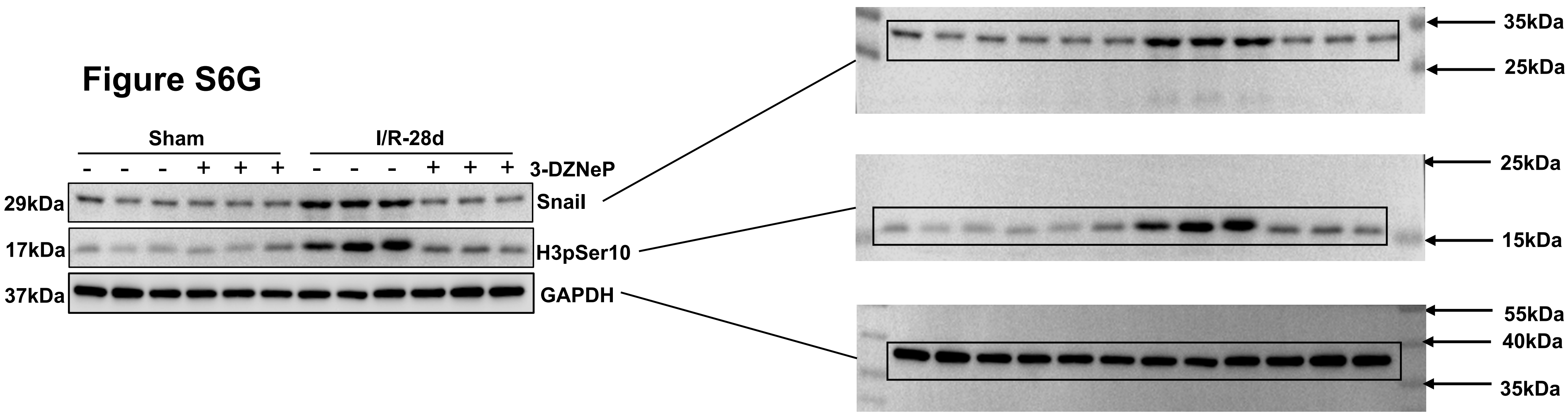

Figure S7A

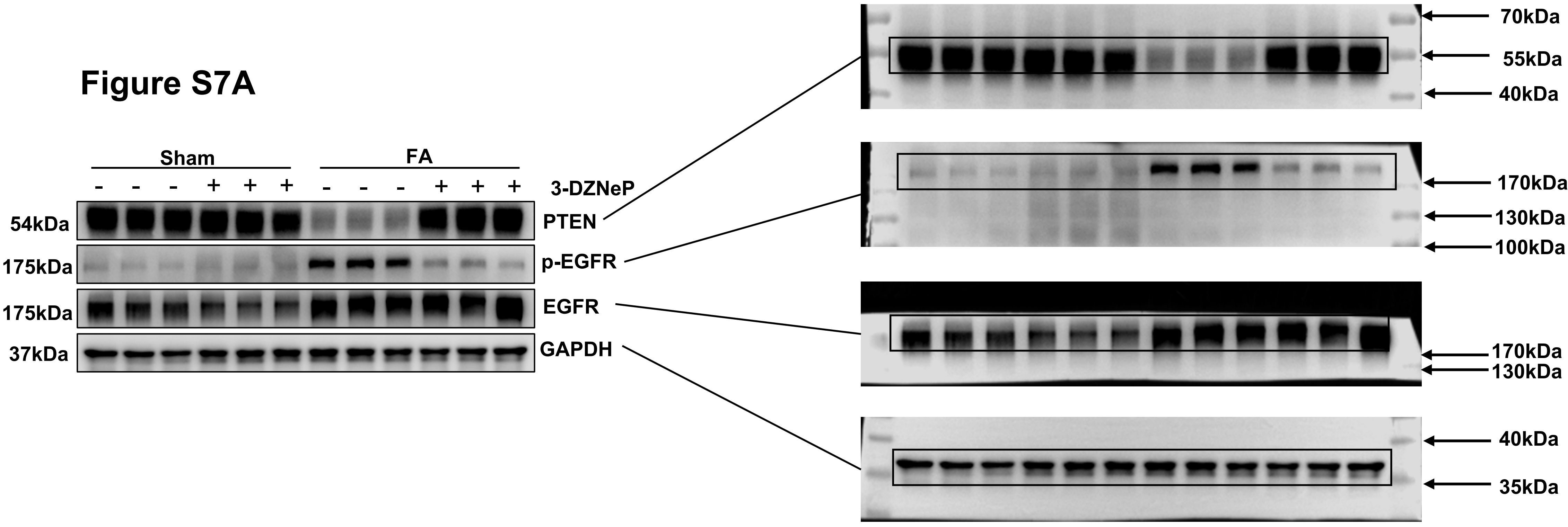

Figure S7D

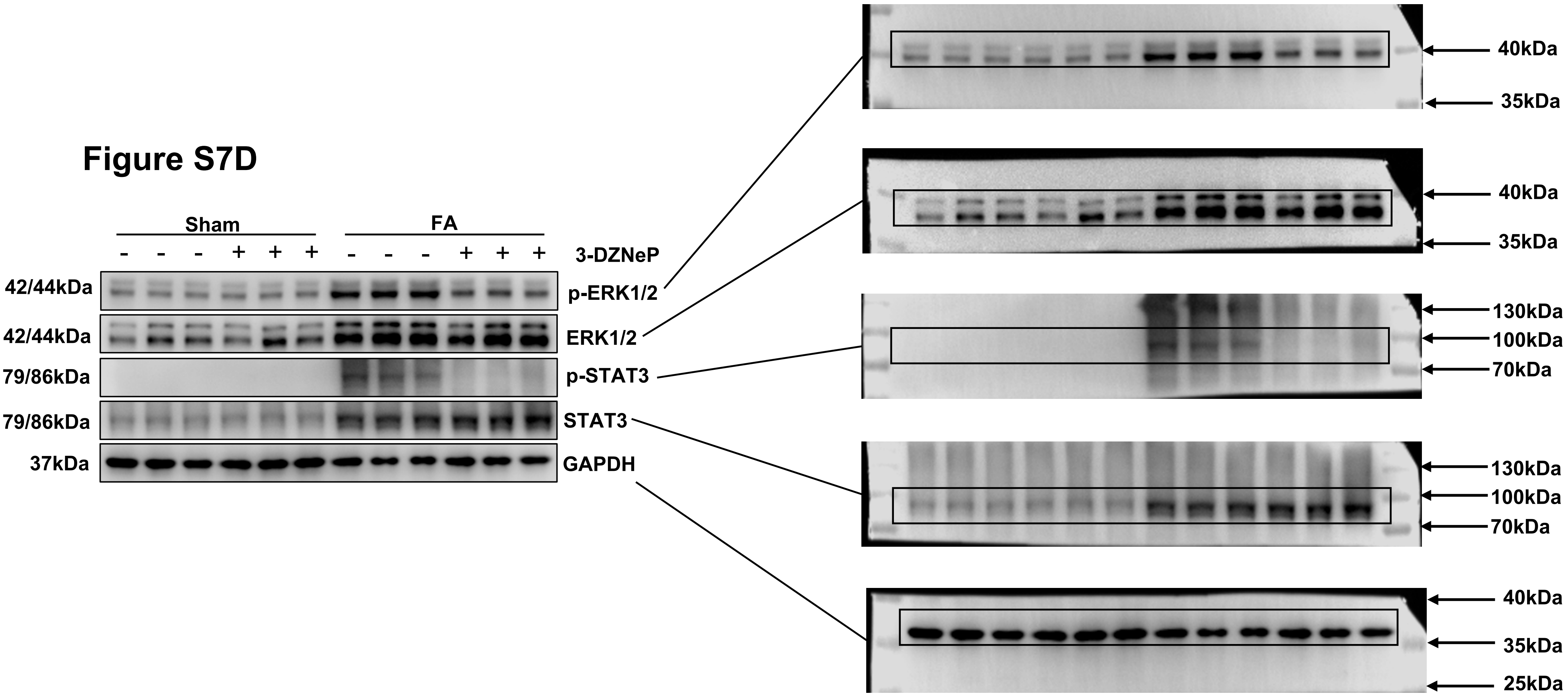

Figure S7G

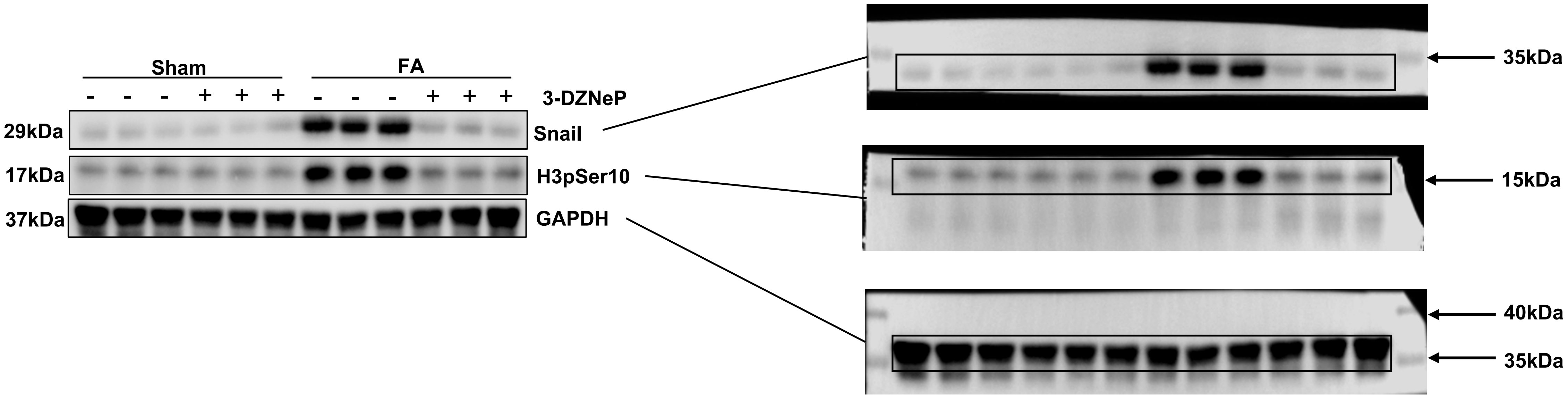

Figure S8A

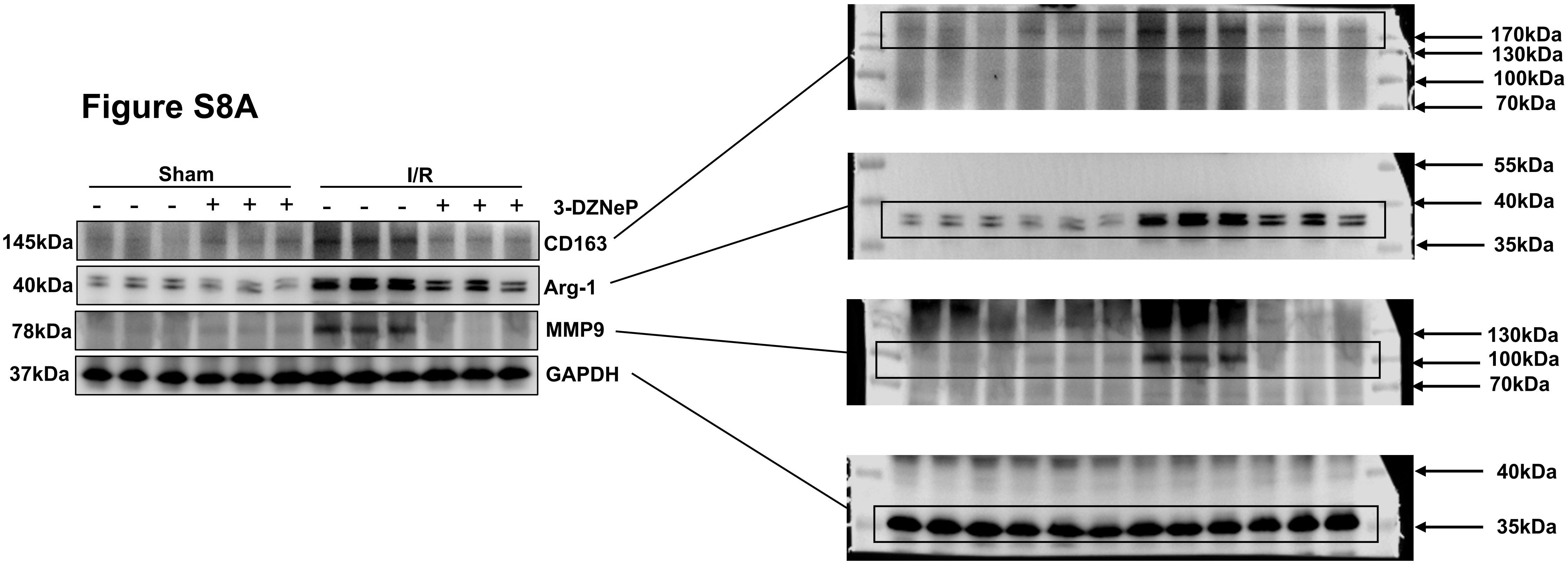

Figure S8E

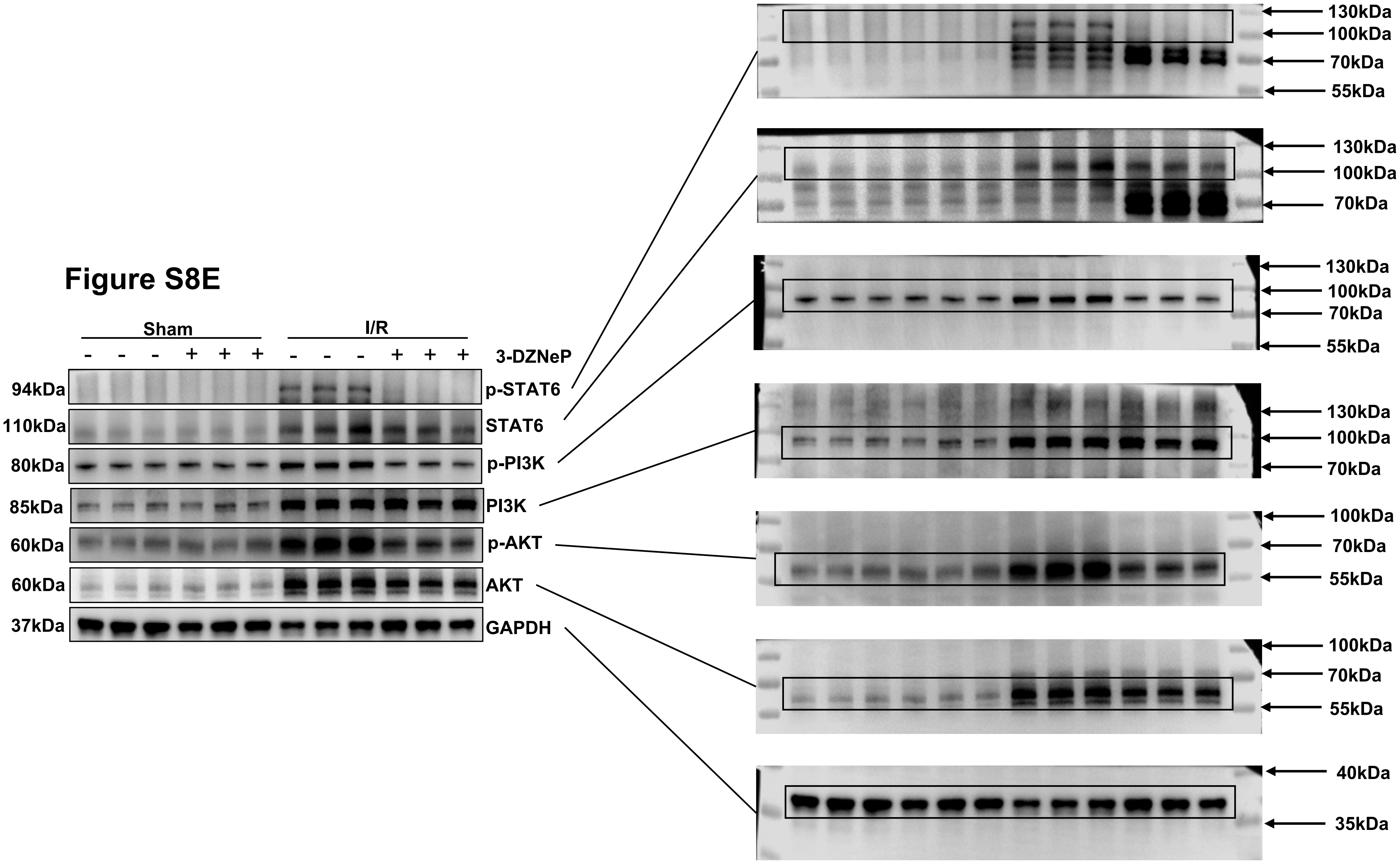

Figure S9A

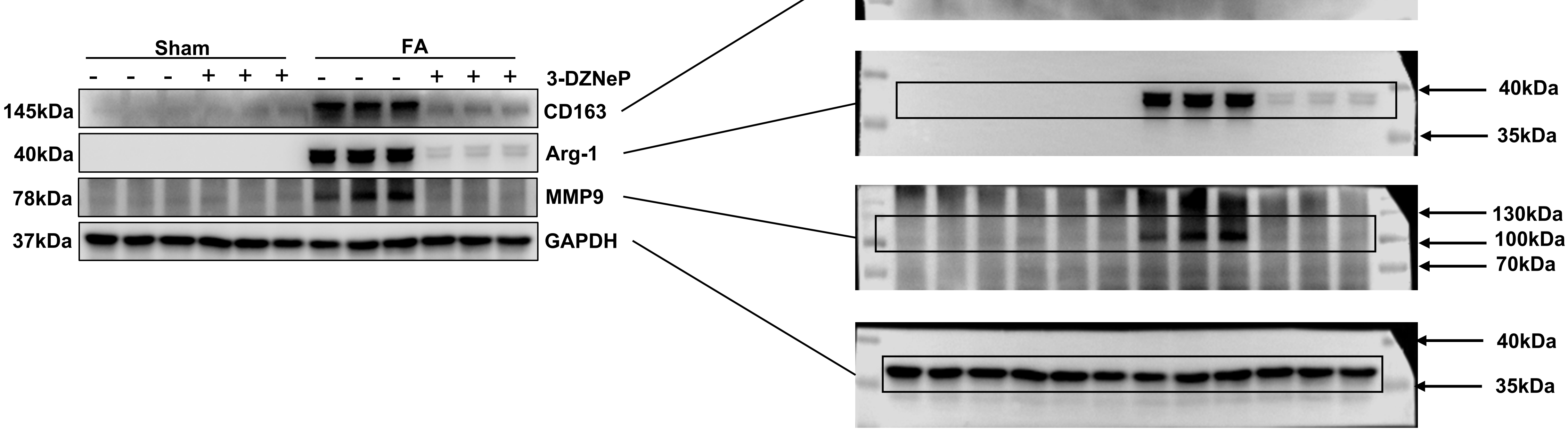

Figure S9E

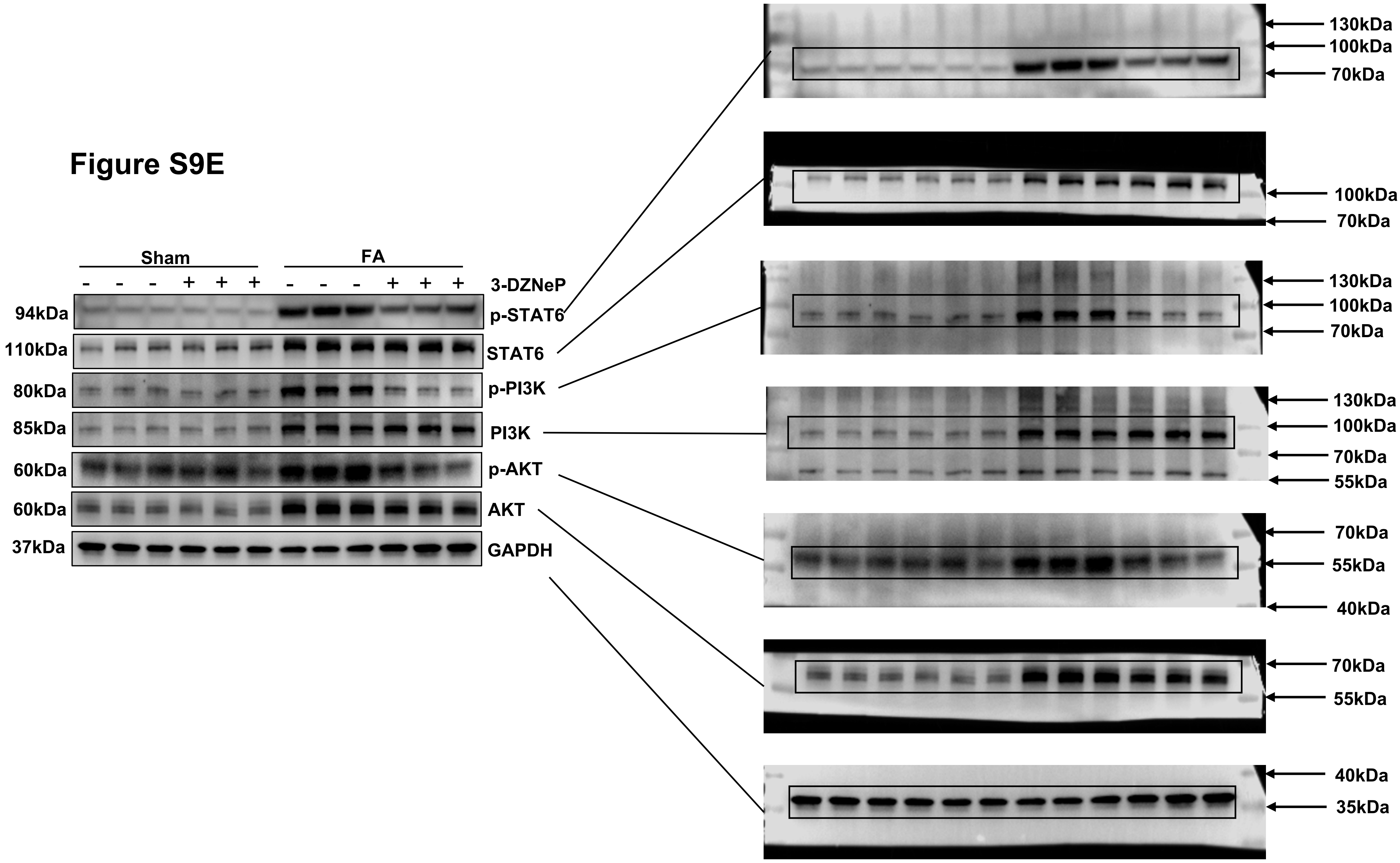

Supplement: Supplementary file 3 — Original Data File [file 41419_2023_5782_MOESM3_ESM.pdf]
